# Quasi-solitons in Rydberg atom chains

**DOI:** 10.1038/s41467-026-75598-1

**Published:** 2026-07-17

**Authors:** Aron Kerschbaumer, Jean-Yves Desaules, Marko Ljubotina, Maksym Serbyn

**Affiliations:** 1 Institute of Science and Technology Austria (ISTA), Klosterneuburg, Austria; 2 Physics Department, Technical University of Munich, TUM School of Natural Sciences, Garching, Germany; 3 Munich Center for Quantum Science and Technology (MCQST), München, Germany

**Keywords:** Quantum simulation, Statistical physics, thermodynamics and nonlinear dynamics

## Abstract

Solitons—localized wave packets that travel without spreading—play a central role in understanding transport and properties of nonlinear systems. In quantum many-body systems, however, such robust excitations are typically destroyed by thermalization. Here, we theoretically demonstrate the existence of solitonic excitations in high-energy states of Rydberg atom chains in the regime of strong nearest-neighbor Rydberg blockade. These localized wave packets propagate directionally atop a special class of reviving initial states related to quantum many-body scars and are capable of carrying energy. Exhibiting long coherence times, these states constitute a form of non-ergodic quantum dynamics and can be efficiently implemented on Rydberg atom simulators. In this work, in addition to a phenomenological description of solitons, we identify their counterpart in a classical nonlinear dynamical system, demonstrate their potential use in quantum information transfer, and conjecture their relevance for anomalous energy transport reported in numerical studies of Rydberg atom arrays.

## Abstract (web summary)

This work identifies long-lived chiral quasiparticles emerging on top of periodically reviving scarlike states in a Rydberg atom chain at high energy densities where instead thermalization is expected. These quasiparticles enable ballistic energy transport, coherent information transfer, and have direct non-trivial analogues in a corresponding nonlinear classical system while far from the semiclassical limit.

## Introduction

Coherently propagating solitons in nonlinear systems have been realized in numerous classical and quantum platforms, ranging from water waves and optical fibers to Bose-Einstein condensates^1^, magnetic materials^2^, and superconducting circuits^3^. While many of these realizations occur in continuum media, modern experimental advances enable the study of discrete solitons, realized in particular by arrays of optical waveguides and similar systems^4^. In quantum settings, soliton excitations are known to exist in numerous integrable lattice models^5,6^, yet their experimental study is currently limited to dynamics of isolated magnon bound states, which can be regarded as the basic building blocks of magnetic solitons^7^.

In our work, we establish the existence of quasi-solitons in the PXP model^8,9^, a strongly interacting, chaotic system that corresponds to an approximate description of Rydberg atom arrays^10–12^. They exist in high-energy states of the PXP model and can be efficiently prepared and launched in Rydberg atom simulators. Specifically, we show that quasi-soliton excitations can be created as simple defects on top of homogeneous short-range entangled states that feature coherent revivals^11,12^.

**Fig. 1:**
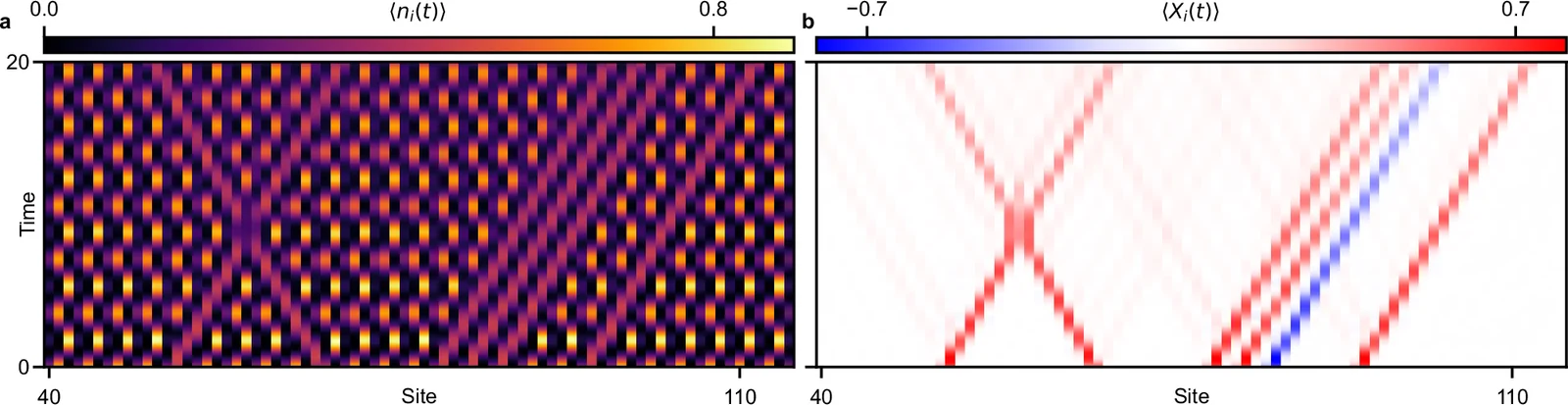
Dynamics of an energy-carrying multi-soliton state. Solitons are embedded as local defects in the weakly entangled scarred state $\left| K\right\rangle$. The solitonic defects are propagating unidirectionally atop the oscillating scar background, as seen in the evolution of **a**, number operator density and **b**, energy density. The collision leads to additional soliton decay. Details of the numerical simulation and the exact form of the states are provided in the Methods section.

These quasi-solitons can carry energy, set by a continuous internal degree of freedom. Multiple quasi-solitons can also be created. This only weakly affects their coherent propagation, except for collisions, which lead to partial decay. Their imperfect nature is in line with the non-exact revivals of the scarred initial states^13,14^ they are built upon. This approximate scarred dynamics in a chaotic system makes it difficult to rigorously relate our coherently propagating quasiparticles to solitons in the strict sense. Therefore, we call them quasi-solitons, and establishing a rigorous connection will be a subject of future work. For brevity, we refer to them simply as solitons for the rest of the manuscript; we return to a more detailed discussion of their quasi-solitonic character in the Discussion. These multi-soliton states span an exponentially large manifold of non-thermal, experimentally relevant states. The dynamics of a representative state is shown in Fig. 1 via local observables—the number operator density **a** and energy density **b**—showing uniform soliton propagation on top of the oscillating background. The exponential proliferation of long-lived energy-carrying solitons suggests that they may underlie the unconventional energy transport observed in the PXP model^15^ and points to potential applications in quantum-information protocols, which we will demonstrate by launching a Bell pair of oppositely moving solitons, generating long-range entanglement in an experimentally accessible quench. In addition to the phenomenological construction of solitons, we also identify their counterparts in a classical dynamical system, establishing a connection to solitons in classical nonlinear systems.

## Results

### Model and properties

The dynamics of a Rydberg atom chain can be described as an effective two-level system in which the ground state $\left |\downarrow \right\rangle$ is coupled to the excited state $\left| \uparrow \right\rangle$ via driving with a homogeneous Rabi frequency Ω ^10–12^. Atoms in excited states $\left| \uparrow \right\rangle$ interact with rapidly decaying van der Waals interactions. By tuning an appropriate inter-atom distance, the chain can be brought to the regime of nearest-neighbor Rydberg blockade, characterized by strong nearest-neighbor interactions and negligible interactions beyond. Such an *N*-atom Rydberg chain with nearest-neighbor blockade and periodic boundary conditions can be approximated by the so-called PXP model with the Hamiltonian^9,11^:

1 $$H={\sum }_{i=1}^{N}{P}_{i-1}{X}_{i}{P}_{i+1}.$$

The projectors onto the ground state ${P}_{i}={\left(\left| \downarrow \right\rangle \left\langle \downarrow \right| \right)}_{i}$ enforce a kinetic constraint by preventing neighboring atoms from occupying the excited state simultaneously. The Pauli matrix ${X}_{i}={\left(\left| \downarrow \right\rangle \left\langle \uparrow \right|+\left| \uparrow \right\rangle \left\langle \downarrow \right| \right)}_{i}$ creates Rabi oscillations between the ground and excited state. The model obeys a global chiral symmetry that will play an important role below, generated by an operator ${{{\mathcal{C}}}}$ that upon conjugation changes the sign of the Hamiltonian:

2 $${{{\mathcal{C}}}}=\mathop{\prod }_{i=1}^{N}{Z}_{i},\qquad {{{\mathcal{C}}}}H{{{\mathcal{C}}}}=-H,$$

with ${Z}_{i}={\left(\left| \uparrow \right\rangle \left\langle \uparrow \right| -\left| \downarrow \right\rangle \left\langle \downarrow \right| \right)}_{i}$. The PXP model has been intensively studied due to the presence of quantum many-body scars^16–18^—non-ergodic eigenstates embedded in its otherwise thermal spectrum^13,19–22^. In a quantum quench setup, when one follows the unitary evolution generated by Eq. (1) from simple initial states, quantum many-body scars are manifested in approximate periodic revivals for a few, but not generic, initial states. Intuitively, such scarred initial states are located on the periodic trajectory of an effective semiclassical description of the dynamics, and therefore lead to revivals in a quantum quench^23,24^. The most studied scarred initial state is the Néel product state $\left| {{\mathbb{Z}}}_{2}\right\rangle=\left| \downarrow \uparrow \downarrow \uparrow \ldots \right\rangle$ constructed by repeating the unit cell of two atoms, $\left| \downarrow \uparrow \right\rangle$^11,13^. Notably, perturbing the fine-tuned scarred initial states away from the semiclassical time-dependent variational principle (TDVP) trajectory destroys the periodic revivals. Even a single defect inserted into the $\left| {{\mathbb{Z}}}_{2}\right\rangle$ state, $\left| \ldots \downarrow \uparrow \downarrow \downarrow \downarrow \uparrow \downarrow \ldots \right\rangle$, disturbs the state within a light-cone that ballistically expands throughout the chain in both directions^25,26^. While this impurity can be confined by a strong staggered potential^25,27^ or reflected by a large potential barrier to achieve propagation in a single direction^28^, stabilizing it seemingly always requires a strong deformation of the PXP Hamiltonian.

To obtain coherently propagating chiral excitations in the PXP model, we focus on a different scarred 3-site periodic initial state that was obtained in earlier works using semiclassical trajectories^24^ and an approximate su(2) algebra^29^ (which explains previously reported revivals of the product state $\left| {{\mathbb{Z}}}_{3}\right\rangle=\left| \downarrow \downarrow \uparrow \downarrow \downarrow \uparrow \ldots \right\rangle$^19^ through the large overlap between the two states). This state, with *N* atoms and *N*/3 unit cells, can be built by repeating the following weakly-entangled 3-site unit cell:

3 $$\left| K\right\rangle=\mathop{\bigotimes }_{k=1}^{N/3}\left| S\right\rangle,\qquad \left| S\right\rangle=\frac{1}{\tilde{\beta }}\left| \downarrow \right\rangle (\beta \left| \uparrow \downarrow \right\rangle+\left| \downarrow \uparrow \right\rangle ),$$

where $$\tilde{\beta }=\sqrt{{\beta }^{2}+1}$$ and *β* = 0.65.

### Soliton excitations

Coherently propagating defects—which we will refer to as solitons—can be constructed on top of the 3-site periodic initial state $\left| K\right\rangle$. The local Hilbert space of allowed 3-site cells that obey the constraint, when surrounded by $\left| S\right\rangle$ cells, has dimension 3, and can be spanned by the scarred cell $\left| S\right\rangle$, along with the left- and right-moving defect cells $\left| L\right\rangle$ and $\left| R\right\rangle$. These 3-site defect cells can be determined by requiring (i) the first site to be in the ground state to avoid blockade violation, (ii) $\{\left| S\right\rangle,\left| L\right\rangle,\left| R\right\rangle \}$ being orthonormal to each other and (iii) to satisfy the conjugation condition under chiral symmetry, $\left| L\right\rangle={{{\mathcal{C}}}}\left| R\right\rangle$.

**Fig. 2:**
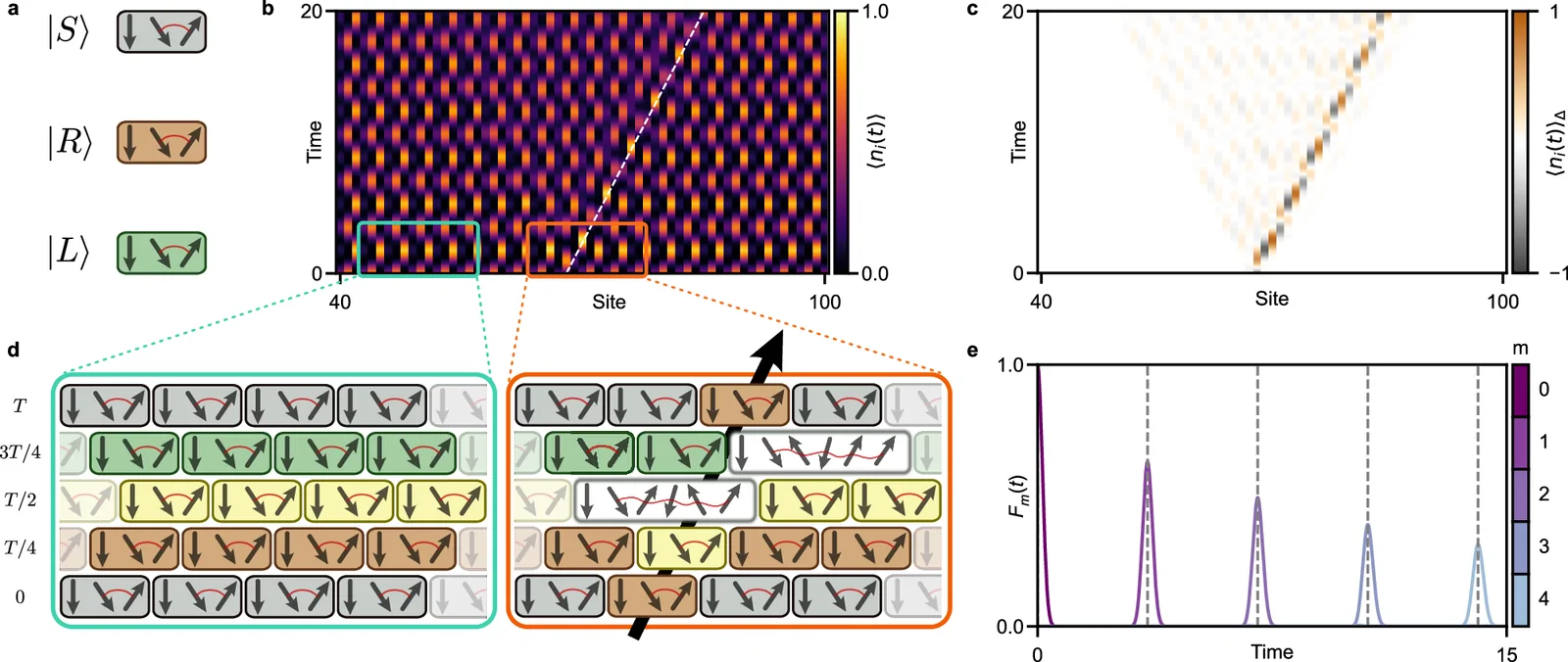
Dynamics of a single right-moving soliton initialized in the scarred state $\left| K\right\rangle$ at sites 67 − 69 in a 150-site Rydberg atom chain. **a** Legend for scarred cell $\left| S\right\rangle$ and soliton cells $\left| R\right\rangle$ and $\left| L\right\rangle$. The first site in each cell is unexcited, while the second and third sites form an entangled pair. This entangled pair differs for each cell, as indicated by the different colors of the cells, and its exact form is listed in Eqs. (3) and (4). **b** The soliton appears as a mobile defect that locally disrupts the homogeneous precession of the number operator; its trajectory is highlighted by the dashed white line. **c** The difference in the number-operator expectation values between the states with and without the soliton indicates a density perturbation that predominantly moves to the right. **d** (Left) Schematic dynamics of scarred unit cells, $\left| S\right\rangle$, shown by gray color within the revival period *T*. (Right) The phase-shifted unit cell from the quarter-periods of the scarred dynamics (orange color) corresponding to $\left| R\right\rangle$ in Eq. (4) is embedded within the $\left| S\right\rangle$ background. It coherently moves one cell to the right over each revival period *T*, corresponding to a right-moving soliton. Green unit cells at 3*T*/4 correspond to left-moving soliton $\left| L\right\rangle$, and the state of remaining yellow and white cells is discussed in Supplementary Note 1^30^. **e** Peaks in the translation fidelity (7) for the subsystem *A* = {61, 62, …, 90} at multiples of *T* indicate near-coherent rightward translation of the entire wave function. Details of numerical simulation and fidelity extraction are available in the Methods section.

Among the one-parameter family of solutions satisfying the above conditions, we find that the following leads to the most pronounced coherent directional propagation, see Fig. 2a:

4 $$\begin{array}{rcl}\left| L,R\right\rangle &=&\frac{1}{\sqrt{2}\tilde{\beta }}\left| \downarrow \right\rangle (\mp \tilde{\beta }\left| \downarrow \downarrow \right\rangle+i\left| \uparrow \downarrow \right\rangle -i\beta \left| \downarrow \uparrow \right\rangle ),\end{array}$$

with $\beta,\tilde{\beta }$ being the numerical constants defined after Eq. (3). Below, we will consider the dynamics of initial states formed by $\left| S\right\rangle$ unit cells with the insertion of $\left| L,R\right\rangle$ defects. For brevity, we denote such states as $\left| {K}^{{{{\bf{l}}}},{{{\bf{r}}}}}\right\rangle$, where the disjoint sets **l** and **r** specify locations of cells with $\left| L\right\rangle$ and $\left| R\right\rangle$ states,

5 $$\left| {K}^{{{{\bf{l}}}},{{{\bf{r}}}}}\right\rangle=\mathop{\bigotimes }_{j=1}^{N/3}\left\{\begin{array}{ll}{\left| L\right\rangle }_{j}\quad &\,{{\mbox{for}}}\,j\in {{{\bf{l}}}},\hfill \\ {\left| R\right\rangle }_{j}\quad &\,{{\mbox{for}}}\,j\in {{{\bf{r}}}},\hfill \\ {\left| S\right\rangle }_{j} \quad &\,{{\mbox{otherwise}}}\,.\end{array}\right.$$

A single unit cell $\left| R\right\rangle$ initialized in the background of the scarred initial state $\left| K\right\rangle$ shows coherent propagation to the right under the unitary evolution with the PXP Hamiltonian, as is visualized in Fig. 2b. This propagation is visible in the expectation value of the Rydberg atom number operator, defined as $n=\left| \uparrow \right\rangle \left\langle \uparrow \right|$, shown for the time evolved state $\left| {K}^{\varnothing,\{j\}}(t)\right\rangle={e}^{-iHt}\left| S\ldots SS{R}_{j}SS\ldots S\right\rangle$ with a single $\left| R\right\rangle$ state inserted near the middle. Far away from the initial location of the $\left| R\right\rangle$ cell, the dynamics follow periodic revivals. Therefore, in Fig. 2c we show the difference between the expectation value of the number operator for the time-evolved states with and without the right-moving soliton,

6 $${\langle {n}_{i}(t)\rangle }_{\Delta }=\langle {K}^{{{{\bf{l}}}},{{{\bf{r}}}}}(t)| {n}_{i}| {K}^{{{{\bf{l}}}},{{{\bf{r}}}}}(t)\rangle -\langle K(t)| {n}_{i}| K(t)\rangle .$$

Figure 2c confirms that the $\left| R\right\rangle$ defect gives a right-moving soliton, as its presence does not lead to the typical symmetric light-cone spreading both to the left and right, but instead affects the state of the system to the right.

The phenomenological explanation of the mechanism by which the $\left| R\right\rangle$ impurity forms a right-moving soliton is displayed in Fig. 2d. There, we first sketch the dynamics of the scarred initial state $\left| K(t)\right\rangle$ over one period *T* ≈ 3.52. Notably, the squared absolute value of the overlap of the state $\left| R\right\rangle$ identified in Eq. (4) with the unit cells of the $\left| K(T/4)\right\rangle$ state is 0.98 (the same holds for the overlap of $\left| L\right\rangle$ and the cell at 3*T*/4). Therefore, the creation of the right-moving soliton corresponds to a phase shift of a particular unit cell in the $\left| K\right\rangle$ initial state by a quarter period, as shown in the right panel of Fig. 2d. This fast-forwarded unit cell naturally gets shifted to the right after time *T*/4. After this, the state of the soliton can no longer be viewed as a 3-site cell, as it forms an entangled state with the adjacent cell to the left. See Supplementary Fig. 1^30^ for additional details. By the full period, the soliton cell $\left| R\right\rangle$ reemerges, but now shifted by 3 sites to the right. A similar picture holds for the insertion of the $\left| L\right\rangle$ cell into the $\left| K\right\rangle$ state, which, however, propagates to the left, corresponding to the left-moving soliton. The left propagation of $\left| L\right\rangle$ under unitary dynamics follows from the chiral symmetry of the Hamiltonian (2) and the relation $\left| L\right\rangle={{{\mathcal{C}}}}\left| R\right\rangle$ (see Supplementary Note 1^30^).

To quantify how coherently the solitons move between different unit cells under unitary dynamics, we define a family of measures called translation fidelities, labeled by index *m* specifying the number of unit cells each right-/left-moving soliton has shifted towards the right/left:

7 $${F}_{m}(t)={\left| \left\langle {K}^{{{{\bf{l}}}}-m,{{{\bf{r}}}}+m}\left| {K}^{{{{\bf{l}}}},{{{\bf{r}}}}}(t)\right.\right\rangle \right| }^{2},$$

with **r** + *m*: = {*j* + *m*∣*j* ∈ **r**}. As shown in Fig. 2e, the translation fidelities for the initial state with a single right-moving soliton show large peaks occurring at *t* = *mT* for *F*_*m*_(*t*). As a result of the slowly scrambling scarred background and the slow decay of the solitons, the fidelities *F*_*m*_(*t*) decrease with increasing *m*. Nevertheless, for *m* = 4 at *t* ≈ 14 in a large 30-site system, the fidelity remains at 30%, indicating coherent transport of $\left| R\right\rangle$ over 12 sites. Even as fidelity diminishes locally, the soliton still shows coherent propagation, as seen in Fig. 2c. This is in stark contrast to the rapid thermalization of generic states (see Supplementary Fig. 2^30^). Long-lived coherence and the preservation of the initial information were previously known for scarred dynamics. However, the soliton state is spatially inhomogeneous, orthogonal to the scarred state $\left| K\right\rangle$, and undergoes spatial translation, therefore representing a distinct class of non-ergodic behavior in highly excited states.

### Soliton stability and collisions

**Fig. 3:**
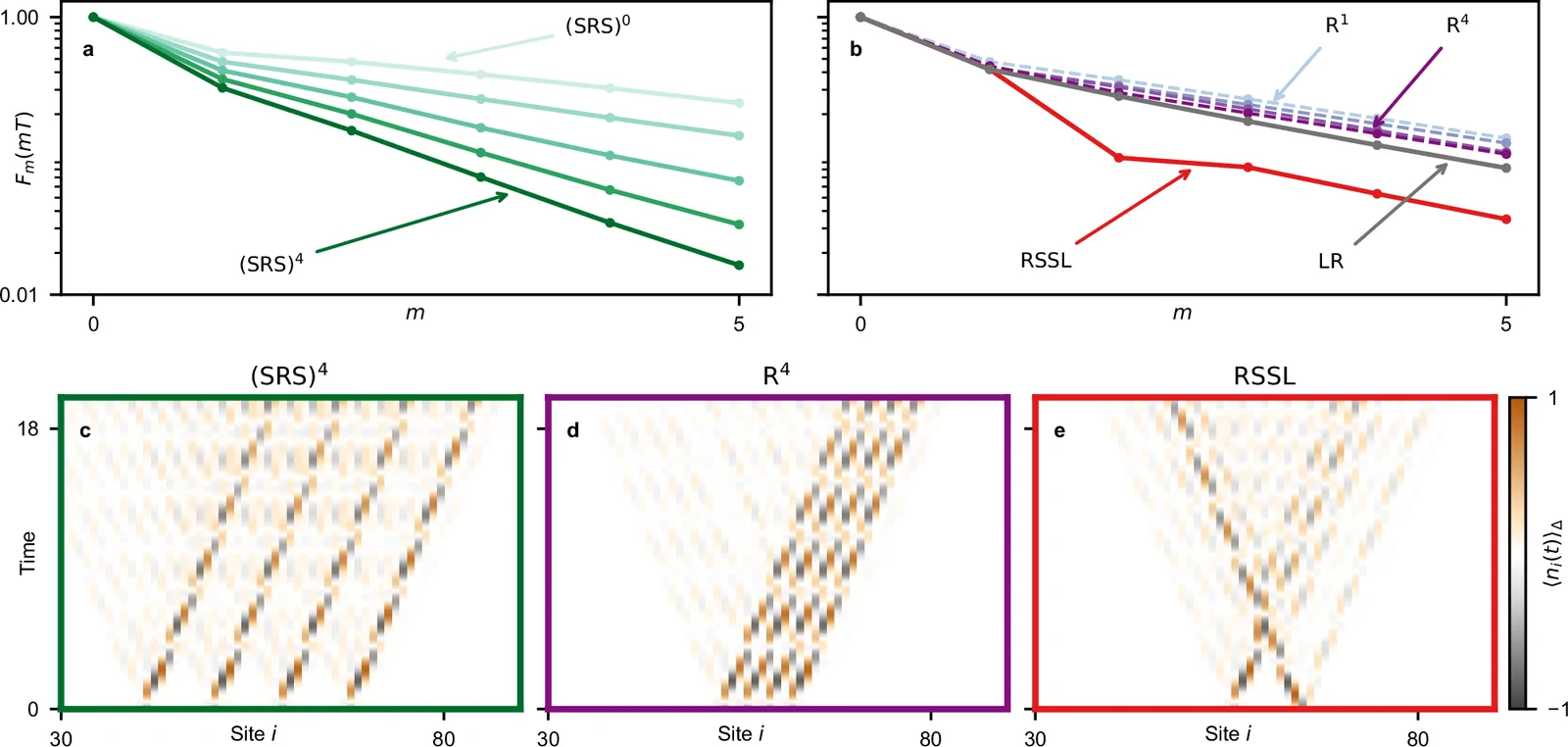
Decay of initial multi-soliton states under unitary dynamics. **a** For isolated right-moving solitons, the decay rate is proportional to the number of solitons. **b** In contrast, for initial states where right-moving solitons are placed side-by-side, the decay rate is nearly independent of the number of solitons, implying that domain walls between $\left| S\right\rangle$ and soliton cells are responsible for the enhanced decay. **c**, **d** The expectation value of $\langle n\rangle_{\Delta}$, Eq. (6), also confirms that the initial four-soliton state with isolated solitons features less coherent propagation compared to the dynamics of the four-soliton bulk excitation. **e** The collision of right-/left-moving solitons in the time interval *t* ∈ [*T*, 2*T*] leads to a rapid decrease in the translation fidelity visible in **b**. Fidelities are shown for the subsystem *A* = {31, 32, …, 90}, see Methods for details.

After establishing individual right- and left-moving solitons, we study the dynamics of initial states with multiple solitons in Fig. 3. To this end, we first consider states with *k* right-moving solitons $\left| R\right\rangle$, each consecutive pair of solitons separated by two cells $\left| S\right\rangle$, embedded in our scarred state $\left| K\right\rangle$ in the form of $\left| \ldots SRS\ldots SRS\ldots \right\rangle$. Figure 3a shows the value of the peaks in the translation fidelities (7) at the corresponding multiple of the reviving period *T*. The data labeled with *k* = 0 corresponds to the dynamics of the scarred initial state $\left| K(t)\right\rangle$, whose revivals feature weak but nevertheless exponential decay with time. Introducing isolated solitons into the initial state $\left| K\right\rangle$ enhances this decay with time, by an amount approximately proportional to the number of solitons. Note that, although the smallest peak in fidelity revivals is only about 1% for the four-soliton state, this is still a very large fidelity for a region of 60 sites. Accordingly, the propagation of solitons remains clearly visible in the expectation value of the number operator, see Fig. 3c.

In contrast to the isolated multi-soliton state, for the initial state where right-moving solitons are adjacent to each other in the form of $\left| RR\right\rangle$, $\left| RRR\right\rangle$ and $\left| RRRR\right\rangle$, the fidelity suppression is almost independent of the number of solitons, see Fig. 3b. The coherent propagation of solitons is also clearer in the number operator dynamics in Fig. 3d. The contrasting behavior in fidelity of multi-soliton states in Fig. 3a-b suggests that additional decoherence originates not from the solitons, but from the domain walls separating the $\left| R,L\right\rangle$ soliton and scar cells $\left| S\right\rangle$. This is consistent with the identification of soliton cells with phase-shifted unit cells of the scarred initial state: phase-shifting the entire scarred state by *T*/4 should give no additional decay, which is equivalent to the $\left| RR\ldots \right\rangle$ state.

In Fig. 3b, we also show the additional decay from the collision between solitons propagating in opposite directions. When two counter-propagating solitons are created in a non-colliding configuration, $\left| \ldots LR\ldots \right\rangle$, translation fidelities show smooth exponential decay with time. In contrast, the colliding configuration $\left| \ldots RSSL\ldots \right\rangle$ displays a sudden drop in fidelity (see Fig. 3b), which is also visible in the dynamics of the number operator in Fig. 3e.

Finally, we quantify the lifetime of a single soliton. After the initial sharp drop, the translation fidelities in Fig. 3a show a consistent exponential decay for *t*≥*T*. Therefore, we extract the exponential decay rate between the stroboscopic peaks *T* and 5*T*. To isolate the local quasi-soliton decay from the extensive decay of the scarred background, we calculate the translation fidelity ratio $R(m)={F}_{m}^{(1)}(mT)/{F}_{m}^{(0)}(mT)$, where ${F}_{m}^{(1)}$ corresponds to the single quasi-soliton state (*SRS*)^1^ and ${F}_{m}^{(0)}$ corresponds to the scarred state (*SRS*)^0^. This quantity is insensitive to system size and captures the local soliton-induced decay. Assuming $R(m)\propto {e}^{-{\gamma }_{{{{\rm{sol}}}}}mT}$ in this window, we estimate the decay rate as ${\gamma }_{{{{\rm{sol}}}}}=-\ln [R(5)/R(1)]/(4T)$ and define the soliton lifetime as *τ*_sol_ = 1/*γ*_sol_. Using the values of our numerical simulations shown in Fig. 3a, we get a lifetime of *τ*_sol_ ≈ 36.

### Energy transport via solitons

The discussed left- and right-moving solitons $\left| L,R\right\rangle$ have—as the scarred initial state—zero energy density, 〈*K*^**l,r**^∣*P*_*i*−1_*X*_*i*_*P*_*i*+1_∣*K*^**l,r**^〉 = 0 for any *i* and **l**, **r** (see Supplementary Note 2^30^). Next, we generalize $\left| L,R\right\rangle$ solitons into a continuous family of energy-carrying solitons $\left| {L}^{\alpha },{R}^{\alpha }\right\rangle$, by forming a superposition of the original $\left| L,R\right\rangle$ cell and the scar unit cell $\left| S\right\rangle$ labeled by an angle *α*,

8 $$\left| {R}^{\alpha }\right\rangle=\cos \alpha \left| R\right\rangle+\sin \alpha \left| S\right\rangle,\quad \alpha \in [-\frac{\pi }{2},\frac{\pi }{2}).$$

**Fig. 4:**
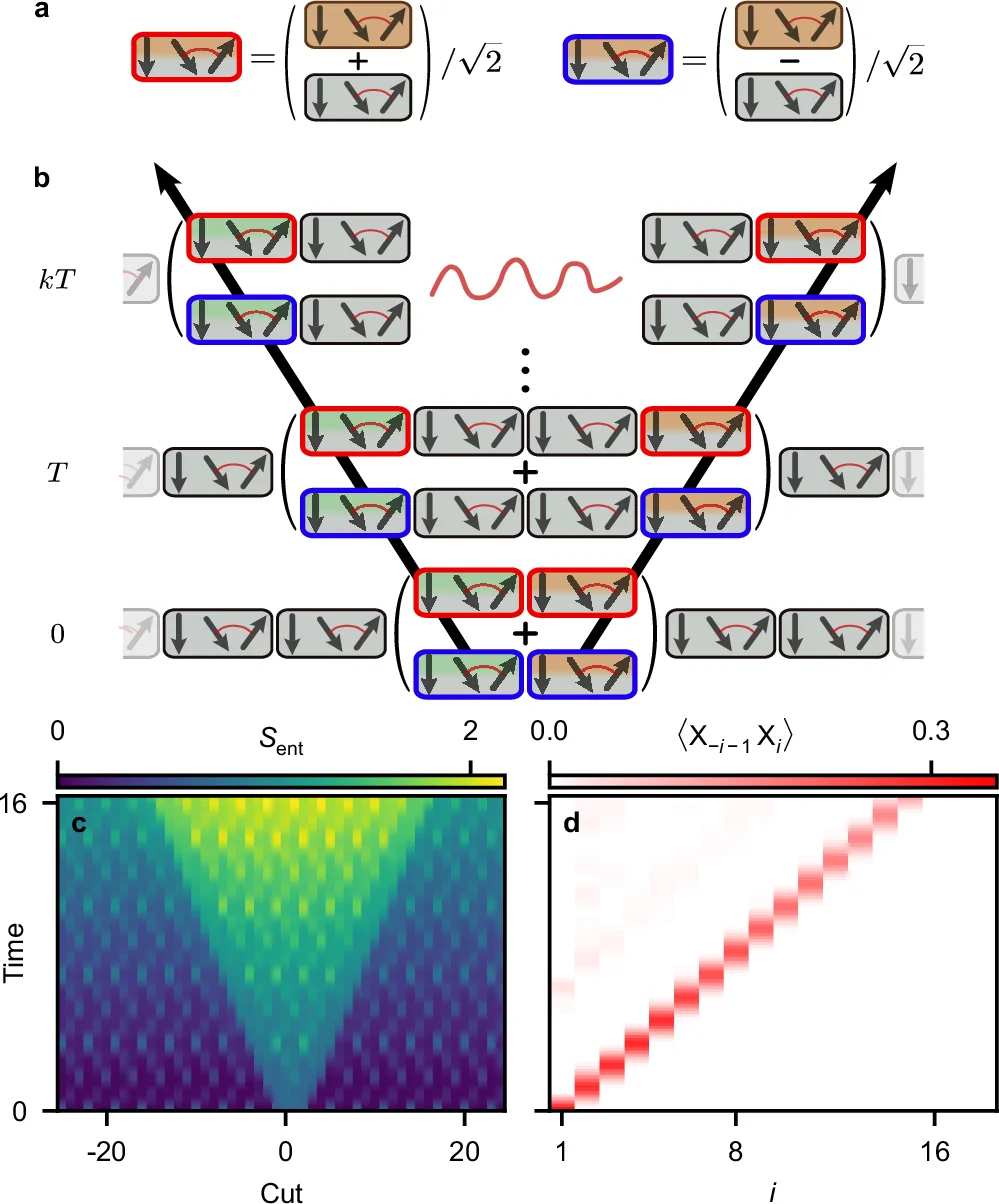
Time evolution of an entangled Bell pair of left- and right-moving solitons embedded in the $\left| K\right\rangle$ state at sites {49, …54} in a chain with 102 sites. **a** Right-moving energy-soliton construction of $\left| {R}^{+}\right\rangle$ (red) and $\left| {R}^{-}\right\rangle$ (blue). Left-moving cells $\left| {L}^{\pm }\right\rangle$ are constructed identically by replacing the brown $\left| R\right\rangle$ cells by the green $\left| L\right\rangle$ cells. **b** Schematic illustration of the dynamics at integer multiples of the revival period *T*. **c** Bipartite entanglement entropy *S*_ent_ for different cuts across the chain (with zero corresponding to the link between the original Bell pair cells) shows the spreading of an entanglement cone as the solitons move apart. **d** The connected energy density correlation function, ${\langle {X}_{-i-1}{X}_{i}\rangle }_{c}$, exhibits positive correlations along a trajectory with a slope matching the inverse soliton velocity, thus providing an experimental way of confirming long-range quantum information transfer via solitons.

Such unit cells carry a total energy of ${\epsilon }_{\alpha }=\sin \left(2\alpha \right)(1+\beta )/\sqrt{2(1+{\beta }^{2})}$ (see Supplementary Note 2^30^ for details). The left-moving state $\left| {L}^{\alpha }\right\rangle$ is defined analogously, and carries energy  − *ϵ*_*α*_ due to chiral symmetry. In what follows, we focus on the dynamics of solitons with the maximal (minimal) energy value, denoted as $\left| {R}^{\pm }\right\rangle=\left| {R}^{\pm \pi /4}\right\rangle$ (see Fig. 4a) and $\left| {L}^{\pm }\right\rangle=\left| {L}^{\mp \pi /4}\right\rangle$. Since the energy soliton cell $\left| {R}^{\pm }\right\rangle$ is a linear superposition of $\left| S\right\rangle$ and $\left| R\right\rangle$ states, we expect that $\left| {R}^{\pm }\right\rangle$ propagates identically to $\left| R\right\rangle$.

The coherent propagation of energy-carrying solitons is shown in Fig. 1 via the expectation values of the number operator density **a** and the local energy density **b**, denoted as $\langle {X}_{i}(t)\rangle=\left\langle \psi (t)\right| {P}_{i-1}{X}_{i}{P}_{i+1}\left| \psi (t)\right\rangle$. The solitons carry energy over long distances with a weak residual decay. The collision between $\left| {R}^{+}\right\rangle$ and $\left| {L}^{+}\right\rangle$ leads to an additional suppression in their energy density, but no detectable phase shift occurs^6^. In Supplementary Note 2^30^, we present more detailed data with respect to the slow decay of multi-soliton states, and furthermore, we show how solitons can be used to construct different homogeneous scarred initial states with tunable energy density.

### Information transmission

The coherent propagation of solitons with an internal degree of freedom that corresponds to the energy offers a way to transport information through the Rydberg chain and access it at times that are multiples of the revival period *T*. To this end, we use the orthogonal states $\left| {R}^{\pm }\right\rangle$ and $\left| {L}^{\pm }\right\rangle$ to create a Bell pair of left and right-moving solitons, $(\left| {L}^{+}{R}^{+}\right\rangle+\left| {L}^{-}{R}^{-}\right\rangle )/\sqrt{2}$ embedded in $\left| K\right\rangle$, see Fig. 4b, traveling in opposite directions without collision. Figure 4c shows the evolution of the bipartite entanglement entropy ${S}_{{{{\rm{ent}}}}}=-{{{\rm{Tr}}}}\left[{\rho }_{A}\ln {\rho }_{A}\right]$ for different cuts through the chain. The clearly visible light cone spreads in Fig. 4c from the cut position at 0, chosen to be in-between the original soliton Bell pair. To highlight the feasibility of experimental observations, Fig. 4d shows the dependence of the connected energy density correlation function between sites that are mirror-symmetric with respect to the cut position at 1, ${\langle {X}_{-i-1}{X}_{i}\rangle }_{c}=\langle {X}_{-i-1}{X}_{i}\rangle -\langle {X}_{-i-1}\rangle \langle {X}_{i}\rangle$. Since the soliton cells each move with a constant velocity *v*_sol_, the maximum of the correlation function *X*_−*i*−1_*X*_*i*_ occurs at stroboscopic times *t* = *k*/*v*_sol_ at *i* = 1 + *k* with $k\in {\mathbb{N}}$, forming a straight line in Fig. 4d. This soliton-based quantum information transmission procedure is readily implementable in current-generation Rydberg atom setups without requiring site-dependent couplings as in other proposed methods^31^. We note that our demonstrated information transmission is meant only as a proof of principle. Our key point is that quantum information can be transported coherently by quasi-solitonic excitations even though the system is chaotic. By contrast, ref. ^32^ develops a model for universal quantum computation in Rydberg arrays using global driving. In their model, quantum information is also transported along the chain, but via a pulse-cycle protocol rather than continuous time evolution under a time-independent Hamiltonian.

### Variational description

After demonstrating the existence of solitonic excitations in exact unitary dynamics, we turn to their variational description using an MPS manifold (see Eq. (11)), which intuitively corresponds to the projection of a spin-1/2 coherent state onto the constrained subspace of the PXP model. Projection of exact unitary dynamics onto such a variational manifold with TDVP results in a first-order system of nonlinear differential equations with a chain of *N* sites leading to 2*N* classical angles *θ*_*i*_, *ϕ*_*i*_, describing the *i*-th spin rotation on the Bloch sphere.

Notably, such an approach was earlier considered only with small unit cells (up to four sites^23,24,29^). We generalize the TDVP equations of motion to an infinite chain with a generic unit cell of *N* sites (see Supplementary Note 4^30^; see also an independent recent work^33^). When constrained to the subspace with zero energy expectation value, *ϕ*_*i*_ = *π*/2, our equations of motion take a simple form

9 $${\partial }_{t}{\theta }_{1}=-\cos {\theta }_{2}-{\sin }^{3}{\theta }_{1}\cot {\theta }_{N}\frac{\langle {n}_{N}\rangle }{\langle {n}_{1}\rangle },$$

where 〈*n*_*i*_〉 is the expectation value of the number operator on site *i* of the unit cell, see Eq. (15) in Methods. The value of ∂_*t*_*θ*_*j*_ for the remaining angles can be obtained by applying cyclic permutations, 1 → 2 → 3…. Equations (9) are nonlinear and non-local, since the evolution of a given angle explicitly depends on all other angles via the expectation values of the number operators.

**Fig. 5:**
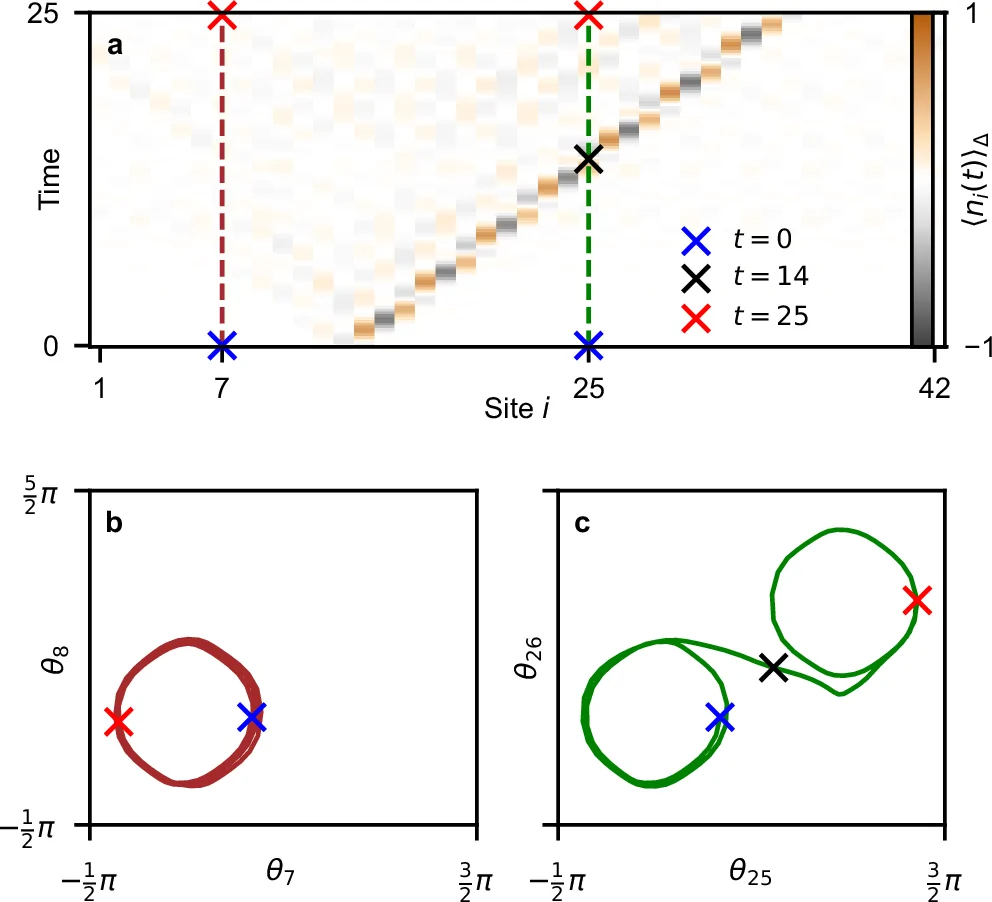
Time evolution of classical equations of motion (9) of an infinite chain with a 42-site unit cell up to time *t* = 25. The initial state has all sites initialized with ${\theta }_{1,2,3}^{S}$ angles except for the sites {13, 14, 15} initialized with right-moving soliton ${\theta }_{1,2,3}^{R}$ angles. **a** The difference in the expectation value of the number operator between states with and without a soliton shows the predominant propagation of the disturbance to the right. **b**, **c** Dynamics of *θ*_7,8_ and *θ*_25,26_ are constrained to approximately closed trajectories. The propagation of the soliton through sites 25 and 26 moves the dynamics of the corresponding angles to a different, almost closed orbit.

This variational description in terms of angles *θ*_*i*_ captures the homogeneous scarred state $\left| K\right\rangle$^24^ and the states $\left| {K}^{{{{\bf{l}}}},{{{\bf{r}}}}}\right\rangle$. At the same time, dynamics generated by Eq. (9) from the initial state with zero-energy solitons $\left| {K}^{{{{\bf{l}}}},{{{\bf{r}}}}}\right\rangle$ lead to their rapid decay, while the energy solitons show better stability (see Supplementary Fig. 9^30^). The discrepancy between TDVP and exact unitary dynamics is not surprising, as the projection makes TDVP equations nonlinear and may lead to disagreement with unitary evolution. Therefore, we search for solitons directly in the TDVP equations of motion. Numerical optimization of a quantity similar to translation fidelity by three sites (see Supplementary Note 4^30^) results in angles ${\theta }_{1,2,3}^{S}$ and ${\theta }_{1,2,3}^{R}$ listed in “Methods”. The homogeneous state described by the 3-site unit cell with angles ${\theta }_{1,2,3}^{S}$ is very close to a point on the trajectory of $\left| K\right\rangle$. The defect cell deviates more strongly from our previously reported right-moving soliton; however, Fig. 5a reveals a dynamics of ${\langle {n}_{i}(t)\rangle }_{\Delta }$ that is very similar to the one observed in exact unitary dynamics (see Supplementary Note 4^30^ for more direct comparison). Moreover, when tracking dynamics of individual classical variables *θ*_*i*_, shown in Fig. 5b,c, we observe that they display periodic motion along approximately closed trajectories. The passing of a soliton through a given site disturbs this motion briefly, after which the angle returns to a replica of the same trajectory, see Fig. 5c. Note that the simple behavior of angles that are not physical observables translates into more complex behavior of local expectation values, such as 〈*n*_*i*_〉. Finally, we note that the capability of TDVP to describe the dynamics faithfully depends on the leakage of the trajectory out of the given variational manifold. While we do not explicitly compute the leakage, previous work has shown that the leakage of the discussed scarred trajectory (without a soliton) is small^24^, suggesting that TDVP can describe its dynamics. Moreover, in Supplementary Fig. 4^30^ we show that the entanglement growth of the purely scarred state and of the state with a single solitonic defect is comparably slow, which supports the expectation that the same variational manifold provides a good description in both cases.

## Discussion

We have demonstrated that the PXP model in one-dimensional atom chains hosts discrete quasi-solitons—localized, potentially energy-carrying, wave packets that propagate coherently over long distances. The quasi-solitons identified in our work propagate on top of special reviving initial states, and do not have a dispersion, as their velocity is fixed by the unit cell size and the revival period of the underlying state. Quasi-solitons exhibit weak decay if propagating in the same direction, and stronger decay upon collisions. Importantly, they enable directed transport of energy and quantum information, suggesting potential uses in communication protocols in synthetic quantum systems. We have also shown that similar coherent structures emerge in a classical system governed by nonlinear equations of motion derived via TDVP.

We want to highlight how unusual the coherent transport of the quasi-solitons in the background of these special reviving states is. All states considered in our work correspond to highly-excited (if not infinite temperature) states of the system, and the coherent propagation of quasi-solitons is not a manifestation of low-energy physics. Furthermore, soliton cells $\left| L,R\right\rangle$ are orthogonal to the cells of the reviving state, $\left| S\right\rangle$, and therefore cannot be understood as a weak deformation thereof. Their spectral decomposition is also much less straightforward than for scarred states, whose dynamics can be explained via the anomalously high overlap with only a few non-thermal eigenstates^13^. The initial states with embedded quasi-solitons also exhibit several outliers, but they are much less distinctly separated from the bulk, meaning that many other eigenstates contribute meaningfully to the dynamics. We note that the outliers are different than those previously identified as causing revivals from the $\left| {{\mathbb{Z}}}_{2}\right\rangle$ states in the same model, but actually match well with those responsible for the oscillatory behavior of the $\left| K\right\rangle$ state that serves as a background for the solitons (see Supplementary Fig. 7^30^).

Although we have established the existence of quasi-solitons, they lack a complete theoretical description, and many aspects remain to be investigated. Inserting left-/right-moving quasi-solitons provides the PXP model with an exponentially large manifold of non-thermal multi-soliton states, but their relevance to physical properties of the model, such as its superdiffusive energy transport^15^, remains to be understood, as well as the possible existence of other soliton-hosting states. Stabilizing quasi-solitons by deforming the PXP model^14,20,34,35^ and thereby establishing the existence of fully stable solitons, applying driving^12,22^ or potential confinement^36^ would allow us to utilize the exponentially large non-thermal Hilbert space to store and transmit information, similar to what we have shown above. Beyond theoretical descriptions, experimental studies of quantum quenches from inhomogeneous multi-soliton initial states—feasible with current Rydberg array quantum simulators^10–12^—could provide important insights. In Supplementary Fig. 14^30^, we provide additional evidence that our quasi-solitons can also be observed in experimentally realistic settings with long-range interactions and imperfect Rydberg blockade. A complementary angle to address these questions is via the study of the associated classical dynamical system (9), which hosts soliton-like excitations and may offer insights into quantum dynamics as well as connections to discrete solitons in optical and other systems^4^.

Another direction is the possible connection to solitonic quasiparticles in integrable Floquet circuits related to the PXP model, such as the RCA201 cellular automaton^35^. This Floquet circuit can be obtained by discretizing (Trotterizing) the time-continuous PXP model and choosing a large step size. In principle, there is no physical argument why a connection between solitonic quasiparticles in the time-continuous model and the cellular automaton should exist, as the Trotter step size required to obtain the cellular automaton is beyond the perturbative regime. Nevertheless, ref. ^34^ found that certain quantum many-body scars of the time-continuous PXP model, associated with the $\left| {{\mathbb{Z}}}_{2}\right\rangle$ state, can be adiabatically connected to cellular automaton eigenstates by treating the Trotter step size as an interpolation parameter. Moreover, certain dynamical signatures of cellular automaton soliton product states persist down to small Trotterization step sizes. This raises the question of whether features of our quasi-solitons in the time-continuous chaotic PXP model are related to the dynamical features of simpler, analytically tractable Floquet realizations, such as the cellular automaton. However, our quasi-soliton states are weakly entangled, so a meaningful connection would require deforming not only the step size but also the state itself into a cellular automaton product state, and it is not obvious how to do so. A more detailed discussion and initial explorations are provided in Supplementary Note 5^30^, leading to the conclusion that a connection is not apparent.

More generally, it would be interesting to understand if other scarred discrete lattice models can host solitonic excitations. In Supplementary Fig. 15^30^, we show that the PPXPP model^29^ exhibits excitations that are similar but less stable. Other potential candidates include the spin-1 XY model, where scars^37^ and unconventional energy transport were also reported^38^, as well as kinetically constrained models such as higher-spin generalizations of PXP^23^ or lattice gauge theories^39–41^. Even more intriguing are questions of potential generalization of present solitons to two-dimensional lattices and the relation to solitons in Rydberg atomic gases^42,43^, potentially coupled to light^44^ in a regime of electromagnetically induced transparency^45^. Making progress in some of the above questions will lead to a better understanding of dynamics in excited quantum systems and realizations of soliton physics in quantum simulators.

## Methods

### Time-evolving block decimation algorithm

Unitary time-evolutions in this article have been conducted via the time-evolving block decimation (TEBD) algorithm^46,47^ using the ITensor library^48,49^. The dynamics generated by Eq. (1) are obtained by using a symmetric fourth-order integrator^50,51^ with the time step chosen to be Δ*t* = 0.02. All simulations have been performed with open boundary conditions and a maximal bond dimension *χ* = 256. Singular values below the threshold 10^−12^ were discarded.

All local observables were calculated at least 30 sites away from the boundaries of the chain to eliminate potential boundary effects for the considered time windows. The translation fidelity defined in Eq. (7) is a global quantity and consequently affected by the boundaries. Therefore, we only consider a finite region of interest *A* by tracing out its complement $\bar{A}$ and calculating the translation fidelity as:

10 $${F}_{m}(t)=\langle {K}^{{{{\bf{l}}}},{{{\bf{r}}}}}(t)|{{{\rm{tr}}}}_{\bar{A}}(| {K}^{{{{\bf{l}}}}-m,{{{\bf{r}}}}+m}\rangle \langle {K}^{{{{\bf{l}}}}-m,{{{\bf{r}}}}+m}| )| {K}^{{{{\bf{l}}}},{{{\bf{r}}}}}(t)\rangle .$$

Here, the reduced operator obtained after tracing out $\bar{A}$ is understood to be embedded back into the full Hilbert space by tensoring with the identity on $\bar{A}$. If the region *A* is chosen to be the entire chain, this expression becomes equivalent to Eq. (7).

To ensure that the data produced via TEBD is reasonably faithful up to the reported times, we repeated the simulations with bond dimension *χ* = 128 and compared the results. For quantities *O* bounded by 1.0 in their reported units (number operator, energy density operator and energy-energy correlation operator expectation value, as well as translation fidelity), we have ensured that their estimates produced with different bond dimensions, denoted as *O*_*χ*_, agree. Specifically, we check that ∣*O*_256_ − *O*_128_∣≤0.01 for all times. Similarly, for the bipartite entanglement entropy, we required that the relative error is upper bounded by ∣*S*_256_ − *S*_128_∣/*S*_256_≤0.01 for all cuts at all times.

Finally, we provide the explicit form of the initial states of the TEBD simulations shown in Figs. 1– 4: Figure 1 : The initial state used in the simulation is defined on 150 sites and is given by ${\left| S\right\rangle }^{\otimes 17}\,\left| {R}^{+}\right\rangle \,{\left| S\right\rangle }^{\otimes 4}\,\left| {L}^{+}\right\rangle \,{\left| S\right\rangle }^{\otimes 3}\,{\left| {R}^{+}\right\rangle }^{\otimes 2}\,\left| {R}^{-}\right\rangle \,{\left| S\right\rangle }^{\otimes 2}\,\left| {R}^{+}\right\rangle \,{\left| S\right\rangle }^{\otimes 18}$ where the different 3-site cells are defined in the main text. Figure 2: The state $\left| {K}^{{{{\bf{l}}}},{{{\bf{r}}}}}\right\rangle$ is defined on 150 sites with ${{{\bf{l}}}}=\varnothing$, **r** = {23}.

Figure 3 : All $\left| {K}^{{{{\bf{l}}}},{{{\bf{r}}}}}\right\rangle$ states are defined on 120 sites. The states denoted as (*SRS*)^*k*^ for *k* = 0…4 correspond to ${{{\bf{l}}}}=\varnothing$ and **r** = {11 + 3*i*∣1≤*i*≤*k*}. The states denoted as *R*^*k*^ correspond to ${{{\bf{l}}}}=\varnothing,{{{\bf{r}}}}=\{17+i| 1\le i\le k\}$ for *k* = 1…4. The state *RSSL* is given by **l** = {22}, **r** = {19} and *LR* is given by **l** = {20}, **r** = {21}.

Figure 4 : The initial state is defined on 102 sites and is given by ${\left| S\right\rangle }^{\otimes 16}\,(\left| {L}^{+}{R}^{+}\right\rangle+\left| {L}^{-}{R}^{-}\right\rangle )\,{\left| S\right\rangle }^{\otimes 16}/\sqrt{2}$.

### Time-dependent variational principle

A low-dimensional manifold able to capture the scarring from the $\left| {{\mathbb{Z}}}_{2}\right\rangle$ and $\left| {{\mathbb{Z}}}_{3}\right\rangle$ states has previously been proposed for the PXP model^23,24^. We are using the same *χ* = 2 matrix-product state (MPS) Ansatz with a different parametrization of the on-site matrices:

11 $${A}_{j}^{\downarrow }=\left(\begin{array}{rc}\cos {\theta }_{j}&0\\ 1&0\end{array}\right),\qquad {A}_{j}^{\uparrow }=\left(\begin{array}{rc}0&{e}^{i{\phi }_{j}}\sin {\theta }_{j}\\ 0&0\end{array}\right),$$

and, therefore, spans the same manifold of states. The states of this manifold are defined on an infinite chain with a repeating unit cell of size *N* and take the form:

12 $$	\left| \psi (\{{\theta }_{j},{\phi }_{j}\})\right\rangle={\mathop{\lim }_{\ell \to \infty }}\,\sum_{{\sigma }_{1},\ldots,{\sigma }_{\ell }} \\ 	 {{{\rm{Tr}}}}\left(\mathop{\prod }_{i=1}^{\ell }{A}_{i}^{{\sigma }_{i}}({\theta }_{((i-1){{{\rm{mod}}}}\,N)+1},{\phi }_{((i-1){{{\rm{mod}}}}\,N)+1})\right)\left| {\sigma }_{1}\cdots {\sigma }_{\ell }\right\rangle,$$

where *σ* ∈ {*↑*, *↓*} labels the on-site basis.

Importantly, this Ansatz is able to represent the scarred state $\left| K\right\rangle$ and any combination of soliton defects $\left| L,R\right\rangle$ embedded in the state (including energy-carrying solitons $\left| {L}^{\pm },{R}^{\pm }\right\rangle$). The projection of the unitary dynamics generated by the PXP Hamiltonian onto this variational manifold yields a system of coupled nonlinear first-order differential equations. Following the scheme in ref. ^24^, equations of motion (EOMs) for the *θ* and *ϕ* angles can be obtained from ${\sum }_{j}2{{{\rm{Im}}}}\langle {\partial }_{{\theta }_{j}}\psi | {\partial }_{{\phi }_{k}}\psi \rangle {\partial }_{t}{\theta }_{j}={\partial }_{{\phi }_{k}}\left\langle \psi \right| H\left| \psi \right\rangle$ and ${\sum }_{k}2\,{{{\rm{Im}}}}\,\left\langle {\partial }_{{\theta }_{j}}\psi \,| \,{\partial }_{{\phi }_{k}}\psi \right\rangle \,{\partial }_{t}{\phi }_{k}=-{\partial }_{{\theta }_{j}}\left\langle \psi \right| H\left| \psi \right\rangle$ for all *j*, *k* ∈ [1, *N*], where $\left| \psi \right\rangle$ is the MPS with an infinitely repeating unit cell of size *N*, therefore depending on *N* variational angles *θ* and *N* variational angles *ϕ*.

We have analytically derived the EOMs for a unit cell size of *N* = 1 to 4 and conjectured its general form to be:

13 $${\partial }_{t}{\theta }_{1}=-\cos {\theta }_{2}\sin {\phi }_{1}-{\sin }^{3}{\theta }_{1}\cot {\theta }_{N}\frac{\langle {n}_{N}\rangle }{\langle {n}_{1}\rangle }\sin {\phi }_{N},$$

14 $${\partial }_{t}{\phi }_{1}=	\left[{\sin }^{3}{\theta }_{2}\frac{\langle {n}_{1}\rangle \cot {\theta }_{1}}{\langle {n}_{2}\rangle \cot {\theta }_{2}}-2\cos {\theta }_{2}\cot 2{\theta }_{1}\right]\cos {\phi }_{1}\\ 	+\cos {\theta }_{3}\tan {\theta }_{2}\cos {\phi }_{2}+\sin {\theta }_{1}\frac{\langle {n}_{N}\rangle \cot {\theta }_{N}}{\langle {n}_{1}\rangle \cot {\theta }_{1}}\cos {\phi }_{N},$$

with the expectation value of the number operator 〈*n*_*j*_〉 on site *j* given by:

15 $$\langle {n}_{1}\rangle={\sin }^{2}{\theta }_{1}\frac{\mathop{\sum }_{l=0}^{N-1}\mathop{\prod }_{j=1}^{l}(-{\sin }^{2}{\theta }_{N-j+1})}{1-\mathop{\prod }_{j=1}^{N}(-{\sin }^{2}{\theta }_{j})}.$$

The EOMs and expectation values for other angles can be simply obtained by a cyclic permutation of the indices as *j* → *j* + 1. These equations of motion match those independently derived in ref. ^33^, and their equivalence is shown in Supplementary Note 4^30^.

The plane with *ϕ*_*j*_ = *π*/2 forms a flow-invariant subspace where ∂_*t*_*ϕ*_*j*_ = 0 ∀ *j*, as follows from Eq. (14). This submanifold is able to express all our states $\left| {K}^{{{{\bf{l}}}},{{{\bf{r}}}}}\right\rangle$ and the EOMs simplify to the ones given in Eq. (9). The number operator 〈*n*_*j*_〉 is still given by Eq. (15) as this quantity is independent of the phase information encoded in the *ϕ*_*j*_ angles. In order to search for periodic trajectories and solitons, we evolve the system of first-order differential equations (9) numerically via SciPy’s Runge-Kutta RK45 integrator of order 5(4)^52,53^. The numerically optimized angles, as described in Supplementary Note 4^30^, are given by:

$${\theta }_{1,2,3}^{S}	=(1.05977133,1.46820419,0.0000131027276),\\ {\theta }_{1,2,3}^{R}	=(0.990853599,0.454003269,3.10125177).$$

In Fig. 5, the angles ${\theta }_{1,2,3}^{S}$ were used as a background, and a right-moving soliton was launched by initializing a particular three-site unit cell with angles ${\theta }_{1,2,3}^{R}$.

## Supplementary information

Supplementary Information

Transparent Peer Review file

## Data Availability

The raw data used to generate the figures are available at ref. ^54^.

## Code availability

The TEBD algorithm used in this work was implemented using the ITensor library^48,49^ and the integration of the classical differential equations was performed via SciPy’s Runge-Kutta RK45 integrator^52,53^. The code used in this study to produce the plots from the shared data is available at ref.^54^.

## References

[1] Nguyen, J. H. V., Dyke, P., Luo, D., Malomed, B. A. & Hulet, R. G. Collisions of matter-wave solitons. *Nat. Phys.* **10**, 918–922 (2014).

[2] Grams, C. P. et al. Observation of chiral solitons in LiCuVO4. *Commun. Phys.* **5**, 37 (2022).

[3] Ustinov, A. V. et al. Dynamics of Sine-Gordon solitons in the annular Josephson junction. *Phys. Rev. Lett.* **69**, 1815–1818 (1992). 10046321 10.1103/PhysRevLett.69.1815

[4] Lederer, F. et al. Discrete solitons in optics. *Phys. Rep.* **463**, 1–126 (2008).

[5] Zabrodin, A. Classical facets of quantum integrability. Preprint at https://arxiv.org/abs/2501.18557 (2025).

[6] Vlijm, R. et al. Quasi-soliton scattering in quantum spin chains. *Phys. Rev. B* **92**, 214427 (2015).

[7] Fukuhara, T. et al. Microscopic observation of magnon bound states and their dynamics. *Nature* **502**, 76–79 (2013). 24067608 10.1038/nature12541

[8] Fendley, P., Sengupta, K. & Sachdev, S. Competing density-wave orders in a one-dimensional hard-boson model. *Phys. Rev. B* **69**, 075106 (2004).

[9] Lesanovsky, I. & Katsura, H. Interacting Fibonacci anyons in a Rydberg gas. *Phys. Rev. A* **86**, 041601(R) (2012).

[10] Browaeys, A. & Lahaye, T. Many-body physics with individually controlled Rydberg atoms. *Nat. Phys.* **16**, 132–142 (2020).

[11] Bernien, H. et al. Probing many-body dynamics on a 51-atom quantum simulator. *Nature* **551**, 579–584 (2017). 29189778 10.1038/nature24622

[12] Bluvstein, D. et al. Controlling quantum many-body dynamics in driven Rydberg atom arrays. *Science* **371**, 1355–1359 (2021). 33632894 10.1126/science.abg2530

[13] Turner, C. J., Michailidis, A. A., Abanin, D. A., Serbyn, M. & Papić, Z. Weak ergodicity breaking from quantum many-body scars. *Nat. Phys.* **14**, 745–749 (2018).

[14] Choi, S. et al. Emergent SU(2) dynamics and perfect quantum many-body scars. *Phys. Rev. Lett.* **122**, 220603 (2019). 31283292 10.1103/PhysRevLett.122.220603

[15] Ljubotina, M., Desaules, J.-Y., Serbyn, M. & Papić, Z. Superdiffusive energy transport in kinetically constrained models. *Phys. Rev. X* **13**, 011033 (2023).

[16] Serbyn, M., Abanin, D. A. & Papić, Z. Quantum many-body scars and weak breaking of ergodicity. *Nat. Phys.* **17**, 675–685 (2021).

[17] Moudgalya, S., Bernevig, B. A. & Regnault, N. Quantum many-body scars and Hilbert space fragmentation: A review of exact results. *Rep. Prog. Phys.* **85**, 086501 (2022). 10.1088/1361-6633/ac73a0 35617909

[18] Chandran, A., Iadecola, T., Khemani, V. & Moessner, R. Quantum many-body scars: A quasiparticle perspective. *Annu. Rev. Condens. Matter Phys.* **14**, 443–469 (2023).

[19] Turner, C. J., Michailidis, A. A., Abanin, D. A., Serbyn, M. & Papić, Z. Quantum scarred eigenstates in a Rydberg atom chain: entanglement, breakdown of thermalization, and stability to perturbations. *Phys. Rev. B* **98**, 155134 (2018).

[20] Khemani, V., Laumann, C. R. & Chandran, A. Signatures of integrability in the dynamics of Rydberg-blockaded chains. *Phys. Rev. B* **99**, 161101 (2019).

[21] Park, H. K. & Lee, S. Graph-theoretical proof of nonintegrability in quantum many-body systems: Application to the PXP model. *Phys. Rev. B* **111**, L081101 (2025).

[22] Maskara, N. et al. Discrete time-crystalline order enabled by quantum many-body scars: Entanglement steering via periodic driving. *Phys. Rev. Lett.* **127**, 090602 (2021). 34506175 10.1103/PhysRevLett.127.090602

[23] Ho, W. W., Choi, S., Pichler, H. & Lukin, M. D. Periodic orbits, entanglement, and quantum many-body scars in constrained models: Matrix product state approach. *Phys. Rev. Lett.* **122**, 040603 (2019). 30768339 10.1103/PhysRevLett.122.040603

[24] Michailidis, A. A., Turner, C. J., Papić, Z., Abanin, D. A. & Serbyn, M. Slow quantum thermalization and many-body revivals from mixed phase space. *Phys. Rev. X* **10**, 011055 (2020).

[25] Surace, F. M. et al. Lattice gauge theories and string dynamics in Rydberg atom quantum simulators. *Phys. Rev. X* **10**, 021041 (2020).

[26] Chen, G., Huang, W. & Yao, Y. Dynamics of entangled domain walls in the PXP model under driving: Crossover from prethermalization to localization. *Phys. Rev. B* **106**, 174302 (2022).

[27] Desaules, J.-Y. et al. Ergodicity breaking under confinement in cold-atom quantum simulators. *Quantum* **8**, 1274 (2024).

[28] Su, G.-X., Osborne, J. J. & Halimeh, J. C. Cold-atom particle collider. *PRX Quantum* **5**, 040310 (2024).

[29] Kerschbaumer, A., Ljubotina, M., Serbyn, M. & Desaules, J.-Y. Quantum many-body scars beyond the PXP model in Rydberg simulators. *Phys. Rev. Lett.* **134**, 160401 (2025). 40344113 10.1103/PhysRevLett.134.160401

[30] Supplementary Online Material (SOM). The SOM contains additional data and analyses that support the results in the main text. Refs. [12, 13, 16-18, 24, 29, 33-35, 52, 55, 56] are used in the SOM.

[31] Dooley, S., Johnston, L., Gormley, P. & Campbell, B. Perfect quantum state transfer through a chaotic spin chain via many-body scars. *Phys. Rev. B* **112**, 214304 (2025).

[32] Cesa, F. & Pichler, H. Universal Quantum Computation in Globally Driven Rydberg Atom Arrays. *Phys. Rev. Lett.* **131**, 170601 (2023). 37955503 10.1103/PhysRevLett.131.170601

[33] Hu, Z. & Wu, B. Variational method for wavefunctions in spin-*J* PXP model. Preprint at https://arxiv.org/abs/2501.09301 (2025).

[34] Giudici, G., Surace, F. M. & Pichler, H. Unraveling PXP many-body scars through Floquet dynamics. *Phys. Rev. Lett.* **133**, 190404 (2024). 39576905 10.1103/PhysRevLett.133.190404

[35] Wilkinson, J. W. P., Klobas, K., Prosen, T. & Garrahan, J. P. Exact solution of the Floquet-PXP cellular automaton. *Phys. Rev. E* **102**, 062107 (2020). 33466043 10.1103/PhysRevE.102.062107

[36] Kormos, M., Collura, M., Takács, G. & Calabrese, P. Real-time confinement following a quantum quench to a non-integrable model. *Nat. Phys.* **13**, 246–249 (2017).

[37] Schecter, M. & Iadecola, T. Weak ergodicity breaking and quantum many-body scars in spin-1 XY magnets. *Phys. Rev. Lett.* **123**, 147201 (2019). 31702215 10.1103/PhysRevLett.123.147201

[38] Morettini, G., Capizzi, L., Fagotti, M. & Mazza, L. Transport in a system with a tower of quantum many-body scars. *Phys. Rev. B* **112**, 134314 (2025).

[39] Chandrasekharan, S. & Wiese, U.-J. Quantum link models: a discrete approach to gauge theories. *Nucl. Phys. B* **492**, 455–471 (1997).

[40] Wiese, U.-J. Ultracold quantum gases and lattice systems: quantum simulation of lattice gauge theories. *Ann. der Phys.* **525**, 777–796 (2013).

[41] Hauke, P., Marcos, D., Dalmonte, M. & Zoller, P. Quantum simulation of a lattice Schwinger model in a chain of trapped ions. *Phys. Rev. X* **3**, 041018 (2013).

[42] Maucher, F. et al. Rydberg-induced solitons: three-dimensional self-trapping of matter waves. *Phys. Rev. Lett.* **106**, 170401 (2011). 21635018 10.1103/PhysRevLett.106.170401

[43] Zhao, Y. et al. Three-dimensional solitons in Rydberg-dressed cold atomic gases with spin-orbit coupling. *Sci. Rep.* **13**, 18079 (2023). 37872222 10.1038/s41598-023-44745-9 PMC10593778

[44] Bai, Z., Zhang, Q. & Huang, G. Quantum reflections of nonlocal optical solitons in a cold Rydberg atomic gas. *Phys. Rev. A* **101**, 053845 (2020).

[45] Fleischhauer, M., Imamoglu, A. & Marangos, J. P. Electromagnetically induced transparency: Optics in coherent media. *Rev. Mod. Phys.* **77**, 633–673 (2005).

[46] Vidal, G. Classical simulation of infinite-size quantum lattice systems in one spatial dimension. *Phys. Rev. Lett.* **98**, 070201 (2007). 17358998 10.1103/PhysRevLett.98.070201

[47] Schollwöck, U. The density-matrix renormalization group in the age of matrix product states. *Ann. Phys.* **326**, 96 – 192 (2011).

[48] Fishman, M., White, S. R. & Stoudenmire, E. M. The ITensor Software Library for Tensor Network Calculations. *SciPost Phys. Codebases* 4 (2022).

[49] Fishman, M., White, S. R. & Stoudenmire, E. M. Codebase release 0.3 for ITensor. *SciPost Phys. Codebases* 4–r0.3 (2022).

[50] Forest, E. & Ruth, R. D. Fourth-order symplectic integration. *Phys. D: Nonlinear Phenom.* **43**, 105–117 (1990).

[51] Yoshida, H. Construction of higher order symplectic integrators. *Phys. Lett. A* **150**, 262–268 (1990).

[52] Virtanen, P. et al. SciPy 1.0: Fundamental algorithms for scientific computing in Python. *Nat. Methods* **17**, 261–272 (2020). 32015543 10.1038/s41592-019-0686-2 PMC7056644

[53] Dormand, J. & Prince, P. A family of embedded Runge-Kutta formulae. *J. Comput. Appl. Math.* **6**, 19–26 (1980).

[54] Kerschbaumer, A. Research Data: Quasi-solitons in Rydberg atom chains. Institute of Science and Technology Austria 10.15479/AT-ISTA-21960 (2026). 10.1038/s41467-026-75598-1 PMC13493837 42469244

