# Supplementary material for: Quasi-solitons in Rydberg atom chains
Source: Nat Commun. 2026 Jul 17;17:8783. doi: 10.1038/s41467-026-75598-1 (PMC13493837; doi:10.1038/s41467-026-75598-1)
Supplement: Supplementary file 1 [file 41467_2026_75598_MOESM1_ESM.pdf]

Supplementary Information for "Quasi-solitons in Rydberg atom chains"

Aron Kerschbaumer[1], Jean-Yves Desaules[1], Marko Ljubotina[1,2,3], and Maksym Serbyn[1]
[1]Institute of Science and Technology Austria (ISTA), Am Campus 1, 3400 Klosterneuburg, Austria
[2]Physics Department, Technical University of Munich, TUM School of Natural Sciences, James-Franck-Str. 1, Garching 85748, Germany
[3]Munich Center for Quantum Science and Technology (MCQST), Schellingstr. 4, München 80799, Germany

CONTENTS

Supplementary Note 1. Solitons under unitary dynamics 1
Supplementary Note 2. Energy-carrying solitons and new scarred eigenstates 4
Supplementary Note 3. Spectral decomposition of solitons 5
Supplementary Note 4. TDVP equations of motion and dynamics 6
Supplementary Note 5. Floquet Dynamics 12
Supplementary Note 6. Realization of solitons in Rydberg atom arrays 14
Supplementary Note 7. Solitons in the PPXPP model 15

Supplementary Note 1. SOLITONS UNDER UNITARY DYNAMICS

In Fig. 2**d** of the main text, we have shown the approximate dynamics, during one revival period T, of the scarred initial state $|K\rangle$ and the same state perturbed with a single right-moving cell $|R\rangle$. We validate this picture by numerically simulating a small system with $N = 15$ sites with periodic boundary conditions. We calculate the overlaps of the time-evolved states with the Ansatz states that obey the same structure as proclaimed in the main text figure. The relatively modest system size was chosen to focus on the local dynamics of the soliton and not the scarred cells $|S\rangle$ far away from the soliton and their decay due to imperfect oscillations. The periodic boundary conditions are chosen to avoid the soliton being disturbed by boundary effects. To investigate the structure of $|K(t)\rangle$ we introduce the states:

$$|\psi^0\rangle = |S\rangle^{\otimes 5}, \quad |\psi^{T/4}\rangle = \mathcal{T}\,|R\rangle^{\otimes 5}, \quad |\psi^{T/2}\rangle = \mathcal{T}^2\,|\bar{S}\rangle^{\otimes 5}, \quad |\psi^{3T/4}\rangle = \mathcal{T}\,|L\rangle^{\otimes 5}, \tag{1}$$

where $\mathcal{T}$ is the translation operator that transforms each site $i \to i+1$. The cell $|\bar{S}\rangle$, depicted in yellow in Fig. 2**d** is given by $|\bar{S}\rangle = \frac{1}{\beta}|{\downarrow}\rangle(|{\uparrow\downarrow}\rangle + \beta|{\downarrow\uparrow}\rangle)$. Supplementary Fig. 1**a** shows that the state $|K(mT/4)\rangle$ indeed displays large overlap with the Ansatz state $|\psi^{mT/4}\rangle$ for all integer m, with the corresponding times $mT/4$ highlighted by vertically dashed lines.

For the state with a single right-moving soliton $K^{\mathbf{l},\mathbf{r}}$ with $\mathbf{r} = \{1\}$ and $\mathbf{l} = \varnothing$, we use a similar approach and define the following states:

$$|\psi^0_S\rangle = |R\rangle\,|S\rangle^{\otimes 4}, \quad |\psi^{T/4}_S\rangle = \mathcal{T}\,|\bar{S}\rangle\,|R\rangle^{\otimes 4}, \quad |\psi^{T/2}_S\rangle = \mathcal{T}^2\,|D^1\rangle\,|\bar{S}\rangle^{\otimes 3}, \quad |\psi^{3T/4}_S\rangle = \mathcal{T}\,|L\rangle^{\otimes 2}\,|D^2\rangle\,|L\rangle, \tag{2}$$

where the 6-site defect cells at $T/2$ and $3T/4$ are given by:

$$\begin{aligned}
|D^1\rangle = d_1\,|{\downarrow}\rangle\Big(&0.410\,|{\downarrow\uparrow\downarrow\downarrow\downarrow}\rangle - 0.342\,\mathrm{i}\,|{\downarrow\uparrow\downarrow\downarrow\uparrow}\rangle + 0.176\,\mathrm{i}\,|{\downarrow\uparrow\downarrow\uparrow\downarrow}\rangle + |{\uparrow\downarrow\downarrow\downarrow\downarrow}\rangle \\
&- 0.834\,\mathrm{i}\,|{\uparrow\downarrow\downarrow\downarrow\uparrow}\rangle + 0.247\,\mathrm{i}\,|{\uparrow\downarrow\downarrow\uparrow\downarrow}\rangle + 0.387\,\mathrm{i}\,|{\uparrow\downarrow\uparrow\downarrow\downarrow}\rangle + 0.323\,|{\uparrow\downarrow\uparrow\downarrow\uparrow}\rangle\Big), \\
|D^2\rangle = d_2\,|{\downarrow}\rangle\Big(&0.136\,|{\downarrow\downarrow\downarrow\downarrow\downarrow}\rangle + 0.084\,\mathrm{i}\,|{\downarrow\downarrow\downarrow\downarrow\uparrow}\rangle + 0.139\,\mathrm{i}\,|{\downarrow\downarrow\downarrow\uparrow\downarrow}\rangle + \mathrm{i}\,|{\downarrow\downarrow\uparrow\downarrow\downarrow}\rangle \\
&- 0.614\,|{\downarrow\downarrow\uparrow\downarrow\uparrow}\rangle + 0.113\,\mathrm{i}\,|{\downarrow\uparrow\downarrow\downarrow\downarrow}\rangle - 0.069\,|{\downarrow\uparrow\downarrow\downarrow\uparrow}\rangle + 0.132\,|{\downarrow\uparrow\downarrow\uparrow\downarrow}\rangle \\
&+ 0.071\,|{\uparrow\downarrow\downarrow\uparrow\downarrow}\rangle + 0.389\,|{\uparrow\downarrow\uparrow\downarrow\downarrow}\rangle + 0.239\,\mathrm{i}\,|{\uparrow\downarrow\uparrow\downarrow\uparrow}\rangle\Big),
\end{aligned} \tag{3}$$

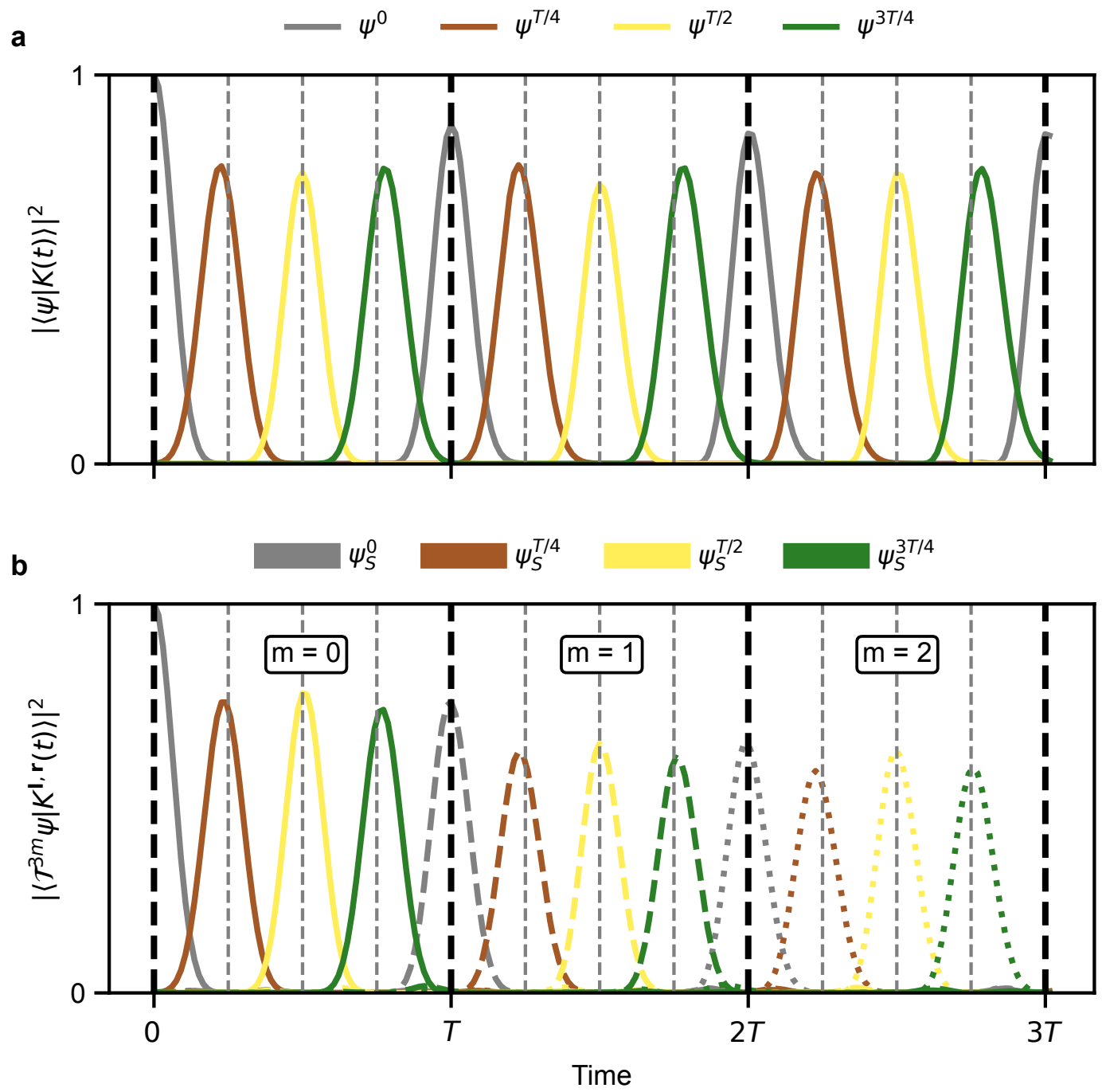

Supplementary Fig. 1. Exact diagonalization simulation of the dynamics of a scarred state without and with right-moving defect cell. The system simulated is a chain with 15 sites with periodic boundary conditions, for which the first three revival periods are shown. Vertical dashed lines indicate times multiple of $T/4$. **a,** The fidelity between $|K(t)\rangle$ and the Ansatz states defined in Eq. (1) show large peaks at multiples of $T/4$. **b,** Similar peaks are visible in the fidelity between the state $|K^{\mathbf{l},\mathbf{r}(t)}(t)\rangle$ ($\mathbf{l}(t) = \varnothing$ $\mathbf{r} = \{1\}$) and the Ansatz states defined in Eq. (2), as well as with the Ansatz states translated by one unit cell, $\mathcal{T}^3$, (dashed lines) and two unit cells, $\mathcal{T}^6$ (dotted lines).

with d_1 and d_2 being normalization constants. The defect cells have been obtained by variationally optimizing generic 6-site states in order to maximize the overlaps between $|\psi_S^{T/2}\rangle$, $|\psi_S^{3T/4}\rangle$ and $K^{\mathbf{l},\mathbf{r}}$ at $T/2$ and $3T/4$ respectively. Even though it is unlikely that we have found the ideal defect cells that yield the maximal overlaps, they already reproduce the proclaimed soliton dynamics. This is visible in Supplementary Fig. 1**b** as pronounced fidelity peaks of the time-evolved state $K^{\mathbf{l},\mathbf{r}(t)}$ and the Ansatz states during the first revival period. Since the R cell shifts by 3 sites to the right during each period T, we also evaluate the overlaps with $\mathcal{T}^3$ as well as $\mathcal{T}^6$ translated Ansatz states (colored dashed and dotted lines respectively).

The coherent propagation of the right-moving soliton, together with the chiral symmetry of the Hamiltonian in the form of $\{H, \mathcal{C}\} = 0$ and the enforced condition on the left-moving soliton $|L\rangle = \mathcal{C}|R\rangle$, ensures the approximate leftwards propagation of $|L\rangle$. Noting that the scarred cell satisfies $\mathcal{C}|S\rangle = |S\rangle$ it follows that $\mathcal{C}K^{\mathbf{l},\mathbf{r}} = K^{\mathbf{r},\mathbf{l}}$, so right-moving soliton cells are mapped to left-moving ones and vice versa. Let us consider a state with a single defect cell $|L\rangle$ at position j, $\mathbf{l} = \{j\}$, $\mathbf{r} = \varnothing$. Then the time evolution of this state for one period T yields indeed:

$$e^{-iHT}|K^{\{j\},\varnothing}\rangle = e^{-iHT}\mathcal{C}|K^{\varnothing,\{j\}}\rangle = \mathcal{C}e^{+iHT}|K^{\varnothing,\{j\}}\rangle \approx \mathcal{C}|K^{\varnothing,\{j-1\}}\rangle = |K^{\{j-1\},\varnothing}\rangle, \tag{4}$$

where the step from the second to the third line relies on the approximate translation of $|R\rangle$ by one unit cell during one period T. Therefore, a coherently right-propagating soliton yields automatically a coherently left-propagating soliton.

That the left-moving soliton cell $|L\rangle$ embedded in the scarred background $|K\rangle$ shows indeed the same coherent propagation as $|R\rangle$ but in the opposite direction can be seen in Supplementary Fig. 2**a** in terms of the number operator and translation fidelity revivals. In the main text, we demonstrated that not only can a single soliton propagate coherently, but a bulk of solitons do as well. We can extend this bulk and consider a state comprising only $|L\rangle$ cells except a single $|S\rangle$ cell. All $|L\rangle$ cells move left by three sites per period; therefore, the single $|S\rangle$ cell is carried along as a defect in the same direction (panel **b** of Supplementary Fig. 2). The heightened translation fidelity revivals are being attributed to the slower decay of the scarred cells $|L\rangle$ compared to $|S\rangle$. However, we emphasize that the defect cells $|L, R\rangle$ only show coherent propagation if they are embedded in the corresponding scarred background. Embedded in a thermal state, they instead lead to incoherent propagation in both directions of

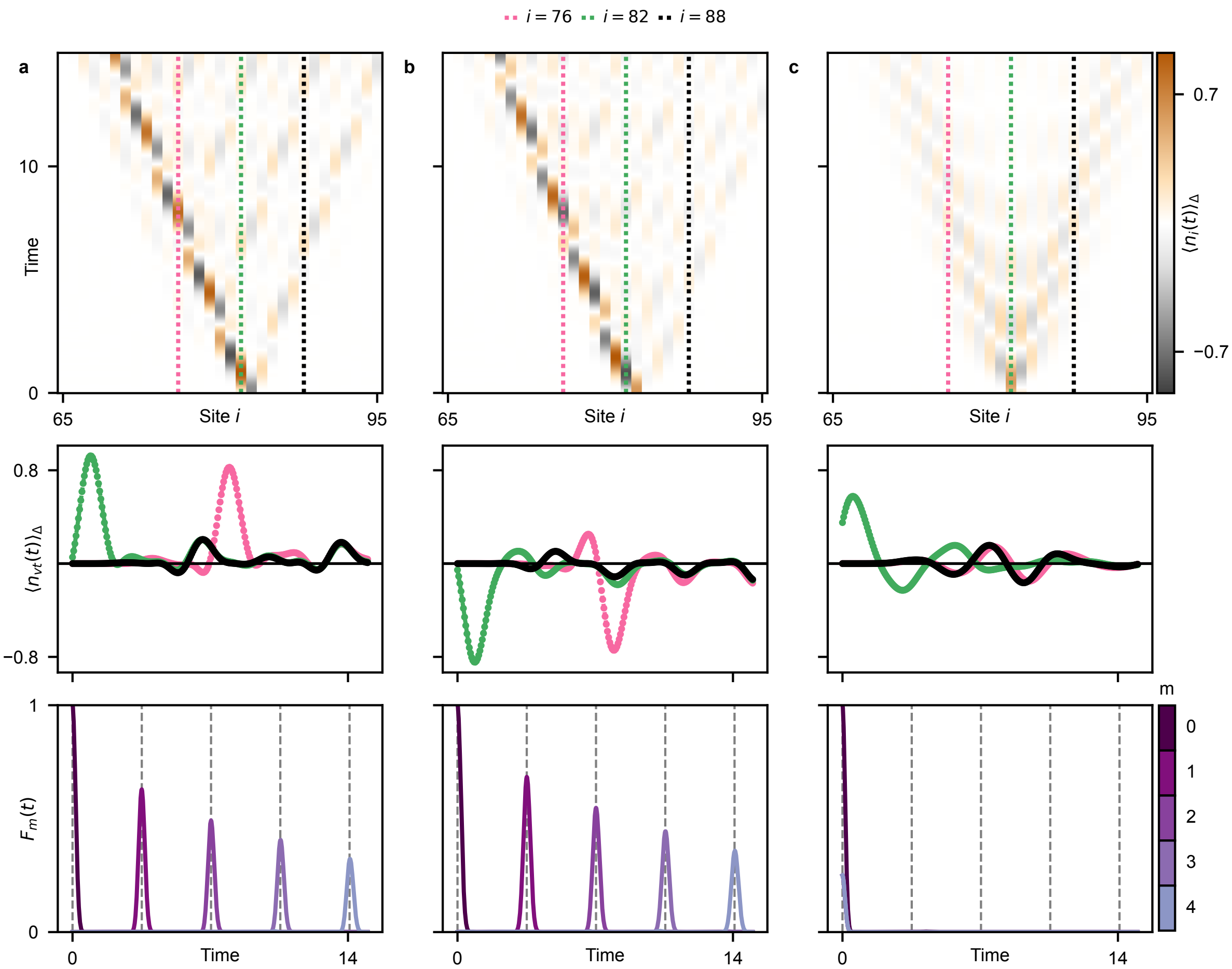

Supplementary Fig. 2. Dynamics of homogeneous initial states with 150 sites perturbed by a single defect cell positioned at sites $\{82, 83, 84\}$. The top row shows the difference between the number operators of the states with and without defect cells. The second row shows the same number operator difference over time for 3 selected sites corresponding to the vertical dashed lines in the top row. The bottom row shows translation fidelities of the corresponding states measured on the subsystem $A = \{61, 62, \ldots, 90\}$. **a,** Scarred state $|K\rangle$ with a defect cell $|L\rangle$ shows coherent propagation of left-moving defect. **b,** The initial state consisting only of left-moving cells $|L\rangle$ except the defect cell $|S\rangle$ show a similar leftwards propagation of the defect cell. **c,** Polarized state consisting of unit cells $|{\downarrow\downarrow\downarrow}\rangle$ where a defect cell $|L\rangle$ is inserted. The defect spreads incoherently in both directions of the chain, and no translation fidelity peak can be observed. In panels **c**, the definition of the translation fidelity is adapted by using $|{\downarrow\downarrow\downarrow}\rangle$ instead of the scarred background state $|S\rangle$.

the chain. This can be seen in Supplementary Fig. 2**c** for the cell $|L\rangle$ inserted in the polarized state $|0\rangle = |{\downarrow\downarrow \ldots \downarrow}\rangle$. While the bidirectional propagation of the defect is clearly visible from the number operator results, its incoherent nature is attested by the lack of peaks in the translation fidelity.

Since the states consisting of the unit cells $|S\rangle$, $|L\rangle$ and $|R\rangle$ lie on the same scarred trajectory (up to translation), the homogeneous states $|K_L\rangle = |L\rangle^{\otimes m}$ and $|K_R\rangle = |R\rangle^{\otimes m}$ show periodic revivals as well, as shown in Supplementary Fig. 3. In comparison, the polarized state $|0\rangle$ shows rapidly decaying fidelity. The state $|K_{\bar{S}}\rangle = |\bar{S}\rangle^{\otimes m}$ shows the same scarred dynamics as $|S\rangle$, $|L\rangle$ and $|R\rangle$ but is omitted for clarity. In fact, we can understand its dynamics and the effect of inserting defects on top of it directly from the symmetries of the Hamiltonian and the structure of the various states. The spatial inversion operator I maps site $i \to N - i + 1$ and commutes with the PXP Hamiltonian. Assuming for simplicity periodic boundary conditions, one can see that the scarred cells $|S\rangle$ and $|\bar{S}\rangle$ are related by $|\bar{S}\rangle = \mathcal{T}\mathcal{I}\,|S\rangle$. Furthermore, the spatially inverted right-moving soliton cell has high overlap with the left-moving cell, up to translation. More specifically, $|\langle L|\mathcal{T}\mathcal{I}|R\rangle|^2 \approx 0.92$. We can define $K^{\mathbf{l},\mathbf{r}}_{|\bar{S}\rangle}$ analogously to Eq. (5) of the main text but formed of unit cells $|\bar{S}\rangle$ instead of $|S\rangle$, while $\mathbf{l}, \mathbf{r}$ again denote the positions of the defect cells $|L, R\rangle$. Considering

such a state with an embedded right-moving defect cell $|R\rangle$ at position j, (i.e. $\mathbf{l} = \varnothing$, $\mathbf{r} = \{j\}$), it will evolve as:

$$e^{-iHT}\,|K_{\bar{S}}^{\varnothing,\{j\}}\rangle = \mathcal{I}e^{-iHT}\mathcal{I}\,|K_{\bar{S}}^{\varnothing,\{j\}}\rangle \approx \mathcal{I}e^{-iHT}\mathcal{T}^{-1}\,|K^{\{N-j+1\},\varnothing}\rangle \approx \mathcal{I}\mathcal{T}^{-1}\,|K^{\{N-j\},\varnothing}\rangle = |K_{\bar{S}}^{\varnothing,\{j+1\}}\rangle\,. \tag{5}$$

As such, $|L\rangle$ and $|R\rangle$ cells placed on top of the scarred state consisting of $|\bar{S}\rangle$ unit cells propagate to the left and right, respectively, as is the case for a background of $|S\rangle$ cells.

In summary, all unit cells attained at multiples of $T/4$ on the scarred trajectory can host coherently propagating defect cells. For $|K\rangle$ and $|K_{\bar{S}}\rangle$, inserting a $|R\rangle$ cell leads to propagation to the right, while inserting $|L\rangle$ leads to propagation to the left. Notably, for $|K_R\rangle$, only right-moving defects can be created by either inserting $|S\rangle$ or $|\bar{S}\rangle$, while the same defects lead to propagation to the left when inserted in $|K_L\rangle$.

A characteristic property of scarred states is their slow growth of entanglement under unitary time-evolution compared to thermal states. While their entanglement entropy still grows linearly in time, as shown in previous works [1, 2], this growth is strongly suppressed. Inserting soliton defect cells into the scarred background does not enhance the entanglement growth of the scarred state significantly, as can be seen in Supplementary Fig. 4. The evolution of the half-chain bipartite entanglement entropy $S_{\rm ent} = -\,{\rm Tr}\,[\rho_A \ln \rho_A]$ is being measured for different states $K_{|\bar{S}\rangle}^{\mathbf{l},\mathbf{r}}$ and all of them show oscillatory dynamics that is connected to reviving of the scarred cells, while a heightened entanglement peak occurs when the solitons pass through the observed cut. Moreover, these states show strongly suppressed entanglement growth compared to the thermal polarized state $|0\rangle$. Even the state showing a collision between left- and right-moving solitons $|\ldots SRSSLS\ldots\rangle$ still features a suppressed entanglement growth.

Supplementary Note 2. ENERGY-CARRYING SOLITONS AND NEW SCARRED EIGENSTATES

Energy-carrying solitons $|R^{\pm}\rangle$ and $|L^{\pm}\rangle$ embedded in the scarred state $|K\rangle$ propagate coherently and show increased stability compared to their counterparts with zero energy, as shown in the main text. To better understand the interaction between energy-solitons and their decay, we are showing the local energy density of different multi-soliton configurations in Supplementary Fig. 5 and plot the energy densities along three different world-lines. As expected, the energy density along a soliton trajectory without collision decays very slowly, while the energy density experiences an additional decay during collision.

Energy-carrying soliton cells $|R^{\alpha}\rangle$ can be used to create homogeneous scarred states with tunable energy of the form $|K_{\alpha}\rangle = |R^{\alpha}\rangle^{\otimes l}$. The state returns approximately to its initial configuration because each cell shifts 3 sites to the right per period (scarred states can be formed analogously by using $|L^{\alpha}\rangle$). The energies at each site are given by:

$$\langle R^{\alpha}|X_1|R^{\alpha}\rangle = 0, \quad \langle R^{\alpha}|X_2|R^{\alpha}\rangle = \sqrt{\tfrac{1}{2+2/\beta^2}}\,\sin(2\alpha)\,, \quad \langle R^{\alpha}|X_3|R^{\alpha}\rangle = \sqrt{\tfrac{1}{2+2\beta^2}}\,\sin(2\alpha)\,, \tag{6}$$

leading to average energy per site $\langle\bar{X}\rangle$ between -0.33 and 0.33 for the value of $\beta = 0.65$ that we use. For the ground and ceiling states, the same quantity takes values of ± 0.60 respectively. Thus, the states $|R^{\alpha}\rangle$ and $|L^{\alpha}\rangle$ are always at

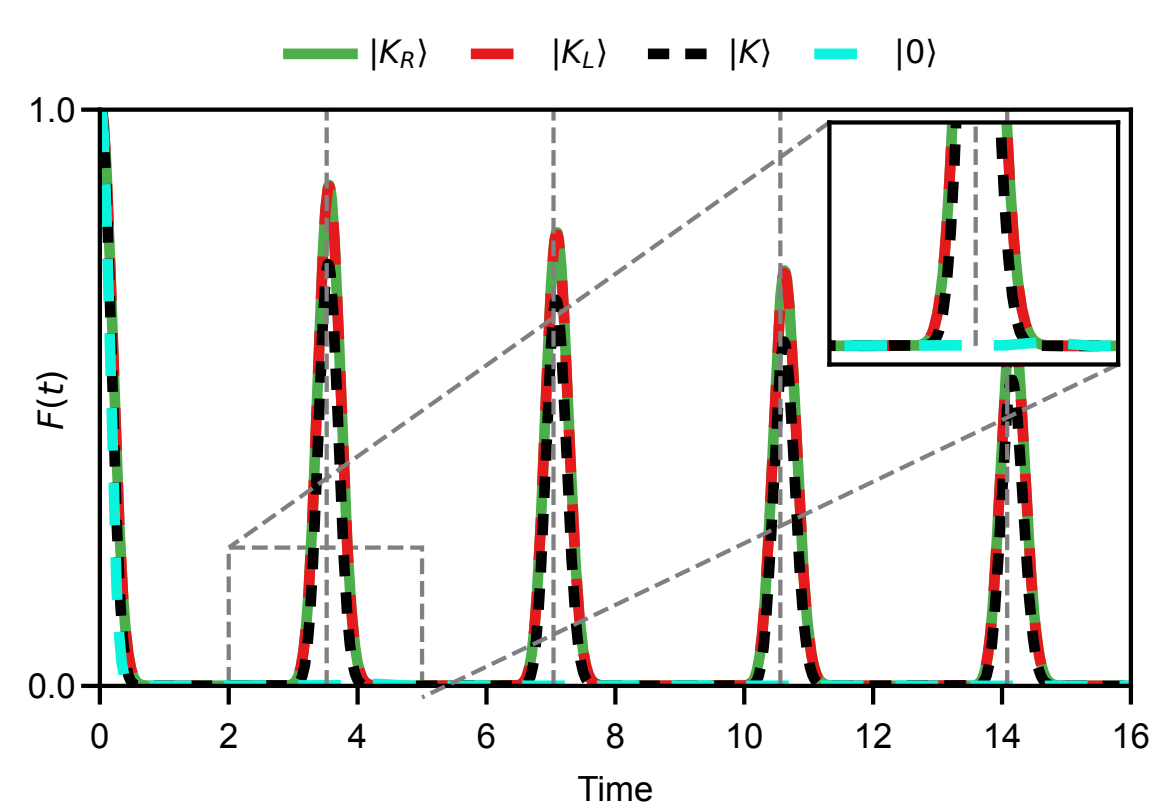

Supplementary Fig. 3. Time-evolution of different homogeneous initial states defined on a 150-site Rydberg chain. The states $|K_R\rangle = |R\rangle^{\otimes 50}$, $|K_L\rangle = |L\rangle^{\otimes 50}$ and $|K\rangle = |S\rangle^{\otimes 50}$ show scarred dynamics in form of large fidelity revivals at multiples of period T (identified by vertically dashed lines). By comparison the polarized state $|0\rangle = |\downarrow\rangle^{\otimes 150}$ shows fast fidelity decay without revivals. Fidelities are being measured on the subsystem $A = \{61, 62, \ldots 90\}$.

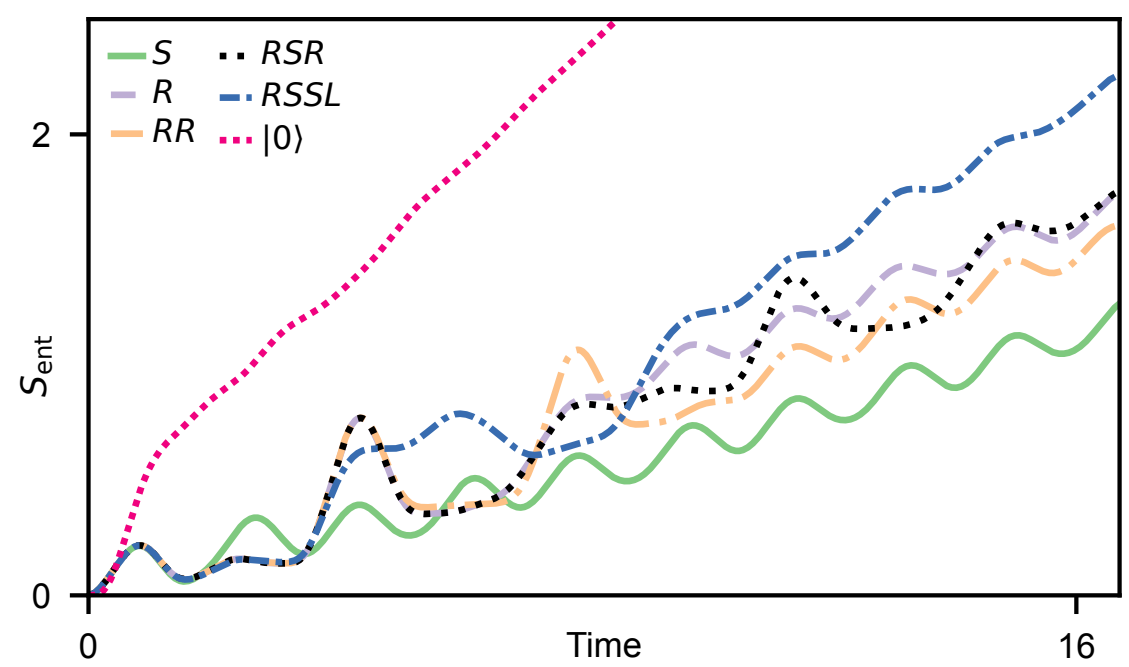

Supplementary Fig. 4. Half-chain bipartite entanglement dynamics of different $|K^{\mathbf{l},\mathbf{r}}\rangle$ states defined on a 120-site chain with the bipartition cut chosen between sites 60 and 61. The states show oscillatory entanglement dynamics, with additional peaks when the soliton passes through the cut, except for the state $RSSL$, which leads to a disruption of the oscillatory entanglement growth. The entanglement growth of all $|K^{\mathbf{l},\mathbf{r}}\rangle$ states is suppressed compared to the polarized state $|0\rangle$. The label S corresponds to the scarred states $|K\rangle$. Label R: $\mathbf{l} = \varnothing$ and $\mathbf{r} = \{19\}$. Label RR: $\mathbf{l} = \varnothing$ and $\mathbf{r} = \{18, 19\}$, Label RSR: $\mathbf{l} = \varnothing$ and $\mathbf{r} = \{17, 19\}$, Label $RSSL$: $\mathbf{l} = \{22\}$ and $\mathbf{r} = \{19\}$

.

finite energy density away from the edges of the spectrum; therefore, excluding low-energy phenomena as a possible explanation for their revivals. Periodic revivals for states with different energy can be seen in Supplementary Fig. 6. While the fidelity decays faster than for zero-energy states, there are still strong signatures of ergodicity breaking for *all* values of α.

Supplementary Note 3. SPECTRAL DECOMPOSITION OF SOLITONS

Periodic revivals of scarred states are usually attributed to large overlaps with a set of special, equidistantly spaced, eigenstates [1, 3–5]. Indeed, in agreement with this intuition, the zero-energy state $|K\rangle$ shows high overlap with a band

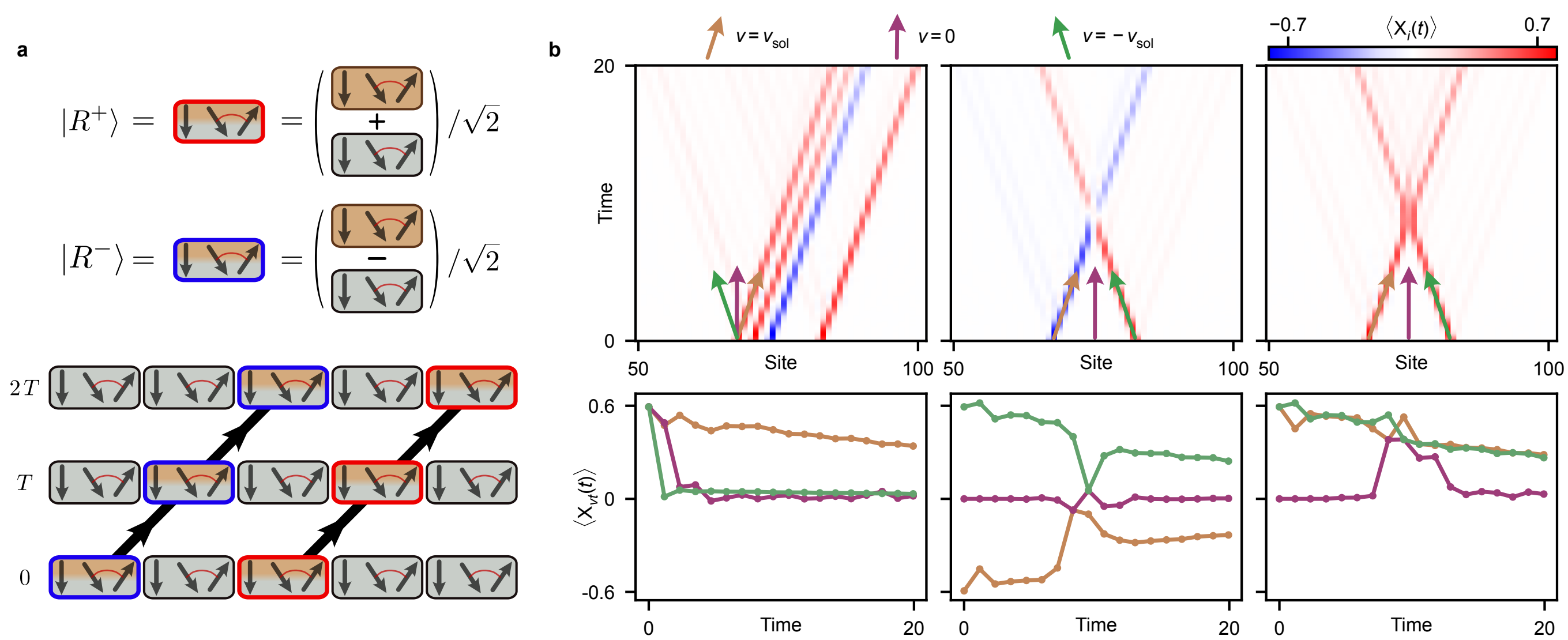

Supplementary Fig. 5. Time evolution of the energy density for three different energy-carrying multi-soliton states. Top: energy density dynamics shown as a function of position and time highlight the chiral propagation of energy. Bottom: energy density along world-lines with velocities $v = 0, \pm v_{\mathrm{sol}}$, with $v_{\mathrm{sol}} = 3/T \approx 0.85$. Without collisions (**a**), more than 50% of the original energy is retained in the soliton even at late times, whereas collisions (**b** and **c**) lead to an additional decrease in the energy density along the soliton trajectory. All three different initial states are defined on 150 sites and are given by (from left to right):
$|S\rangle^{\otimes 22}|R^+\rangle|R^+\rangle|R^-\rangle|S\rangle|S\rangle|R^+\rangle|S\rangle^{\otimes 22}$, $|S\rangle^{\otimes 22}|R^-\rangle\,|S\rangle^{\otimes 4}|L^+\rangle|S\rangle^{\otimes 22}$ and $|S\rangle^{\otimes 22}|R^+\rangle\,|S\rangle^{\otimes 4}|L^+\rangle|S\rangle^{\otimes 22}$.

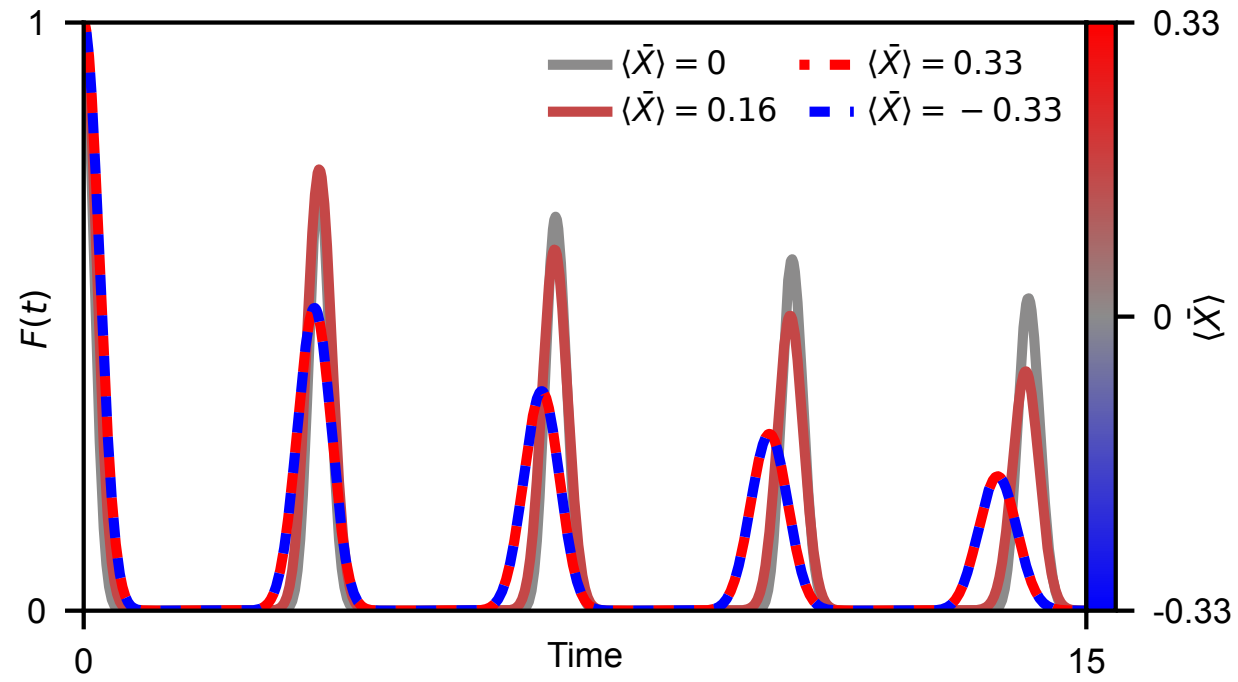

Supplementary Fig. 6. Time-evolution for various scarred states $|\psi\rangle = |R^\alpha\rangle^{\otimes 30}$ with different mean energies $\langle\bar{X}\rangle$ (average is taken over all sites i) defined on 90 sites. $\langle\bar{X}\rangle = 0$ corresponds to $|S\rangle$ ($\alpha = \pi/2$) unit cells, $\langle\bar{X}\rangle = \pm 0.33$ to $|R^\pm\rangle$ ($\alpha = \pm\pi/4$) and $\langle\bar{X}\rangle = 0.16$ to $\alpha = 0.26$. Pronounced periodic fidelity revivals can be observed for scarred states with all energies, while the revivals for scarred states with extremal energy densities are lower. The fidelities are being measured on the subsystem $A = \{31, 32, \ldots 60\}$.

of special eigenstates that is symmetrically distributed around the center of the spectrum, as visible in Supplementary Fig. 7**a**. Likewise, the scarred state $|K_{\pi/4}\rangle$ built from repeating soliton cells $|R^+\rangle$ has enhanced overlaps with a band of special eigenstates that now all have positive energies, see Supplementary Fig. 7**b**. We emphasize that these eigenstates are fully distinct from the ones responsible for the scarring observed in the $|\mathbb{Z}_2\rangle$ state. Additionally, we note that these eigenstates would be hard to identify from entanglement entropy alone, as their entropies increase progressively as their energy approaches zero.

The expansion of an initial state that has a single soliton cell $|R\rangle$ inserted in the scarred background, shown in Supplementary Fig. 7**c**, is, however, more intricate. First, overlaps with this initial state single out a few eigenstates at positive and negative energies. These dominant eigenstates occur at the same energies as the ones responsible for scarring from the $|K\rangle$ state, and several of them are actually the same for both initial states. However, while eigenstates closer to edges of the spectrum have somewhat enhanced overlaps compared to other states at similar energies, visible as "gap" in Supplementary Fig. 7**c** separating them from the rest of the states, this gap is missing near the center of the spectrum. As such, compared to the $|S\rangle$ state, many more eigenstates contribute to the dynamics when a defect cell is introduced. This is expected based on symmetry considerations: for the $|K\rangle$ state, only the $k = 0$ and $k = \pm\frac{2\pi}{3}$ momentum sectors contribute, whereas for single-defect states *all* symmetry sectors are relevant. These results suggest that, while the states with solitons retain high overlap with the eigenstates linked to the scarred background $|S\rangle$, there is no simple subset of eigenstates responsible for soliton dynamics. Although we cannot exclude the possibility that some weak deformation of the PXP model may stabilize solitons and reveal a simpler structure in their eigenstate expansions.

Supplementary Note 4. TDVP EQUATIONS OF MOTION AND DYNAMICS

The following optimization procedure has been applied in order to find the soliton and scarred angles, denoted by vectorized notations as $\boldsymbol{\theta}^S = (\theta_1^S, \theta_2^S, \theta_3^S)$, and $\boldsymbol{\theta}^R = (\theta_1^R, \theta_2^R, \theta_3^R)$, respectively. The simulation has been performed on a system with a unit cell of size 15, in which the following two sets of angles are defined:

$$\boldsymbol{\theta}^{\text{init}} = (\boldsymbol{\theta}^S, \boldsymbol{\theta}^R, \boldsymbol{\theta}^S, \boldsymbol{\theta}^S, \boldsymbol{\theta}^S), \qquad \boldsymbol{\theta}^{\text{shift}} = (\boldsymbol{\theta}^S, \boldsymbol{\theta}^S, \boldsymbol{\theta}^R, \boldsymbol{\theta}^S, \boldsymbol{\theta}^S).$$

The EOMs for the initial state defined by the angles $\boldsymbol{\theta}^{\text{init}}$ are integrated up to the final time $t = 20$, resulting in a function $\boldsymbol{\theta}(t)$ for $t \in [0, 20]$.

We defined a total loss functional operating on $\boldsymbol{\theta}(t)$, L, that contains two parts,

$$L = L_{\text{closed}} + L_{\text{shifted}}.$$

The first part measures how well the trajectory returns to its initial state after the soliton has traveled one full period through the chain, and the second part measures how accurately all angles have shifted by 3 sites to the right after

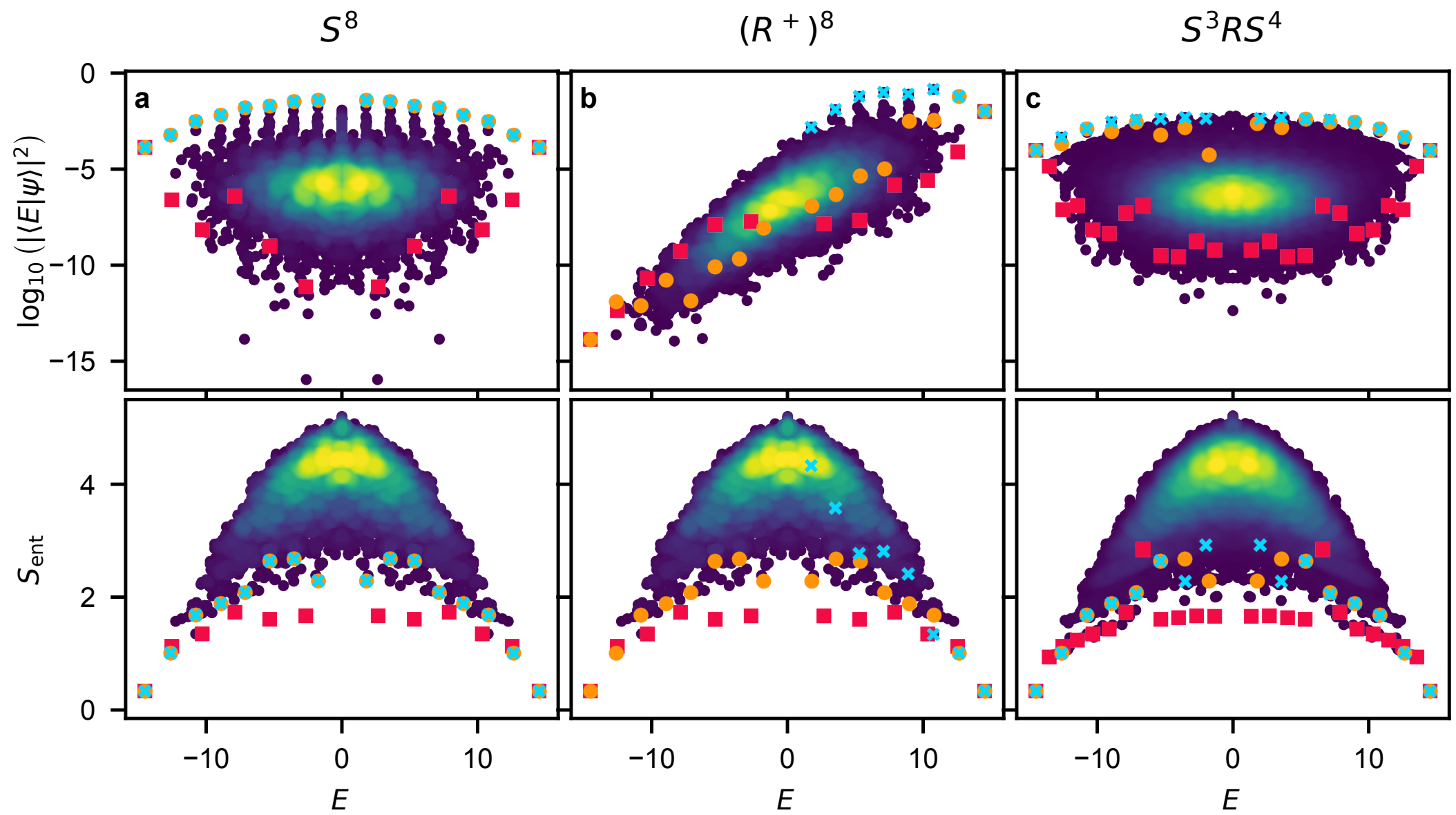

Supplementary Fig. 7. Overlap of eigenstates with different scarred states with and without defects (top row) and half-chain von Neumann entanglement entropy of eigenstates (bottom row). The states are defined on a chain of 24 sites with periodic boundary conditions. Only eigenstates in the symmetry sectors relevant for each state are shown ($k = 0$ and $k = \pm\frac{2\pi}{3}$ in panels **a** and **b** and all momentum sectors for panel **c**). The color indicates the density of data points, going from dark blue to yellow as density increases. The red squares indicate the scarred eigenstates linked to the $|\mathbb{Z}_2\rangle$ state, while the orange circles indicate the ones linked to the entangled $|\mathbb{Z}_3\rangle$ state $|K\rangle = |S \cdots S\rangle$. Finally, the light blue crosses indicate the most likely candidate for scarred eigenstates for the state considered in each panel. They are identified as the eigenstates with the highest overlap in their energy window. For the three different highlighted set of states, the same eigenstates are also highlighted in the same way in the bottom row. **a,** The scarred state $|K\rangle$ has high overlap with a band of approximately evenly spaced eigenstates that is well separated from the bulk, symmetric around zero energy, and has low entanglement. **b,** Scarred state with maximal positive energy with unit cell $|R^+\rangle$ has increased overlaps with higher energy eigenstates. A band of special eigenstates with positive energy shows very high overlap with the scarred states and is well separated from the other eigenstates. **c,** The state $K^{\mathbf{l},\mathbf{r}}$ with a single right-moving defect ($\mathbf{l} = \varnothing$, $\mathbf{r} = \{4\}$) shows several eigenstates with heightened overlap that have low entanglement. However, these states are not very well separated from the bulk of the eigenstates.

one scar revival period. The first part of the loss functional is defined as:

$$L_{\text{closed}} = \min_{t\in[15,20]} \sum_{i=1}^{15} \Delta_i\big(t; \boldsymbol{\theta}^{\text{init}}\big) + P_{\text{closed}}, \tag{7}$$

$$\Delta_i(t; \mathbf{v}) = \left|\cos^2\theta_i(t) - \cos^2 v_i\right| + \left|\sin^2\theta_i(t) - \sin^2 v_i\right|. \tag{8}$$

$$P_{\text{closed}} = \begin{cases} \cos^2\!\big(\frac{\pi}{2}\, d/0.4\big), & d < 0.4, \\ 0, & d \geq 0.4, \end{cases} \tag{9}$$

$$d = \sum_{1\leq i<j\leq 3} \big[\,\big|\,|\cos\theta_i^S| - |\cos\theta_j^S|\,\big| + \big|\,|\sin\theta_i^S| - |\sin\theta_j^S|\,\big|\,\big], \tag{10}$$

where $\Delta_i(t; \mathbf{v})$ is a helper function that defines how we measure the distance between angles. It takes an angle vector $\mathbf{v}$ as input and measures its distance to the time-evolved angles in the i-th coordinate. Consequently, the first term of L_{closed} measures how close the trajectory returns to its initial conditions in the expected time window ($5\cdot 3 \leq t \leq 5\cdot 4$) in which the soliton travels through all 5 cells (time window of scar reviving period was chosen to be $3 \leq t \leq 4$). P_{closed} is a penalty that is introduced to ensure that the angles of the scarred cells are not all identical to each other, since this leads to solutions that yield relatively low losses but do not show solitonic dynamics. The functional form of P_{closed} is chosen to ensure a smooth penalty, and the cutoff value 0.4 was empirically found to be a good value.

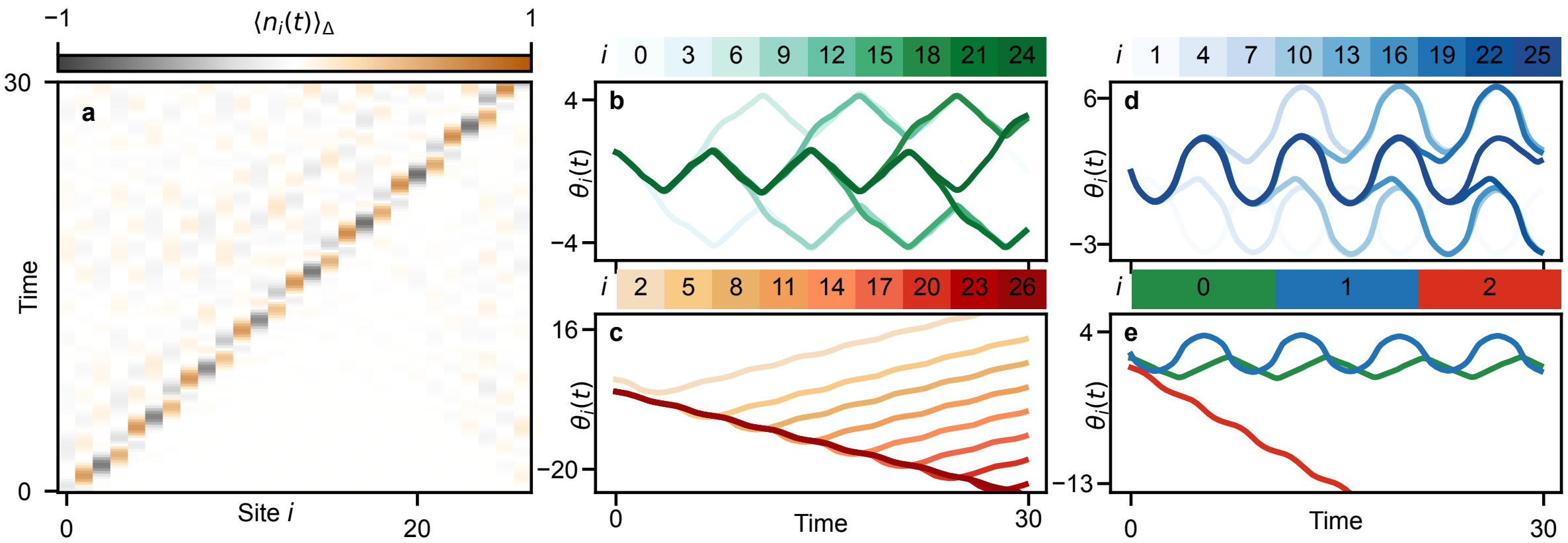

Supplementary Fig. 8. Time evolution of classical equations of motion (9) of an infinite-chain with a 27-site unit cell up to time $t = 30$. The initial state has all sites initialized with $\theta^S_{1,2,3}$ angles except for the sites $\{1, 2, 3\}$ initialized with right-moving soliton $\theta^R_{1,2,3}$ angles. **a,** The characteristic soliton propagation can be observed in the expectation value of the number operator density between states with and without a soliton cell. **b-d** Evolution of angles of the first and second site of each 3-site shows approximately periodic dynamics up to aperiodic shifts induced when the soliton crosses the unit-cell. **e** Periodic dynamics of the three scarred angles for the initial state without the soliton cell.

The second part has a somewhat simpler form as

$$L_{\text{shifted}} = \min_{t\in[3,4]} \sum_{i=1}^{15} \Delta_i\big(t;\boldsymbol{\theta}^{\text{shift}}\big) + P_{\text{shifted}}. \tag{11}$$

$$P_{\text{shifted}} = \begin{cases} 3\cos^2\!\big(\frac{\pi}{2}\, D/0.7\big), & D \le 0.7, \\ 0, & D > 0.7, \end{cases} \tag{12}$$

$$D = \sum_{a=1}^{3} \big(|\cos^2\theta^S_a - \cos^2\theta^R_a| + |\sin^2\theta^S_a - \sin^2\theta^R_a|\big), \tag{13}$$

where the first term in L_{shifted} measures the distance between the time-evolved angles and $\boldsymbol{\theta}^{\text{shift}}$ in the time window of the expected scar reviving period. The penalty term P_{shifted} ensures that the parameters of the soliton cell are different from those of the scarred cells. The function is again chosen to be smooth, and the cutoff value 0.7 was chosen empirically.

We have run this minimization procedure several times and used the parameters that most convincingly produced the solitonic dynamics, which are provided in the Methods section. Numerical integration reveals that these equations feature many singular points, originating from the PXP constraint. Some of these singularities occur close to the angles corresponding to the scarred cells, which makes the optimization very challenging, especially for gradient-based optimizers. We used SciPy's stochastic differential evolution algorithm [6, 7] for this purpose. We note that it is likely that better initial configurations – i.e., initial configurations leading to more coherent solitonic dynamics or even closed trajectories – exist.

In Fig. 5 of the main text, we have established the existence of solitons within our variational description. To get a better understanding of the evolution of the angles $\theta_i(t)$ in a classical dynamical system, we revisit the same initial state (with a smaller unit cell) in Supplementary Fig. 8 and plot the evolution of all angles. In panel **a**, the number operator density clearly shows the coherent propagation of the soliton. Panels **b-c** show the periodic motion of the first and second angles of each unit cell correspondingly, until the soliton passes through, temporarily disturbing this periodic motion, and shifting the angle to a replica of the periodic trajectory. The third angle in each cell shown in panel **d** corresponds to a winding angle whose direction reverses as the soliton passes through. Panel **e** shows the characteristic periodic dynamics of the purely scarred initial state without solitons. As discussed in the main text this state is very close to a point on the trajectory of $|K\rangle$ which is the trajectory discussed in Ref. [8].

In the main text, it has been shown that the classical EOMs (13) and (14) derived via TDVP are capable of capturing solitonic dynamics. The scarred background cell $\theta^S_{1,2,3} = (1.05977133, 1.46820419, 0.0000131027276)$

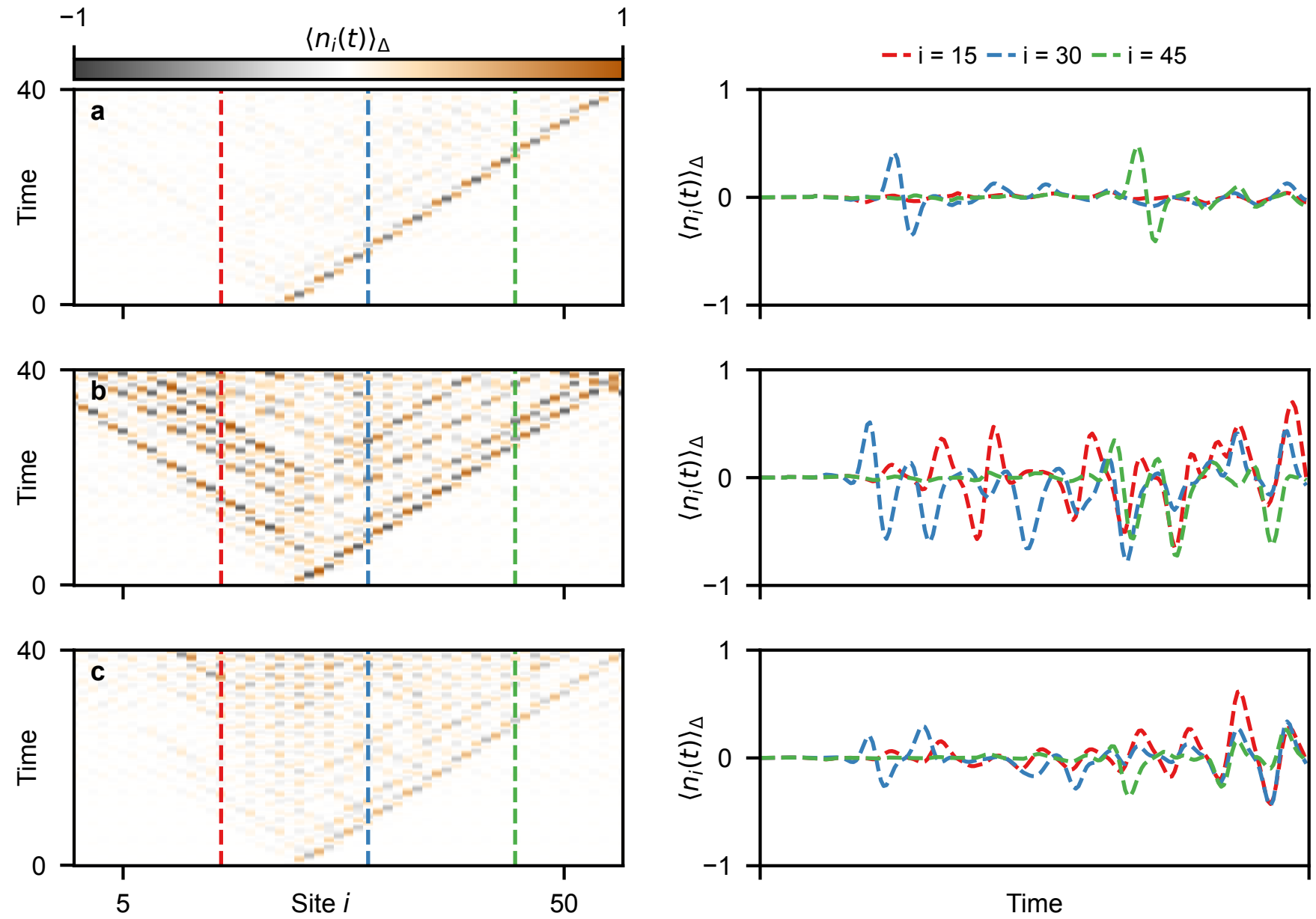

Supplementary Fig. 9. Time evolution of classical EOMs (13) and (14) of the main text for different scarred initial states defined using a 57-site unit cell with a single defect cell inserted at sites $\{22, 23, 24\}$. The left column shows the evolution of the number operator densities with and without the defect cells. The right column shows the evolution of the number operator at three specific sites. **a,** Dynamics of the numerically optimized state shown in the main text with angles of scarred cell given by $\theta^S_{1,2,3} = (1.05977133, 1.46820419, 0.0000131027276)$ and defect cell by $\theta^R_{1,2,3} = (0.990853599, 0.454003269, 3.10125177)$. The defect cell propagates coherently even at later times. **b,** Dynamics of the scarred state $|S\rangle$ corresponding to the angles $\tilde{\theta}^S_{1,2,3} = (0.0, 0.57637521, \pi/2)$ with a right-moving soliton $|R\rangle$ corresponding to $\tilde{\theta}^R_{1,2,3} = (0.0, 2.5069758, 2.64260532)$. Unlike in the fully quantum case, this state shows fast decay of the soliton and subsequent incoherent propagation in both directions. **c,** Dynamics of the scarred state $|S\rangle$ given by $\tilde{\theta}^S_{1,2,3} = (0.0, 0.57637521, \pi/2)$ with right-moving energy-carrying soliton $|R^+\rangle$ given by $\tilde{\theta}^{R^+}_{1,2,3} = (0.0, 2.53577767, 2.22464348)$ and $\tilde{\phi}^{R^+}_{1,2,3} = (0.0, 0.82745493, 2.71076968)$. While coherent propagation is visible at early times, it eventually turns incoherent.

and the right-moving defect cell $\theta^R_{1,2,3} = (0.990853599, 0.454003269, 3.10125177)$ have been numerically optimized to achieve coherent propagation of the solitonic defect. However, the solitonic defects $|L, R\rangle$ and $|L^\pm, R^\pm\rangle$ embedded in the scarred background state $|K\rangle$ can also be faithfully represented via the Ansatz in Eq. (11) of the main text, allowing their time-evolution to be studied within the semiclassical description. The angles for the scarred cell $|S\rangle$ are given by $\tilde{\theta}^S_{1,2,3} = (0.0, 0.57637521, \pi/2)$, for $|L\rangle$ and $|R\rangle$ by $\tilde{\theta}^L_{1,2,3} = (0.0, 2.50697563, 0.49898734)$ and $\tilde{\theta}^R_{1,2,3} = (0.0, 2.5069758, 2.64260532)$. The energy-carrying soliton cells, which have phase angles $\phi \neq \pi/2$, are given by $\tilde{\theta}^{R^+}_{1,2,3} = (0.0, 2.53577767, 2.22464348)$, $\tilde{\phi}^{R^+}_{1,2,3} = (0.0, 0.82745493, 2.71076968)$ and $\tilde{\theta}^{R^-}_{1,2,3} = (0.0, 2.53577767, 2.22464348)$, $\tilde{\phi}^{R^-}_{1,2,3} = (0.0, 2.31413787, 0.43082226)$ as well as $\tilde{\theta}^{L^+}_{1,2,3} = (0.0, 2.53577818, 0.91695008)$, $\tilde{\phi}^{L^+}_{1,2,3} = (\pi/2, 2.31414114, 0.43082552)$ and $\tilde{\theta}^{L^-}_{1,2,3} = (0.0, 2.53577773, 0.91694925)$, $\tilde{\phi}^{L^-}_{1,2,3} = (0.0, 0.82745289, 2.71076687)$.

In Supplementary Fig. 9**b** we show that $|R\rangle$ embedded in the scarred state background cells $|S\rangle$ initially shows solitonic dynamics but quickly loses coherence compared to the numerically optimized state in Supplementary Fig. 9**a**. The energy-carrying soliton (Supplementary Fig. 9**c**) shows coherent propagation for longer times but still does not match the coherent propagation of the numerically optimized state.

A numerically optimized left-moving defect cell is given by the angles $\theta^L_{1,2,3} = (4.13244625, 3.59559592, 6.24284442)$. Numerically optimized left- and right-moving solitons interact strongly upon collision, deform the scarred background, and lose their coherence as shown in Supplementary Fig. 10**a**. The collision between the energy-carrying solitons $|L^+\rangle$ and $|R^+\rangle$ appears to be more stable (see Supplementary Fig. 10**b**), as solitons continue to propagate without a phase shift and no significant decrease in energy after the collision. Nevertheless, the collision strongly distorts the scarred background.

So far, we have considered states with solitonic excitations that show coherent propagation under the exact dynamics

of the quantum model, and have investigated their evolution in the system of classical EOMs. Conversely, we will now take the soliton that we have numerically optimized in the semiclassical limit and investigate its dynamics under unitary time-evolution of the quantum model. Using the MPS Ansatz given in Eq. (11) of the main text (fixing all phase angles $\phi = \pi/2$), we define the scarred state via the numerically optimized angles $\theta^S_{1,2,3}$ as

$$|\psi_S\rangle = \sum_{\{\sigma\}} V_L \prod_{l=0}^{N/3-1} A^{\sigma_{1+3l}}(\theta_1^{\mathrm{S}})\, A^{\sigma_{2+3l}}(\theta_2^{\mathrm{S}})\, A^{\sigma_{3+3l}}(\theta_3^{\mathrm{S}})\, V_R\ |\sigma_1\sigma_2\ldots\sigma_N\rangle\,, \tag{14}$$

and the state with the incorporated soliton defect cell $\theta^R_{1,2,3}$ as

$$\begin{aligned}|\psi_R\rangle = \sum_{\{\sigma\}} & V_L\Big[\prod_{l=0}^{l_0-1} A^{\sigma_{1+3l}}(\theta_1^{\mathrm{S}})\, A^{\sigma_{2+3l}}(\theta_2^{\mathrm{S}})\, A^{\sigma_{3+3l}}(\theta_3^{\mathrm{S}})\Big] \\ & \times A^{\sigma_{3l_0+1}}(\theta_1^{\mathrm{R}})\, A^{\sigma_{3l_0+2}}(\theta_2^{\mathrm{R}})\, A^{\sigma_{3l_0+3}}(\theta_3^{\mathrm{R}}) \\ & \times \Big[\prod_{l=l_0+1}^{N/3-1} A^{\sigma_{1+3l}}(\theta_1^{\mathrm{S}})\, A^{\sigma_{2+3l}}(\theta_2^{\mathrm{S}})\, A^{\sigma_{3+3l}}(\theta_3^{\mathrm{S}})\Big]V_R\ |\sigma_1\sigma_2\ldots\sigma_N\rangle\,,\end{aligned} \tag{15}$$

where the physical indices σ label the on-site Hilbert space basis $(\uparrow, \downarrow)$. V_L and V_R are the left and right boundary vectors, chosen to be $(1,0)^T$ and $(1,1)$ respectively, and l_0 defines the position of the soliton cell. The cells of the MPSs are weakly entangled and have no product-like structure. We measure their similarity with regard to the cells that show scarred and solitonic under unitary time-evolution of the quantum model by measuring the projector expectation values. It turns out the numerically optimized scarred state is locally almost identical to the state $|\bar{S}\rangle$ that occurs along the scarred trajectory. Indeed, we find that in a system with 153 sites, $\langle\psi_S|P_{\bar{S},75}|\psi_S\rangle \approx 0.99$, where $P_{\bar{S},j} = |\bar{S}\rangle\langle\bar{S}|$ is the projector on the $\bar{S}$ cell at position j to $j+2$. Analogously, we are measuring the expectation value of $P_{R,75} = |R\rangle\langle R|$ with respect to $|\psi_R\rangle$. The position of the defect angles $\{3l_0+1, 3l_0+2, 3l_0+3\}$ coincides with $\{75, 76, 77\}$ and the expectation value yields $\langle\psi_R|P_{R,75}|\psi_R\rangle \approx 0.56$. Finally, time-evolving $|\psi_R\rangle$ under unitary dynamics of the quantum model leads the defect to propagate coherently to the right with only modest deformation of the scarred background (Supplementary Fig. 11). Large peaks of the translation fidelities reveal that the defect cell approximately maintains its structure. The revivals of all $F_m(t)$ at multiples of T reveal finite overlap of the defect and the scarred cell. Solitonic excitations in the classical EOMs appear to be more stable when they have finite overlap with the scarred cell.

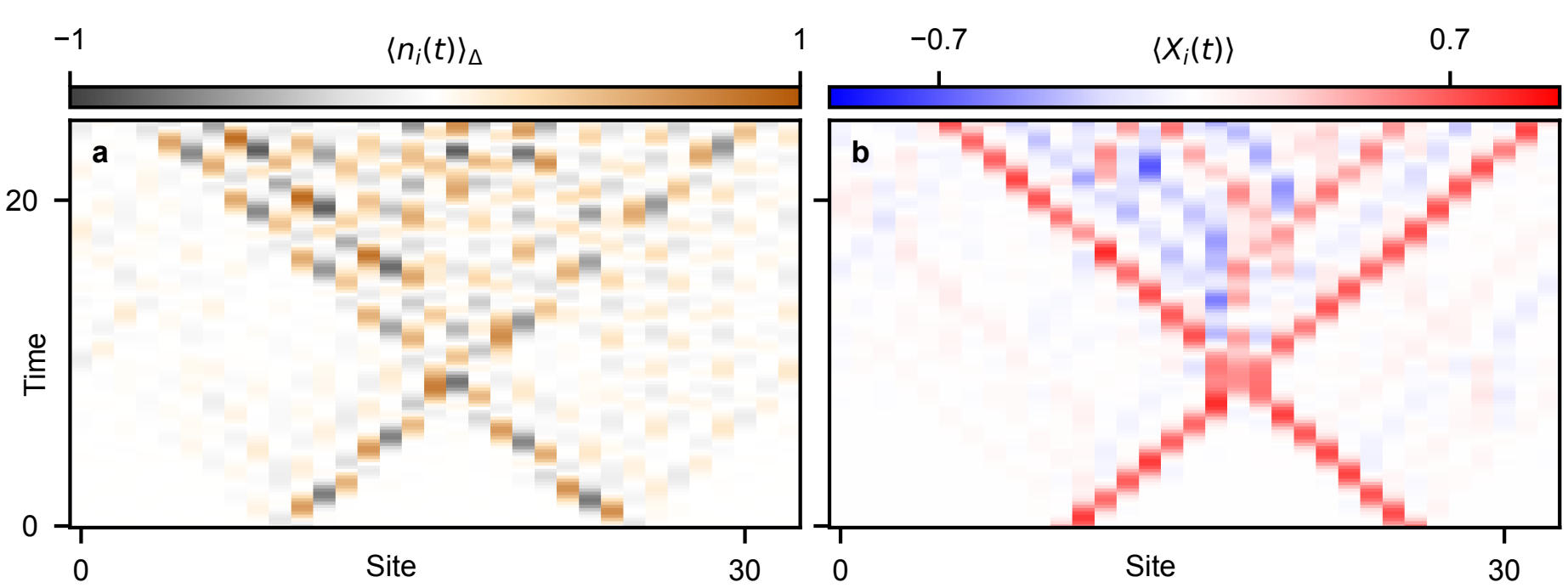

Supplementary Fig. 10. Time evolution of classical EOMs (13), (14) of the main text showing collisions of solitons propagating in opposite directions on top of a scarred background state defined on 33 sites. **a,** Numerically optimized zero-energy solitons with the scarred background cells defined via the angles $\theta^S_{1,2,3} = (1.05977133, 1.46820419, 0.0000131027276)$ and a right-moving soliton cell defined via the angles $\theta^R_{1,2,3} = (0.990853599, 0.454003269, 3.10125177)$ on the sites $\{10, 11, 12\}$ and a left-moving soliton with angles $\theta^L_{1,2,3} = (4.13244625, 3.59559592, 6.24284442)$ on the sites $\{25, 26, 27\}$. Solitons are strongly interacting during the collision and perturb the scarred background. **b,** Energy solitons on top of the usual scarred background cells $|S\rangle$ with angles $\tilde{\theta}^S_{1,2,3} = (0.0, 0.57637521, \pi/2)$ and a right-moving energy soliton cell $|R^+\rangle$ given via $\tilde{\theta}^{R^+}_{1,2,3} = (0.0, 2.53577767, 2.22464348)$ and $\tilde{\phi}^{R^+}_{1,2,3} = (0.0, 0.82745493, 2.71076968)$, positioned at sites $\{10, 11, 12\}$ and a left-moving energy soliton cell $|L^+\rangle$ given via $\tilde{\theta}^{L^+}_{1,2,3} = (0.0, 2.53577818, 0.91695008)$ and $\tilde{\phi}^{L^+}_{1,2,3} = (\pi/2, 2.31414114, 0.43082552)$, positioned at sites $\{25, 26, 27\}$.

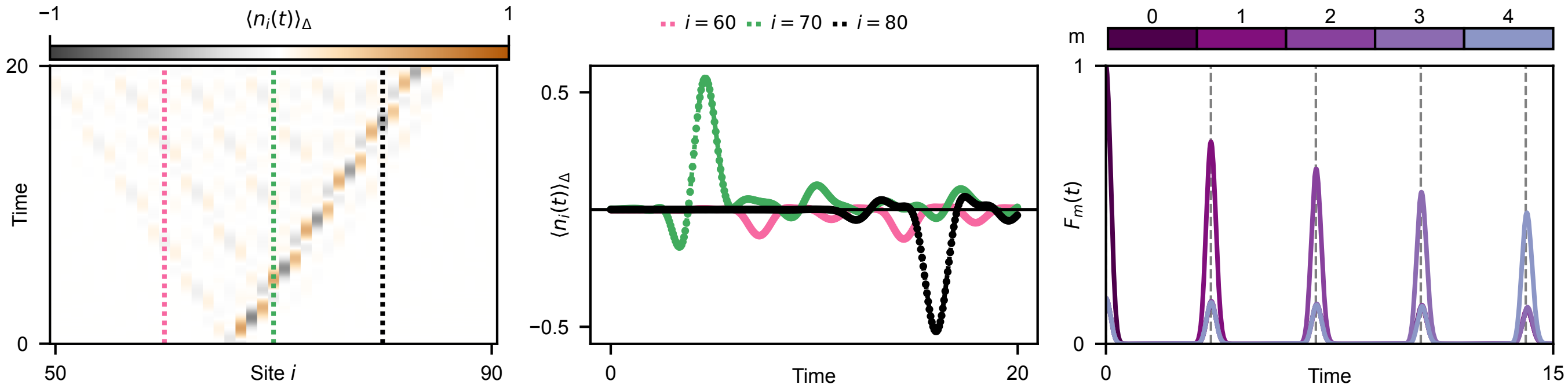

Supplementary Fig. 11. Dynamics of numerically optimized soliton state in the full quantum model defined on 150 sites. Evolving the MPS given by the scarred background cells with the angles $\theta^S_{1,2,3} = (1.05977133, 1.46820419, 0.0000131027276)$ and the soliton angles given by $\theta^R_{1,2,3} = (0.990853599, 0.454003269, 3.10125177)$ and positioned on $\{82, 83, 84\}$ leads to coherent propagation of the soliton. This manifests in the difference between the number operator of the state with and without a defect cell, as well as high translation fidelity peaks occurring at multiples of the revival period $T \approx 3.52$.

In Ref. [9], TDVP equations for the PXP model with an arbitrary unit cell size are also derived. For spin-1/2, the Ansatz used in that work is identical to ours, except for a factor two in the definition of the θ_j angle and a minus sign for the ϕ_j angles.

Using the results of that work with $\Delta_i = 0$ and $\Omega_i = 1$ for all i, $J = 1/2$, one gets the following results:

$$\partial_t \theta_i = 2\frac{\tan(\phi_i)}{\sin(\theta_i)}\tilde{h}_{s^x_i} + \frac{\eta_{i-1}\tan(\phi_{i-1})\tan\left(\frac{\theta_i}{2}\right)}{\eta_i}\tilde{h}_{s^x_{i-1}} \tag{16}$$

$$\partial_t \phi_i = \frac{\tilde{h}_{s^x_i}}{\sin^2\left(\frac{\theta_i}{2}\right)} - 2\frac{\tan\left(\frac{\theta_{i+1}}{2}\right)}{\eta_{i+1}}\tilde{R}_{\theta_{i+1}} - \frac{4}{\eta_i \sin(\theta_i)}\tilde{R}_{\theta_i}\ , \tag{17}$$

where

$$\tilde{h}_{s^x_i} = \sin(\theta_i)\cos(\phi_i)\cos\left(\frac{\theta_{i+1}}{2}\right) \tag{18}$$

$$\tilde{R}_{\theta_i} \equiv \frac{1}{4}\eta_{i-1}\sin\left(\frac{\theta_i}{2}\right)\sin(\theta_{i-1})\cos(\phi_{i-1}) + \frac{\eta_i}{2}\cos(\phi_i)\cos\left(\frac{\theta_{i+1}}{2}\right). \tag{19}$$

As noted in Ref. [9] below Eq. (20), for spin-1/2 all $\tilde{c}_i = 0$.

The η_i in these equations can be linked to the $\langle n_i \rangle$ we define in Eq. (15) of the main text as $\langle n_i \rangle = \sin^2\left(\frac{\theta_i}{2}\right)\eta_i$. This can be seen clearly by rewriting the η_i given for small values of L in Eq. (10) of Ref. [9] in a familiar form as

$$\eta_i = \begin{cases} \frac{1}{1-\sin^2\left(\frac{\theta_i}{2}\right)} & L = 1 \\ \frac{1-\sin^2\left(\frac{\theta_{i-1}}{2}\right)}{1-\left[-\sin^2\left(\frac{\theta_{i-1}}{2}\right)\right]\left[-\sin^2\left(\frac{\theta_i}{2}\right)\right]} & L = 2 \\ \frac{1-\sin^2\left(\frac{\theta_{i-2}}{2}\right)\left[1-\sin^2\left(\frac{\theta_{i-1}}{2}\right)\right]}{1-\left[-\sin^2\left(\frac{\theta_{i-2}}{2}\right)\right]\left[-\sin^2\left(\frac{\theta_{i-1}}{2}\right)\right]\left[-\sin^2\left(\frac{\theta_i}{2}\right)\right]} & L = 3\ . \end{cases} \tag{20}$$

Substituting the full expressions for η_i and $\tilde{h}_{s^x_i}$, we can further simplify Eq. (16) as

$$\frac{\partial_t \theta_i}{2} = \sin(\phi_i)\cos\left(\frac{\theta_{i+1}}{2}\right) + \frac{\langle n_{i-1}\rangle}{\langle n_i \rangle}\sin(\phi_{i-1})\sin\left(\frac{\theta_{i-1}}{2}\right)\cot\left(\frac{\theta_{i-1}}{2}\right)\sin^3\left(\frac{\theta_i}{2}\right). \tag{21}$$

This matches exactly with our independently derived Eq. (13) of the main text up to the transformations $\theta_i/2 \to \theta_i$ and $\phi_i \to -\phi_i$ which stems from the difference in the Ansätze used. We can perform the exact same procedure on

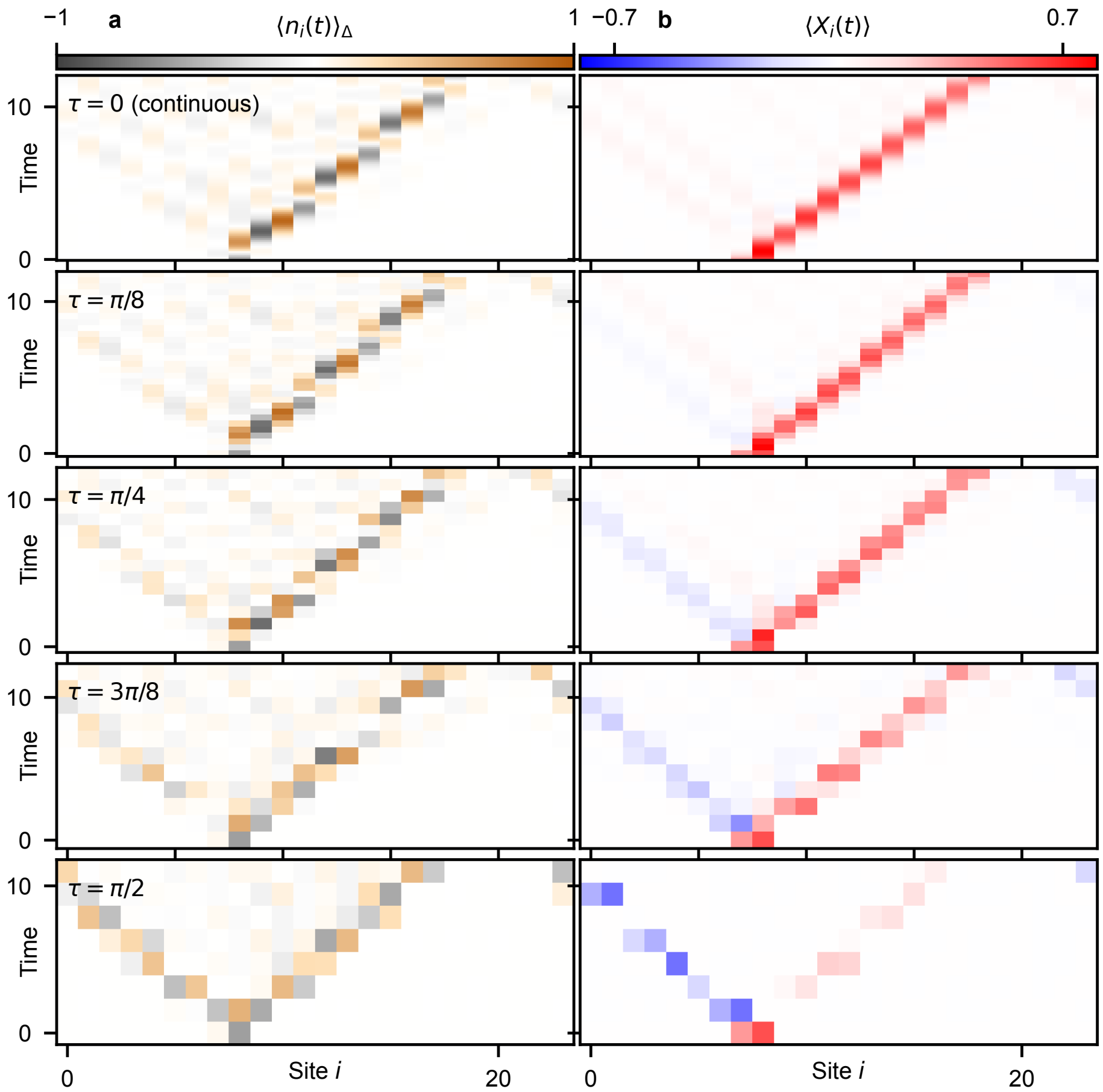

Supplementary Fig. 12. Time evolution of solitons under the Trotterized PXP Hamiltonian given in Eq. (23) with step sizes interpolated between the time-continuous PXP model ($\tau = 0$) up to the integrable point ($\tau = \pi/2$) **a,** The number operator density difference of the initial $|S\rangle^{\otimes 2}\,|R\rangle\,|S\rangle^{\otimes 5}$ state reveals coherent propagation for evolution with all considered Trotter step sizes, but the defect loses its chirality upon increasing τ and spreads nearly symmetrically in both directions for $\tau = \pi/2$. **b,** The energy-soliton state $|S\rangle^{\otimes 2}|R^{+}\rangle|S\rangle^{\otimes 5}$ also shows an increasingly symmetric propagation of $\langle X_i(t)\rangle$ (with opposite signs) with increasing Trotter step size.

Eq. (17). While this requires substantially more steps, it is straightforward and leads to

$$
\begin{aligned}
-\partial_t \phi_i = \cos(\phi_i) &\left[\sin^3\left(\frac{\theta_{i+1}}{2}\right)\frac{\langle n_i\rangle \cot\left(\frac{\theta_i}{2}\right)}{\langle n_{i+1}\rangle \cot\left(\frac{\theta_{i+1}}{2}\right)} - 2\cos\left(\frac{\theta_{i+1}}{2}\right)\cot(\theta_i)\right] \\
&+ \cos\left(\frac{\theta_{i+2}}{2}\right)\tan\left(\frac{\theta_{i+1}}{2}\right)\cos(\phi_{i+1}) + \sin\left(\frac{\theta_i}{2}\right)\frac{\langle n_{i-1}\rangle \cot\left(\frac{\theta_{i-1}}{2}\right)}{\langle n_i\rangle \cot\left(\frac{\theta_i}{2}\right)}\cos(\phi_{i-1}),
\end{aligned}
\tag{22}
$$

which now matches with Eq. (15) of the main text up to the same transformations $\theta_i/2 \to \theta_i$ and $\phi_i \to -\phi_i$.

Supplementary Note 5. FLOQUET DYNAMICS

Recent works have investigated Floquet realizations of the Rydberg atom chain, with special attention to classical cellular automaton (CA) limits, which revealed surprising connections to quantum many-body dynamics [10–12]. Especially interesting to us in this context is Ref. [10], where the authors analyzed the RCA201/Floquet-PXP model, which is an integrable classical CA. Interestingly, this model exhibits solitons that show ballistic transport and interact non-trivially upon collisions with a phase shift but otherwise re-emerge intact after the collision. They can

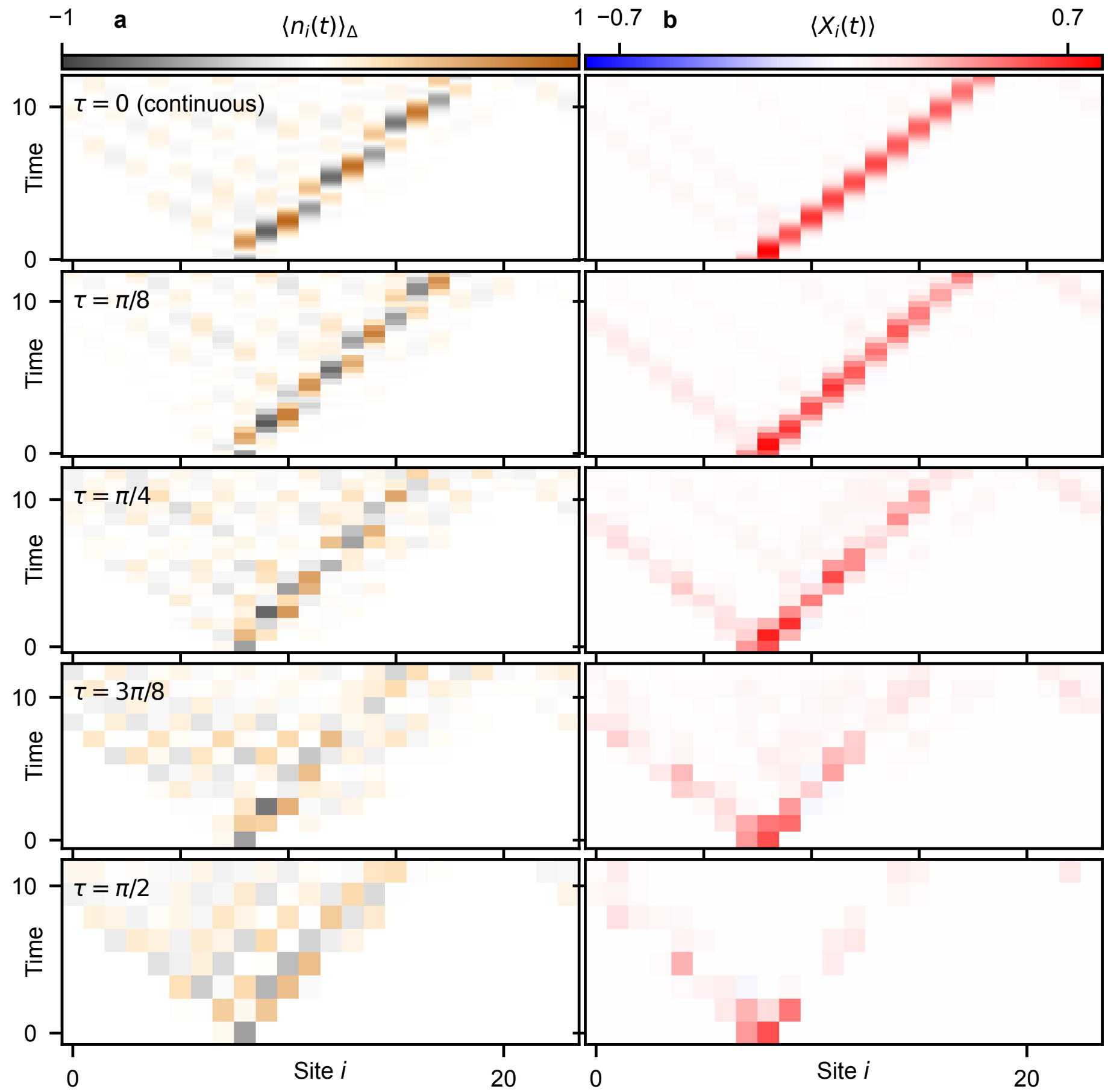

Supplementary Fig. 13. Dynamics of solitons under evolution of the Trotterized PXP Hamiltonian given in Eq. (24) with step sizes interpolated between the time-continuous PXP model ($\tau = 0$) up to the integrable point ($\tau = \pi/2$) **a,** The number operator density difference of the initial $|S\rangle^{\otimes 2}|R\rangle|S\rangle^{\otimes 5}$ shows less coherent propagation for larger step sizes but still appears to move predominantly to right up to $\tau = \pi/2$. **b,** The soliton dynamics shown in form of the expectation value $\langle X_i(t)\rangle$ of the state $|S\rangle^{\otimes 2}|R^+\rangle|S\rangle^{\otimes 5}$ becomes increasingly incoherent for larger Trotter step sizes.

be understood as domain walls between three vacuum states. These solitons in the classical model bear resemblance in certain aspects to the quasi-solitons that we study in the time-continuous PXP model.

The CA point can be obtained by a Trotter discretization of the time-continuous PXP dynamics. Concretely, one splits the PXP Hamiltonian $H = \sum_j P_{j-1}X_jP_{j+1}$ into even- and odd-site parts, $H = H_e + H_o$, with $H_{e(o)} = \sum_{j\,\text{even(odd)}} P_{j-1}X_jP_{j+1}$, and defines the Floquet operator:

$$U_\tau \;=\; U_\tau^e U_\tau^o \;=\; e^{-iH_e\tau}\, e^{-iH_o\tau}, \tag{23}$$

where τ is the Trotter step size. For $\tau \to 0$ one recovers the time-continuous evolution, while $\tau = \pi/2$ yields the integrable classical CA that maps classical states to classical states. A priori, there is no reason why the soliton dynamics of the CA should leave an imprint in the time-continuous PXP model, since $\tau = \pi/2$ is far beyond the perturbative regime. However, in Ref. [12] the authors found surprising connections between the quantum many-body scars associated with the $|\mathbb{Z}_2\rangle$ state and some eigenstates of the PXP CA. This connection was established by treating τ as an interpolation parameter and demonstrating an adiabatic connection between eigenstates of the time-continuous PXP model and eigenstates of the CA, at least for QMBSs close to zero energy. Furthermore, Ref. [12] studied initial product states that host solitons under the CA time evolution and tracked their dynamics as a function of τ, finding that certain signatures of the soliton dynamics persist down to small Trotter steps.

One can ask how the quasi-solitons found in our work change when interpolating between the time-continuous PXP model and the cellular automaton point by varying the Trotter step size. However, the initial states at play here are not product states but weakly entangled states, which complicates matters. To test whether the dynamics of

these soliton states in the time-continuous model are connected to the solitons in the CA, one would, in principle, have to interpolate the Trotter step while at the same time deforming the entangled soliton state into a CA soliton product state. It is not clear how this should be done: this is not a simple one-dimensional interpolation, but rather an arbitrary trajectory in the exponentially large Hilbert space that starts at $\tau = 0$ from the soliton state in the time-continuous PXP model and ends at $\tau = \pi/2$ at a CA soliton product state. As already mentioned, there is no physical argument for why these should be related, since the change in the Trotter step size is far beyond the perturbative regime. Potential explorations in this direction are beyond the scope of this work and are left for future research. Here we only take a first step and explore how the dynamics of our states with a single soliton $|R\rangle$ and $|R^+\rangle$ embedded in the scarred background change when interpolating from the time-continuous limit $\tau = 0$ to the integrable point at $\tau = \pi/2$ while leaving the state invariant. This exploration is also relevant for potential implementations on a quantum computer. In Supplementary Fig. 12 we see that the chiral dynamics of the soliton persists up to large Trotter steps ($\tau = \pi/4$); while the dynamics remains coherent up to $\tau = \pi/2$, it becomes increasingly less chiral, and the defect propagates in both directions.

It is also not clear whether the Trotterization performed in [12] is the best choice for uncovering a connection between our solitons in the time-continuous model and the cellular automaton. In that work, this Trotterization was used to establish a connection between the scarring of the $|\mathbb{Z}_2\rangle$ state and the CA. However, the Trotterization is not unique, and it could be that other decompositions of the Hamiltonian establish a stronger connection to our solitons in the time-continuous PXP model. Another possible way of decomposing the Hamiltonian would be $H = H_1 + H_2 + H_3$ with $H_r = \sum_{j \bmod 3 = r-1} P_{j-1} X_j P_{j+1}$ $r \in \{1, 2, 3\}$, leading to the Floquet operator:

$$U_\tau^{(3)} = e^{-iH_3\tau}\, e^{-iH_2\tau}\, e^{-iH_1\tau}, \tag{24}$$

for a time step τ. Likewise, this becomes a cellular automaton at $\tau = \pi/2$. Starting from the fully polarized down state $|0\rangle = |{\downarrow\downarrow\downarrow}\cdots\rangle$, the evolution exhibits a simple period-4 orbit that cycles through translated $|\mathbb{Z}_3\rangle = |{\downarrow\downarrow\uparrow\downarrow\downarrow\uparrow}\ldots\rangle$ configurations $|0\rangle \xrightarrow{U^{(3)}_{\pi/2}} T\,|\mathbb{Z}_3\rangle \xrightarrow{U^{(3)}_{\pi/2}} T^2\,|\mathbb{Z}_3\rangle \xrightarrow{U^{(3)}_{\pi/2}} |\mathbb{Z}_3\rangle \xrightarrow{U^{(3)}_{\pi/2}} |0\rangle$. However, we have not explored potential quasi-particle dynamics in this CA version. Instead, we again compare a simple interpolation in Trotter step size between the time-continuous PXP model and the $\tau = \pi/2$ point, analogous to the even/odd Trotterization above, for the solitons $|R\rangle$ and $|R^+\rangle$ in Supplementary Fig. 13. It seems that the defect propagation in both cases becomes less coherent for increasing step sizes τ while $|R\rangle$ remains somewhat chiral up to $\tau = \pi/2$.

In summary, the investigation of two different Trotterizations shows that the even/odd scheme has coherently propagating, however non-chiral defects related to our soliton initial state, whereas the period-3 Trotterization leads to decoherence of solitons. We note that a more thorough investigation would be required to establish any potential meaningful connections between our solitons in the time-continuous PXP model and solitons in Floquet realizations of the PXP model.

Supplementary Note 6. REALIZATION OF SOLITONS IN RYDBERG ATOM ARRAYS

All results so far have been obtained for the idealized PXP Hamiltonian in Eq. (1) of the main text, which approximates the Rydberg chain in the regime of strong nearest-neighbor interactions and weak residual interactions beyond. Under realistic conditions, however, the Rydberg blockade is not perfect, and van der Waals interactions extend beyond nearest neighbors. To examine the stability of the solitons in a more realistic scenario, we simulate their dynamics under the full two-level Rydberg Hamiltonian of the form:

$$\frac{H}{\hbar} = \frac{\Omega}{2}\sum_i X_i - \Delta\sum_i n_i + \sum_{i<j} V_{ij}\, n_i n_j, \qquad V_{ij} = \frac{V_1}{|i-j|^6}. \tag{25}$$

Physical parameters of this Hamiltonian correspond to the Rabi frequency Ω, detuning strength Δ, and repulsive van der Waals interactions V_{ij} between two Rydberg excitations. Our numerical simulations below consider parameters $\Omega = 2 \times 2\pi$ MHz, $V_1 = 14 \times 2\pi$ MHz $= 7\Omega$, and $\Delta = 0.017 \times 2\pi$ MHz $= 0.0085\Omega$. As proposed in Ref. [13], the non-zero value of the detuning Δ serves to mitigate the effects of long-range interactions.

We compare the time evolution generated by the PXP Hamiltonian and by Eq. (25) for a single right-moving soliton $|R\rangle$, as well as for an energy-carrying soliton $|R^+\rangle$ in terms of local experimentally observable quantities such as the number operator density and kinetic energy density defined as $\langle X_i^k(t)\rangle = \langle\psi(t)|X_i|\psi(t)\rangle$, as well as the translation fidelity to get a better understanding of their decay. Note that we omit projectors in the definition of the kinetic energy density since the Rydberg blockade can be violated under time evolution generated by Eq. (25). Moreover, note that $\sum_i X_i^k$ is not a conserved quantity for the full Rydberg Hamiltonian.

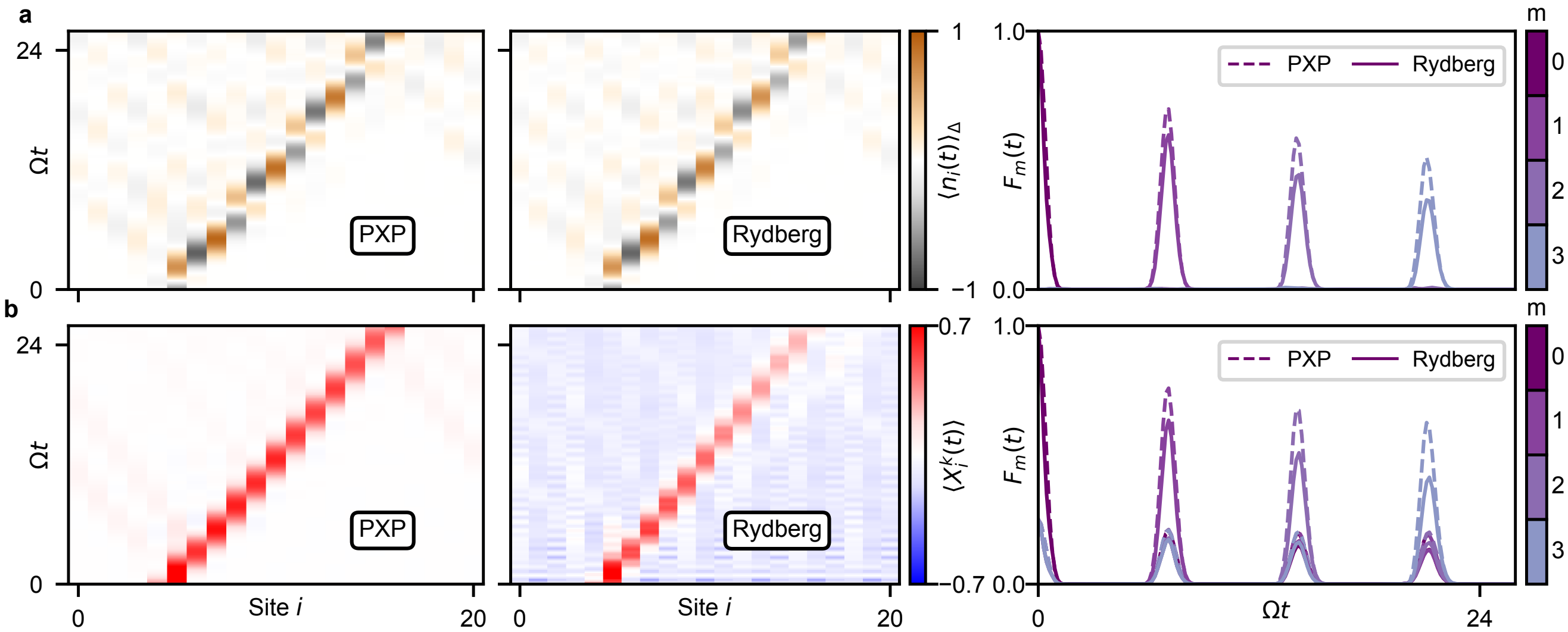

Supplementary Fig. 14. Comparison of unitary time-evolution of solitonic states under the full Rydberg Hamiltonian given in Eq. (25) and the PXP Hamiltonian given in Eq. (1) of the main text performed with sparse Krylov methods. **a,** The number operator density difference of the initial state $|S\rangle|R\rangle\,|S\rangle^{\otimes 5}$ evolved under the PXP Hamiltonian (left) and the full Rydberg Hamiltonian (middle) shows in both cases coherent propagation of the solitonic defect. The translation fidelity revivals (right) for the full Rydberg Hamiltonian are slightly smaller than for the PXP model. **b,** The kinetic energy density of the initial state $|S\rangle|R^{+}\rangle\,|S\rangle^{\otimes 5}$ evolved under the PXP Hamiltonian (left) and the full Rydberg Hamiltonian (middle) show coherent dynamics of the solitonic defect with a somewhat faster decay under evolution of the Rydberg Hamiltonian. The translation fidelity revivals (right) for the full Rydberg Hamiltonian are also slightly smaller.

In Supplementary Fig. 14, it can be seen that the number operator density between the PXP Hamiltonian and the full Rydberg Hamiltonian seems visually almost identical. The energy density $\langle X_i(t)\rangle$ shows a slightly faster decay under time evolution of the full Rydberg Hamiltonian. Furthermore, we can see that even the translation fidelities for the solitons under the time evolution with the full Rydberg Hamiltonian are comparable to those of the PXP model. These results demonstrate that although Rydberg arrays only approximately realize the PXP model, the dynamics of solitons is only mildly affected by these imperfections. Therefore, the solitons presented in our work can be implemented and studied experimentally in Rydberg atom arrays.

Supplementary Note 7. SOLITONS IN THE PPXPP MODEL

It was shown that generalizations of the PXP model with larger blockade radii host scarred states as well [2]. Here, we focus on the specific generalization of PXP to blockade of radius two, denoted as the PPXPP model:

$$H = \sum_{i=1}^{N} P_{i-2}P_{i-1}X_iP_{i+1}P_{i+2}. \tag{26}$$

This model requires $|\uparrow\rangle$ spin states to be separated by at least two sites, and can be approximately realized by a Rydberg atom array in a triangular ladder geometry [2]. In our previous work, we demonstrated that this model hosts at least two scarred states with unit cell sizes of 4, 5, and 6. Notably, we do not find the soliton excitations on top of scarred state with unit cell sizes 4 and 6, suggesting that the size of the unit cell has to be *odd* as a prerequisite for soliton existence.

For the 5-sites unit cell, we consider the following scarred initial state:

$$|K_5\rangle = \bigotimes_{k=1}^{N/5} |S_5\rangle\,, \qquad |S_5\rangle = \frac{1}{\sqrt{\beta^2+2}}|{\downarrow\downarrow}\rangle(\beta|{\uparrow\downarrow\downarrow}\rangle + |{\downarrow\uparrow\downarrow}\rangle + |{\downarrow\downarrow\uparrow}\rangle), \tag{27}$$

and search for defect cells of 5 sites that would feature coherent right-propagation when initialized in the background of $|S_5\rangle$ cells. In contrast to the PXP solitons, which can be approximately understood as translated unit cells of

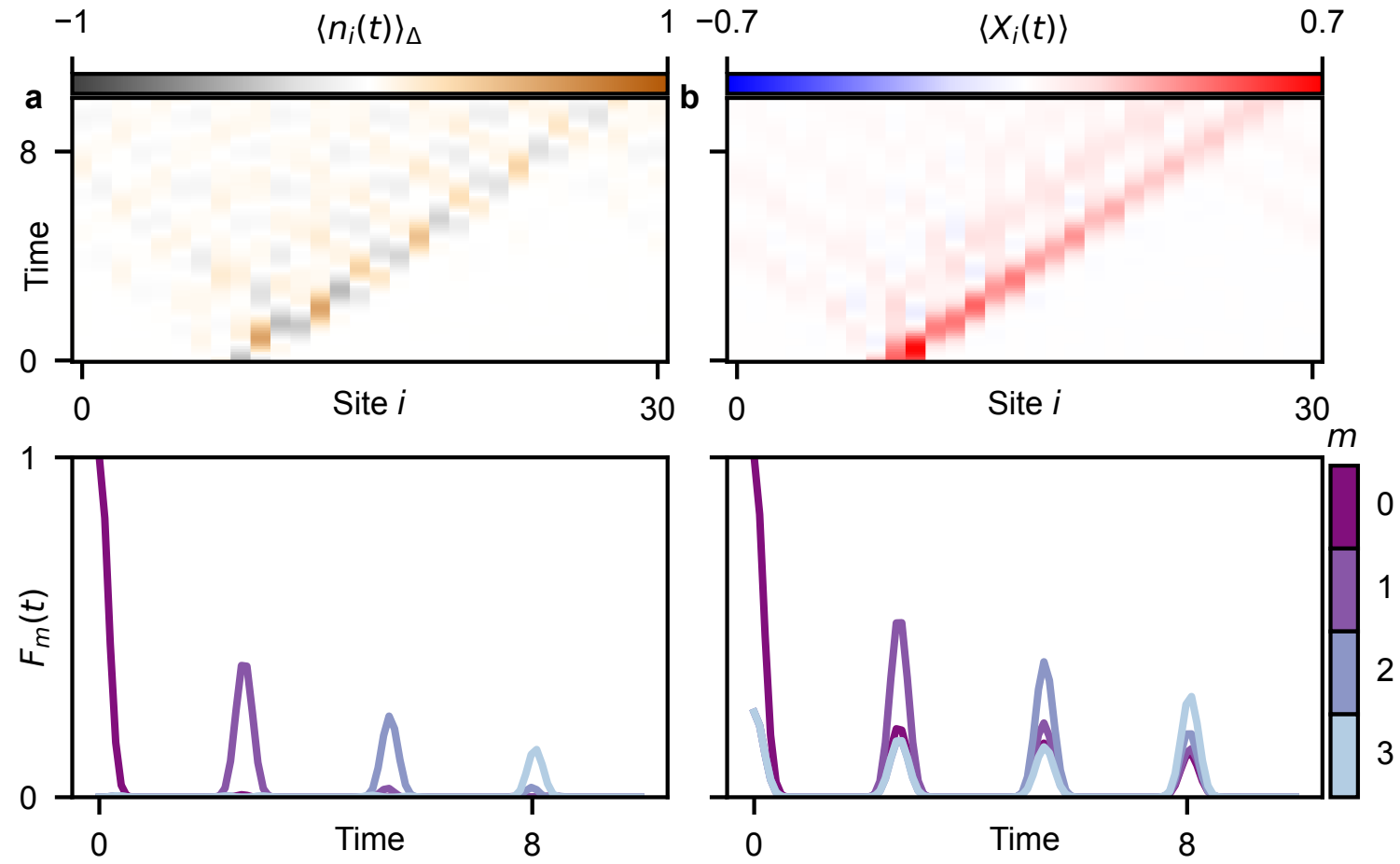

Supplementary Fig. 15. Unitary time-evolution of a system with 30 sites with periodic boundary conditions under the PPXPP Hamiltonian produced using Krylov time evolution. The states have a scarred background with the 5-site unit cell $|S_5\rangle$ and the defect cell for both states is positioned at the sites $\{6,7,8,9,10\}$. **a,** The zero-energy defect cell $|R_5\rangle$ initially shows coherent propagation to the right but distorts the scarred background more and also decays faster compared to the solitons in the PXP model. Also, the translation fidelity peaks show less pronounced revivals and decay faster **b,** Similar behavior can be observed for the energy-carrying soliton $|R_5^+\rangle$.

scarred initial state at quarter and three-quarter periods, a similar intuition does not work for the PPXPP model. Indeed, we observe that appropriately shifted unit cells from the state $|K_5(T/4)\rangle$ and $|K_5(3T/4)\rangle$ violate the blockade condition and cannot be inserted into the background of $|S_5\rangle$ cells.

Hence, we search for the soliton cells by imposing similar constraints on the potential left- and right-moving defect cells as in the PXP model: (i) the first two sites have to be in the ground state to avoid blockade violation, (ii) $\{|S_5\rangle, |R_5\rangle, |L_5\rangle\}$ are orthonormal to each other and (iii) they satisfy the conjugation condition under chiral symmetry, $|L_5\rangle = \mathcal{C}|R_5\rangle$. We optimize over the family of states that satisfy these conditions to maximize the translation fidelity of a single right-moving defect $|R_5\rangle$ after one revival period. Numerical optimization yields the following cells:

$$|L_5, R_5\rangle = \frac{1}{c}|{\downarrow\downarrow}\rangle(\mp 1.133|{\downarrow\downarrow\downarrow}\rangle + 0.84i|{\uparrow\downarrow\downarrow}\rangle + 0.10i|{\downarrow\uparrow\downarrow}\rangle - 0.646i|{\downarrow\downarrow\uparrow}\rangle), \tag{28}$$

with c being a normalization constant. These cells, taken in superposition with $|S_5\rangle$ in the form $|R_5^+\rangle = (|S_5\rangle + |R_5\rangle)/\sqrt{2}$, can also be used to initialize right- (and analogously left-) moving energy solitons.

We numerically check the unitary dynamics of such optimized cells now in a larger system. Specifically, inserting $|R_5\rangle$ in the scarred state $|K_5\rangle$ yields a right-moving defect as can be seen in Supplementary Fig. 15**a**. There, we show, similarly to the main text, the difference in expectation value of the local number operator between dynamics with and without a soliton defect. It is visible that although the propagation of disturbance is highly asymmetric between left and right directions, the pattern becomes washed out faster. Accordingly, the translation fidelity peaks are less pronounced compared to the PXP model. Finally, in Supplementary Fig. 15**b** we also see that the energy soliton $|R^+\rangle$ defined above shows somewhat more stable coherent propagation.

In conclusion, the results shown in this section suggest that solitons are not limited to the PXP model. This opens an exciting direction for searching for coherently propagating excitations in other models, with PPPXPPP and longer-range blockade generalizations of PXP being one perspective family.

SUPPLEMENTARY REFERENCES

[1] Turner, C. J., Michailidis, A. A., Abanin, D. A., Serbyn, M. & Papić, Z. Weak ergodicity breaking from quantum many-body scars. *Nature Physics* **14**, 745–749 (2018).

[2] Kerschbaumer, A., Ljubotina, M., Serbyn, M. & Desaules, J.-Y. Quantum many-body scars beyond the PXP model in Rydberg simulators. *Physical Review Letters* **134**, 160401 (2025).

[3] Serbyn, M., Abanin, D. A. & Papić, Z. Quantum many-body scars and weak breaking of ergodicity. *Nature Physics* **17**, 675–685 (2021).

[4] Chandran, A., Iadecola, T., Khemani, V. & Moessner, R. Quantum many-body scars: A quasiparticle perspective. *Annual Review of Condensed Matter Physics* **14**, 443–469 (2023).

[5] Moudgalya, S., Bernevig, B. A. & Regnault, N. Quantum many-body scars and Hilbert space fragmentation: A review of exact results. *Reports on Progress in Physics* **85**, 086501 (2022).

[6] Virtanen, P. *et al.* SciPy 1.0: Fundamental Algorithms for Scientific Computing in Python. *Nature Methods* **17**, 261–272 (2020).

[7] Storn, R. & Price, K. Differential Evolution – A Simple and Efficient Heuristic for global Optimization over Continuous Spaces. *Journal of Global Optimization* **11**, 341–359 (1997). Publisher: Springer Science and Business Media LLC.

[8] Michailidis, A. A., Turner, C. J., Papić, Z., Abanin, D. A. & Serbyn, M. Slow quantum thermalization and many-body revivals from mixed phase space. *Physical Review X* **10**, 011055 (2020).

[9] Hu, Z. & Wu, B. Variational method for $\mathbb{Z}_K$ wavefunctions in spin-J PXP model. *arXiv ePrints* (2025). 2501.09301.

[10] Wilkinson, J. W. P., Klobas, K., Prosen, T. & Garrahan, J. P. Exact solution of the Floquet-PXP cellular automaton. *Physical Review E* **102**, 062107 (2020).

[11] Iadecola, T. & Vijay, S. Nonergodic quantum dynamics from deformations of classical cellular automata. *Physical Review B* **102**, 180302 (2020).

[12] Giudici, G., Surace, F. M. & Pichler, H. Unraveling PXP many-body scars through Floquet dynamics. *Physical Review Letters* **133**, 190404 (2024).

[13] Bluvstein, D. *et al.* Controlling quantum many-body dynamics in driven Rydberg atom arrays. *Science* **371**, 1355–1359 (2021).
