# Supplementary material for: Quasi-solitons in Rydberg atom chains
Source: Nat Commun. 2026 Jul 17;17:8783. doi: 10.1038/s41467-026-75598-1 (PMC13493837; doi:10.1038/s41467-026-75598-1)
Supplement: Supplementary file 2 [file 41467_2026_75598_MOESM2_ESM.pdf]

nature portfolio

Peer Review File

Quasi-solitons in Rydberg atom chains

Corresponding Author: Mr Aron Kerschbaumer

This file contains all reviewer reports in order by version, followed by all author rebuttals in order by version.

Version 0:

Reviewer comments:

Reviewer #1

(Remarks to the Author)
In “Discrete solitons in Rydberg atom chains”, Kerschbaumer, Desaules, Ljubotina and Serbyn discover soliton-like behavior in the PXP model in Rydberg atom arrays, which has received significant recent attention owing to its unusual non-equilibrium dynamics.
They find solitonic local excitations on top of a certain initial state which is periodic over three sites: these excitations propagate nearly ballistically over space and time. This is evident in local observables such as the Rydberg excitation density and the energy density, as well as non-local observables such as the many-body fidelity of states that are translated in space. Entangled pairs of solitons can propagate and carry entanglement. Finally, the authors give a variational description of this dynamics in terms of the time-dependent variational principle (TDVP), which postulates that the state evolves within a manifold parameterized by a few parameters. Remarkably, the TDVP parameters follow simple equations of motion which appear to be able to account for the observed solitonic dynamics.
This strongly defies the standard expectations of chaotic many-body dynamics, and constitutes one of the most surprising phenomena observed in the PXP model known to date. While this discovery arises primarily from numerical simulations, the authors have a plausible but preliminary theory for this phenomenon in terms of the TDVP.
We believe that this finding is significant and deserves publication in a journal of high standing such as Nature Communications. However, we highlight certain aspects of this work which could be improved:
1. The connection between this phenomenon and many-body scarring (as well as other phenomena in the PXP model) could be clarified. For example, a natural question is whether the soliton states as well as the background state |K> have high overlap with many-body scars. This is not discussed in the main text but is discussed briefly in the supplemental material, specifically Fig. S8. There, they show that these states have high overlap with an exceptional set of eigenstates, but do not discuss this further. Basic facts such as that whether these are the Z2 or Z3 scar eigenstates are not addressed. I infer that they are not the Z2 scar eigenstates, based on the entanglement entropy plot, but this may not be obvious to many readers (As a technical comment, the symmetry quantum number in Fig. S8 should be indicated).
There are connections to other known phenomena in the PXP model that could be addressed, such as to the ballistic behavior seen in Surace et. al. (Ref. [25]) and Giudici et. al. (Ref. [33]), but the scar phenomenology will likely be what most readers will find lacking.
2. Similarly, more discussion of the TDVP ansatz could be useful. How does this ansatz differ from that of Ref. [23,24]? If the manifolds share a common phase space, how do the trajectories of the Z3 state and the |K> state compare?
As a scientific question, is there a description of the limit-cycle behavior in Fig. 4b in terms of the equation of motion Eq. (9)? Is it a particular solution to Eq. (9) and if so, are there non-trivial phase relations for the angle parameters between neighboring sites? This would seem to be the case from Fig. 4c, which indicates that there are multiple limit cycles related by a phase shift. For the |K> state (without the solitons), it is a system of three coupled equations which presumably can be studied. Some discussion of the “leakage” out of the TDVP manifold may also be useful: as established in previous works, only special trajectories of the TDVP manifold experience small leakage which justifies their applicability.
These basic and natural questions do not seem to be addressed in the paper, which makes the work seem somewhat incomplete.
We believe that clarifying these points will strengthen the paper. Given the discoveries made in this paper and the otherwise thorough analysis, we believe it should be eventually published in Nature Communications.

Reviewer #2

(Remarks to the Author)
I co-reviewed this manuscript with one of the reviewers who provided the listed reports. This is part of the Nature

Communications initiative to facilitate training in peer review and to provide appropriate recognition for Early Career Researchers who co-review manuscripts.

Reviewer #3

(Remarks to the Author)
This manuscript investigates the emergence of soliton-like excitations in one-dimensional Rydberg atom chains described by the PXP model. The authors identify localized, directionally propagating defects embedded in special scarred background states, demonstrate their long-lived coherent motion, and show that such excitations can carry both energy and quantum information. The work combines extensive numerical simulations with a phenomenological construction of soliton states and a variational analysis based on the time-dependent variational principle (TDVP), revealing related soliton-like trajectories in an associated classical nonlinear dynamical system.

The existence of chiral, stable quasiparticles in PXP-type models is, however, already well established in the literature, particularly in the context of Floquet realizations such as the PXP cellular automaton, where ballistic propagation, absence of dispersion, and characteristic scattering properties have been studied in depth [PRE 102, 062107]. Within this broader context, the present manuscript makes a meaningful contribution by identifying an interesting and distinct class of soliton-like excitations directly in the time-continuous Hamiltonian, without relying on exact Floquet dynamics. The additional connection drawn to classical TDVP trajectories is appealing and represents one of the more original aspects of the paper.

Nevertheless, much of the phenomenology reported here closely parallels what is already known from Floquet-PXP models. The solitons presented share several defining features with previously studied chiral quasiparticles, including fixed-velocity propagation, the absence of dispersion, and the lack of a nontrivial scattering phase. Similarly, the idea of exploiting such chiral excitations for quantum information transport has already been put forward in the Floquet-PXP setting, and prior works have developed these ideas to the point of proposing universal quantum computing schemes based on such dynamics [PRL 131, 170601]. From this perspective, the information-transport protocols presented in the manuscript appear conceptually very close to existing proposals, differing mainly in their realization within the time-continuous PXP model. While the authors emphasize experimental relevance, the manuscript does not explicitly investigate the stability of the proposed solitons under more realistic Rydberg Hamiltonians beyond the PXP approximation, making it difficult to assess their practical observability.

In summary, this is a technically solid and clearly written manuscript that presents interesting results and deserves publication in some form. However, given the substantial overlap in phenomenology with earlier work on Floquet-PXP models and chiral quasiparticles, I am not fully convinced that the level of conceptual novelty meets the standards expected for publication in Nature Communications.

Reviewer #4

(Remarks to the Author)
This is a very interesting work in the timely researches of PXP model. It presents a new scarred state similar with the Z3 state. Two so-called solitons L and R are introduced which are found to unidirectionally move to the left and right hand side, respectively, with long-standing quantum coherence. As one normally considers the empirical Z2, Z3 and Z4 states as the scars, this idea is really appealing and exotic. Basically, I'll be willing to recommend publishing, but there are still several issues to be clarified.

1, Not every particles that move unidirectionally can be called soliton. For example, the fracton also has got this feature. A formal definition of soliton should be a stable solution of a nonlinear equation. Although there is an Eq. 9, it has not exact solution up to date. Instead, the authors may consider domain wall, exciplex, or something like that.

2, The time evolution should be calculated to longer duration to exhibit the complete decay of the excitations, such that one can estimate the lifetime.

3, Using different colors to denote S, L and R state is not clear. As the right two sites are in XY plane in S state, the authors may consider to use two horizontal arrows for S. And those for L and R could be up-down symmetric.

4, Zi has not got definition, though it is obvious for experts.

5, Why does the collision change the energy without phase shift, considering the system is closed?

6, How to map theta back to the quantum state? Some superposition coefficients?

Version 1:

Reviewer comments:

Reviewer #1

(Remarks to the Author)
The authors have addressed our previous comments and questions: they have clarified their TDVP methods and have

discussed the connections to known phenomena such as many-body scarring and other solitonic behavior in the PXP model and its variants.
Minor comments:
1. In the added phrase “with Zi = |↑⟩ ⟨↑| – |↓⟩ ⟨↓|” below Eq (2), they should indicate that this operator acts on site i.
2. The authors claim that there is a comment on the relation between the |K> and |Z3> states in the “Model and properties” section of the main text and in S2B of the supplement. We cannot find such a comment that explicitly mentions the relation to the |Z3> state.

Once these comments are addressed, we believe that this work represents a significant new finding in the dynamics of a PXP model, a model that has received wide interest both in theory and experiment, and merits publication in Nature Communications.

Reviewer #2

(Remarks to the Author)
I co-reviewed this manuscript with one of the reviewers who provided the listed reports. This is part of the Nature Communications initiative to facilitate training in peer review and to provide appropriate recognition for Early Career Researchers who co-review manuscripts.

Reviewer #3

(Remarks to the Author)
I thank the authors for their detailed response and for the efforts made to improve the manuscript. In particular, I appreciate the added discussion of the relation to Floquet-PXP models (which, however, remains largely speculative) and the inclusion of additional references and numerical results. These additions certainly strengthen the presentation and help place the work in a broader context.

That said, my main concern regarding the level of conceptual novelty remains only partially addressed. The authors emphasize that “the central message of our work is the identification of coherently propagating defects in high-temperature states in a non-integrable, time-independent system.” However, the existence of coherently propagating defects in the non-integrable PXP model is, in my view, not new. In particular, related ballistic defect dynamics has already been observed in earlier works [25], where localized excitations propagate on top of scarred backgrounds. While those defects are not chiral in the static PXP model, they have subsequently been connected to chiral quasiparticles in integrable Floquet realizations such as the PXP cellular automaton [34].

From this perspective, the main conceptual advance of the present manuscript appears to be that the defects are already chiral directly in the time-continuous, non-integrable PXP Hamiltonian. While I agree that this is an interesting and nontrivial observation, I do not believe that it constitutes a sufficiently strong conceptual advance beyond the existing literature to meet the novelty standards expected for publication in Nature Communications. Rather, the work seems to establish that a phenomenology closely related to that of Floquet-PXP quasiparticles can already emerge in the static model, which I view as an important extension, but not necessarily a fundamentally new mechanism.

Reviewer #4

(Remarks to the Author)
The authors have addressed all my concerns in the revised version. I can then recommend the publication. By the way, I think the Reviewer 3 did not get the novel idea of this work, which is not about the dynamics but the new scarred states with localized and coherent deformation.

Open Access This Peer Review File is licensed under a Creative Commons Attribution 4.0 International License, which permits use, sharing, adaptation, distribution and reproduction in any medium or format, as long as you give appropriate credit to the original author(s) and the source, provide a link to the Creative Commons license, and indicate if changes were made.
In cases where reviewers are anonymous, credit should be given to 'Anonymous Referee' and the source.
The images or other third party material in this Peer Review File are included in the article's Creative Commons license, unless indicated otherwise in a credit line to the material. If material is not included in the article's Creative Commons license and your intended use is not permitted by statutory regulation or exceeds the permitted use, you will need to obtain permission directly from the copyright holder.
To view a copy of this license, visit https://creativecommons.org/licenses/by/4.0/

Response to Reviewer #1

We want to thank the Reviewer for the thorough study of our work and the useful comments that helped us to improve our manuscript and clarify its message.

The connection between this phenomenon and many-body scarring (as well as other phenomena in the PXP model) could be clarified. For example, a natural question is whether the soliton states as well as the background state $|K\rangle$ have high overlap with many-body scars. This is not discussed in the main text but is discussed briefly in the supplemental material, specifically Fig. S8. There, they show that these states have high overlap with an exceptional set of eigenstates, but do not discuss this further. Basic facts such as that whether these are the Z2 or Z3 scar eigenstates are not addressed. I infer that they are not the Z2 scar eigenstates, based on the entanglement entropy plot, but this may not be obvious to many readers (As a technical comment, the symmetry quantum number in Fig. S8 should be indicated).

We appreciate the detailed comment and fully agree with it. In order to clarify the roles of the different quantum many-body scars associated with the different scarred initial states in the PXP model, reported in Ref. [29], we now highlight them in Fig. S8. We have also specified the symmetry quantum numbers for which we show the eigenstates in that figure. Finally, we have expanded the discussion of the spectral decomposition of the soliton states in the "Discussion" section of the main text.

There are connections to other known phenomena in the PXP model that could be addressed, such as to the ballistic behavior seen in Surace et. al. (Ref. [25]) and Giudici et. al. (Ref. [33]), but the scar phenomenology will likely be what most readers will find lacking.

We have added a section in the supplement of the manuscript that discusses a potential connection between our quasi-solitons in the chaotic time-continuous PXP model to ballistic behavior in Floquet realizations of the PXP model, such as the cellular automaton discussed in Ref. [33](now [34] in current manuscript) and added a paragraph in the "Discussion" section of the main text.

Similarly, more discussion of the TDVP ansatz could be useful. How does this ansatz differ from that of Ref. [23,24]? If the manifolds share a common phase space, how do the trajectories of the Z3 state and the $|K\rangle$ state compare?

The on-site matrix of our MPS Ansatz for TDVP is identical to the spin 1/2 Ansatz in Ref. [23] and the Ansatz in Ref. [24] up to a different parametrization. Consequently, they span the same MPS manifold (we updated the description in the "Methods" section to clarify this relation). However, these papers consider only manifolds with a periodic 2- and 3-site unit cell and derive the corresponding equations of motion for them. We derive these equations of motion for *arbitrary* unit cell sizes, which enables us to study localized defects embedded in an otherwise homogeneous background. Specifically, using our equations of motion, we address the dynamics of a quasi-soliton embedded in a scarred background, as discussed in the section "Variational description" of the main text.

The relation between the $|K\rangle$ and $|Z_3\rangle$ states has been discussed in Ref. [29]. The $|K\rangle$ state lies essentially on the same periodic orbit as the 3-site reviving state discussed in Ref. [24]. $|Z_3\rangle$ has significant overlap with $|K\rangle$ for moderate system sizes and shows, therefore, relatively good revivals in the quantum model. The classical TDVP trajectory of $|Z_3\rangle$ stays near the periodic trajectory associated with $|K\rangle$, but it does not form a closed orbit; see Ref. [24] for details. Additionally, to the comment in the section "Model and properties" we have added a brief discussion in the supplement section S2.B. that clarifies the equivalence between these trajectories.

As a scientific question, is there a description of the limit-cycle behavior in Fig. 4b in terms of the equation of motion Eq.

(9)? Is it a particular solution to Eq. (9) and if so, are there non-trivial phase relations for the angle parameters between neighboring sites? This would seem to be the case from Fig. 4c, which indicates that there are multiple limit cycles related by a phase shift. For the $|K\rangle$ state (without the solitons), it is a system of three coupled equations which presumably can be studied.

We would like to emphasize that the quasi-soliton trajectory in the scarred background shown in Fig. 4 is not perfectly closed. It shows coherent soliton-like dynamics for long times, but eventually becomes chaotic. The apparent "cycles" in panel **b** are not closed. Furthermore, an analytic treatment of Eq. (9) is generally very challenging. Although the equations appear compact, for the system underlying Fig. 4, they amount to a set of 42 coupled nonlinear differential equations, which makes it difficult to extract simple phase relations between neighboring variables along the soliton trajectory. In order to provide more insights about the dynamics of the angles, we provided an additional figure in the supplement that shows the independent evolution of all angles, with and without the soliton.

We want to note that it is possible that a closed trajectory exists that realizes soliton-like dynamics similar to Fig. 4, that may open the possibility to some form of analytical treatment, but we were not able to find one. It could also be possible that finding a perfectly closed soliton-like trajectory requires an MPS Ansatz with a higher bond dimension. We are not aware of any analytic solution that describes the periodic scarred trajectory or a simple relation between angles of neighboring sites. No analytic solution has been reported even for the simpler $|Z_2\rangle$ scarred trajectory reported in Ref. [23].

Some discussion of the "leakage" out of the TDVP manifold may also be useful: as established in previous works, only special trajectories of the TDVP manifold experience small leakage which justifies their applicability.

The investigated 3-site scarred trajectory in Ref. [24], which is identical to ours, has been shown to have low leakage. Furthermore, we show in the supplement that the entanglement growth of the purely scarred state and the same state with a single solitonic defect is comparably slow. Therefore, we have sufficient evidence of the low-leakage nature of this trajectory, and believe that our TDVP description is justified. We thank the Reviewer for pointing out this omission and have added a corresponding discussion in the section "Variational description".

Response to Reviewer #3 We thank the Reviewer for their thorough analysis of our work and their useful comments. In the following, we explain why we believe that even though the phenomenology of chiral, stable quasiparticles has been established in Floquet realizations such as the PXP cellular automaton, our findings are conceptually different and novel. At the same time, we fully agree that the discussion and comparison between these settings should be made explicit and that prior results in Floquet dynamics must be properly credited. For this reason, we have added a dedicated section in the supplement that discusses the similarities and differences in detail, a discussion in the main text, and new numerical data.

The existence of chiral, stable quasiparticles in PXP-type models is, however, already well established in the literature, particularly in the context of Floquet realizations such as the PXP cellular automaton, where ballistic propagation, absence of dispersion, and characteristic scattering properties have been studied in depth [PRE 102, 062107]. Within this broader context, the present manuscript makes a meaningful contribution by identifying an interesting and distinct class of soliton-like excitations directly in the time-continuous Hamiltonian, without relying on exact Floquet dynamics ... Nevertheless, much of the phenomenology reported here closely parallels what is already known from Floquet-PXP models. The solitons presented share several defining features with previously studied chiral quasiparticles, including fixed-velocity propagation, the absence of dispersion, and the lack of a nontrivial scattering phase.

The central message of our work is the identification of coherently propagating defects in high temperature states in a *non-integrable*, time-independent system. While conventional expectations predict rapid thermalization, our defects show coherent dynamics for exceptionally long times. This distinguishes our findings from integrable Floquet or cellular-automaton variants, pointing to a novel mechanism of weak ergodicity breaking.

Much of the current literature on non-thermal dynamics in chaotic systems, especially in quantum many-body scars, has focused on spatially homogeneous initial states and their revivals. Intuitively, one would expect that locally deforming the fine-tuned scarred state would destroy the coherent dynamics. Surprisingly, our results highlight that scarred dynamics can protect these defects, and inhomogeneous quenches in chaotic models can reveal qualitatively different non-thermal phenomena. This enlarges the landscape of mechanisms by which thermalization can fail. Our findings can be seen in analogy to the early work on scarred dynamics in Rydberg simulators Ref. [Nature 551, 579-584 (2017)]. The surprising aspect was not that simple product states show periodic revivals, but that they occur in a chaotic many-body Hamiltonian. Since then, quantum many-body scars have received enormous attention, numerous different scarred systems have been found, and various mechanisms have been established that describe them, therefore enriching our understanding of thermalization.

At the same time, we fully agree with the Reviewer that a possible connection between our coherently propagating defects and the solitons discovered in Floquet realizations (such as the PXP cellular automaton) could be interesting and should be discussed in detail. To this end, we added a dedicated paragraph to the "Discussion" section of the main text and a dedicated section in the supplementary material with additional numerical data to address this relation.

Similarly, the idea of exploiting such chiral excitations for quantum information transport has already been put forward in the Floquet-PXP setting, and prior works have developed these ideas to the point of proposing universal quantum computing schemes based on such dynamics [PRL 131, 170601]. From this perspective, the information-transport protocols presented in the manuscript appear conceptually very close to existing proposals, differing mainly in their realization within the time-continuous PXP model.

The intention of our information-transport protocol was not to propose a novel, universal model for

quantum computation as in Ref. [PRL 131, 170601], but rather to provide a proof of principle that we are able to coherently transmit information in a chaotic system.

While quantum many-body scars may offer opportunities for state preparation, they have so far yielded few concrete applications as a form of weak ergodicity breaking. By contrast, our results identify an exponentially large manifold of non-thermal, yet controllable, states, which could provide a more versatile resource, for instance, for protocols of the type we demonstrate here, or, more broadly, as a mechanism for unusual transport phenomena in realistic chaotic systems.

That said, we fully concur with the Reviewer that our mechanism for quantum information transmission is similar in spirit to Ref. [PRL 131, 170601]. We apologize for not making this connection explicit, as we were not aware of this work at the time of submission. We have now clarified the relationship in the revised manuscript and added the appropriate citation.

While the authors emphasize experimental relevance, the manuscript does not explicitly investigate the stability of the proposed solitons under more realistic Rydberg Hamiltonians beyond the PXP approximation, making it difficult to assess their practical observability.

We thank the reviewer for pointing this out, and we have added an additional section in the supplement that discusses the practical implementation of our findings in more detail and provides additional data that shows the practical observability of our quasi-solitons under time-evolution of a more realistic Rydberg Hamiltonian and extended the discussion in the "Discussion" section of the main text.

The additional connection drawn to classical TDVP trajectories is appealing and represents one of the more original aspects of the paper.

We appreciate that the Reviewer finds the connection to classical TDVP trajectories appealing, and we want to note that we have augmented this part with additional discussions in the main text and simulations shown in the supplement.

Response to Reviewer #4

We thank the Reviewer for their careful reading of our manuscript and for recognizing our results as very interesting and timely.

1, Not every particles that move unidirectionally can be called soliton. For example, the fracton also has got this feature. A formal definition of soliton should be a stable solution of a nonlinear equation. Although there is an Eq. 9, it has not exact solution up to date. Instead, the authors may consider domain wall, exciplex, or something like that.

We are grateful to the Referee for their suggestion of alternative names. We carefully considered alternative options, and deemed exciplex to be too exotic and domain wall somewhat long, as our defects should be viewed as a "bound pair of domain walls".

To be precise and prevent any confusion, we have renamed them *quasi*-solitons in the title of our manuscript and added a discussion of this subtlety in the "Introduction" and "Discussion" sections, where we explain the terminology and emphasize that establishing a definitive connection to solitons remains an open question.

2, The time evolution should be calculated to longer duration to exhibit the complete decay of the excitations, such that one can estimate the lifetime.

Our study relies on tensor-network methods to access sufficiently large system sizes. The reachable evolution times are therefore limited by the growth of entanglement, which in practice requires an exponential increase of the bond dimension with simulation time. Given the exceptionally long lifetime of the quasi-solitons, it is computationally infeasible to evolve the system to times where they are fully decayed without using exact diagonalization or Krylov time evolution methods, which store the entire wave function. This will limit the accessible system sizes and introduce finite-size effects at even shorter times than we can currently study. However, we have added the section "Soliton stability and collisions" to the main text in which we estimate the lifetime of the solitons by analyzing the exponential decay rate of the translation fidelities.

3, Using different colors to denote S, L and R state is not clear. As the right two sites are in XY plane in S state, the authors may consider to use two horizontal arrows for S. And those for L and R could be up-down symmetric.

We thank the Reviewer for pointing out this potential ambiguity. However, visualizing the two entangled spins of the seven different cells ($|S, L, R\rangle, |L^{\pm}\rangle, |R^{\pm}\rangle$) used in the manuscript via two-dimensional arrows in a meaningful way is challenging. Indeed, the orientation of these spins is not confined to a plane but explores all three dimensions of the Bloch sphere. To underline this, we have calculated the projections onto the x, y, and z axes of both spins of the entangled pair for each cell:

	spin 2			spin 3		
state	$\langle\sigma^x\rangle$	$\langle\sigma^y\rangle$	$\langle\sigma^z\rangle$	$\langle\sigma^x\rangle$	$\langle\sigma^y\rangle$	$\langle\sigma^z\rangle$
$\|S\rangle$	0.00	0.00	−0.41	0.00	0.00	+0.41
$\|L\rangle$	0.00	+0.84	−0.30	0.00	−0.54	−0.70
$\|R\rangle$	0.00	−0.84	−0.30	0.00	+0.54	−0.70
$\|R^+\rangle$	+0.39	−0.42	−0.35	+0.59	+0.27	−0.15
$\|R^-\rangle$	−0.39	−0.42	−0.35	−0.59	+0.27	−0.15
$\|L^+\rangle$	+0.39	+0.42	−0.35	+0.59	−0.27	−0.15
$\|L^-\rangle$	−0.39	+0.42	−0.35	−0.59	−0.27	−0.15

Local Pauli projections $\langle \sigma_j^\alpha \rangle$ on spins $j = 2, 3$.

Although the table above has several repeating expectation values, in general, projections of spins are subtly different, and it is hardly feasible to show them in a consistent way. As a result, we believe that adding more details to our representation will not add much meaningful information but will instead lead to more clutter, making it harder to understand. Therefore, we are of the opinion that keeping the current color-labeling is the best option. Nevertheless, to improve the clarity of our color-labeling scheme, we have extended the description of the cell legend in the caption of Fig. 1. Additionally, we have added a sketch that shows how the energy-soliton cells are constructed (Fig. 4 **a** in the updated manuscript).

4, Zi has not got definition, though it is obvious for experts.

We thank the Reviewer for spotting this omission, and we added the corresponding definition.

5, Why does the collision change the energy without phase shift, considering the system is closed?

The total energy of the system is, of course, conserved throughout the collision. However, during the collision, a fraction of the energy is scattered away from the linear soliton trajectory. This results in partial attenuation of the soliton, with no appreciable phase shift visible in its trajectory. This behavior is shown in Fig. S6.

6, How to map theta back to the quantum state? Some superposition coefficients?

The mapping between the parametrization angles of the onsite matrices and the corresponding quantum state is non-trivial and given by the definition of the MPS. While we have defined the parametrization of the onsite matrices of our Ansatz (given in Eq. 11 in the "Methods" section of the updated manuscript), we have not explicitly defined the form of the infinite-chain MPS state obtained from this Ansatz. We are grateful to the Reviewer for pointing out this omission, and we have added it in the "Methods" section (Eq. 12 of the updated manuscript).

Response to Reviewer #1

We are very grateful to the Reviewer for their helpful comments, which have allowed us to improve the manuscript. We also appreciate their positive assessment that our results represent a significant new finding in the dynamics of the extensively studied PXP model and merit publication in Nature Communications.

In the added phrase "with $Z_i = |\uparrow\rangle\langle\uparrow| - |\downarrow\rangle\langle\downarrow|$" below Eq (2), they should indicate that this operator acts on site i.

We appreciate the Reviewer pointing out this oversight on our part. We clarified the definitions of the local operators by indicating that these operators act on site i. Specifically, we replaced "$Z_i = |\uparrow\rangle\langle\uparrow| - |\downarrow\rangle\langle\downarrow|$" by "$Z_i = (|\uparrow\rangle\langle\uparrow| - |\downarrow\rangle\langle\downarrow|)_i$", and made analogous changes for the operators X_i and P_i.

The authors claim that there is a comment on the relation between the $|K\rangle$ and $\mathbb{Z}_3$ states in the "Model and properties" section of the main text and in S2B of the supplement. We cannot find such a comment that explicitly mentions the relation to the $\mathbb{Z}_3$ state.

We have now explicitly added a comment on this relation in the "Model and properties" section of the main text. The relevant sentence now reads: "To obtain coherently propagating chiral excitations in the PXP model, we focus on a different scarred 3-site periodic initial state that was obtained in earlier works using semiclassical trajectories [24] and an approximate su(2) algebra [29] (which explains previously reported revivals of the product state $\mathbb{Z}_3 = |{\downarrow\downarrow\uparrow\downarrow\downarrow\uparrow} \ldots\rangle$ [19] through the large overlap between the two states).".

Response to Reviewer #3

We thank the Reviewer for their helpful comments and thorough analysis. We are pleased that the Reviewer thinks that our revisions have strengthened the presentation and helped to place the work in a broader context.

That said, my main concern regarding the level of conceptual novelty remains only partially addressed. The authors emphasize that "the central message of our work is the identification of coherently propagating defects in high-temperature states in a non-integrable, time-independent system." However, the existence of coherently propagating defects in the non-integrable PXP model is, in my view, not new. In particular, related ballistic defect dynamics has already been observed in earlier works [25], where localized excitations propagate on top of scarred backgrounds. While those defects are not chiral in the static PXP model, they have subsequently been connected to chiral quasiparticles in integrable Floquet realizations such as the PXP cellular automaton [34].

From this perspective, the main conceptual advance of the present manuscript appears to be that the defects are already chiral directly in the time-continuous, non-integrable PXP Hamiltonian. While I agree that this is an interesting and nontrivial observation, I do not believe that it constitutes a sufficiently strong conceptual advance beyond the existing literature to meet the novelty standards expected for publication in Nature Communications. Rather, the work seems to establish that a phenomenology closely related to that of Floquet-PXP quasiparticles can already emerge in the static model, which I view as an important extension, but not necessarily a fundamentally new mechanism.

We respectfully disagree with the Reviewer's assessment. We believe that the chirally propagating quasiparticles identified in our work are conceptually distinct from previous findings. In particular, they arise in chaotic continuous-time models relevant to Rydberg atom chains at high energy densities, where information from local deformations of fine-tuned scarred trajectories would normally be expected to scramble, therefore, further challenging our understanding of thermalization in quantum many-body systems. By contrast, solitons in integrable systems are more natural, and

their appearance in the PXP cellular automaton setting is therefore less surprising.

Compared with previously observed coherently propagating defects in the continuous-time PXP model [25], the chirally propagating quasiparticles identified in our work lead to several consequences and phenomena. In particular, our quasi-solitons give rise to an exponentially large manifold of non-thermal, experimentally accessible states. This extends the usual picture of weak ergodicity breaking in chaotic systems, where coherent non-thermal dynamics is typically associated with only a small number of special initial states or scarred eigenstates. Moreover, these quasi-solitons can ballistically transport energy and quantum information, raising possible connections to the unusual energy transport observed in the PXP model [15] and suggesting that such quasiparticles may more broadly lead to unconventional transport phenomena in quantum many-body systems.

Finally, we show that similar coherent structures also emerge in a classical nonlinear dynamical system, establishing an interesting connection between soliton-like objects in chaotic quantum many-body dynamics and classical nonlinear systems, where such structures have been studied extensively. More generally, at a time when quantum simulators are becoming increasingly powerful, our results suggest that inhomogeneous quenches provide a promising route to uncover new forms of non-thermal dynamics beyond homogeneous scarred trajectories. We therefore believe that our work will stimulate further exploration of inhomogeneous non-thermal phenomena in many-body systems, potentially uncover connections between quantum many-body dynamics, classical nonlinear systems, and transport phenomena. In our view, Nature Communications is an ideal venue for this work, as it can bring these results to a broad audience across these different areas.

Response to Reviewer #4

We thank the Reviewer for their thorough analysis of our work and for the helpful comments that allowed us to improve the manuscript. We are pleased that all of their concerns have been addressed and that they recommend publication.

Summary of changes: In the following, we summarize the changes made to the manuscript since the last revision. These changes result from the incorporation of the reviewer comments, the adaptation of the manuscript to the formatting requirements of Nature Communications, and a final proofreading of the text. Broadly, they include a slight reorganization of the headings, the addition of the corresponding authors' email addresses, the inclusion of author contributions and competing interests sections, and the adaptation of the reference style. Furthermore, we updated all figures to comply with the formatting requirements of Nature Communications, including the requested data types, resolution, colors, and labeling. These updates were restricted to presentation and formatting aspects and did not alter the quantities shown or the scientific content of the figures.

Beyond the formatting adaptations, the following changes were made:

Main text:

- We revised the abstract by changing "Solitons — localized wave packets that travel without spreading — play a central role in understanding transport and properties of nonlinear systems, from optical fibers to fluid dynamics." to "Solitons — localized wave packets that travel without spreading — play a central role in understanding transport and properties of nonlinear systems.". Additionally, we changed "In addition to a phenomenological description of solitons, we identify their counterpart in a classical nonlinear dynamical system obtained from a variational projection of the quantum dynamics. We demonstrate the potential use of solitons in quantum information transfer and conjecture their relevance for the anomalous energy transport reported in numerical studies of Rydberg atom arrays." to "In this work, in addition to a phenomenological description of solitons, we identify their counterpart in a classical nonlinear dynamical system, demonstrate their potential use in quantum information transfer, and conjecture their relevance for anomalous energy transport reported in numerical studies of Rydberg atom arrays." Now the abstract length is below 150 words and the last sentence starts with "In this work" as required by the guidelines.
- In the Introduction we replaced "a novel type" with "a form".
- In the Introduction, we clarified the correspondence between the local observables and the panels of Fig.1 by changing "the energy density and number operator density" to "the number operator density (**a**) and energy density (**b**)".
- We corrected the incomplete sentence "establishing a rigorous will be a subject of future work" to "establishing a rigorous connection will be a subject of future work".
- In the introduction we changed "via local observables - the energy density and number operator density - showing" to "via local observables — the energy density and number operator density — showing".
- We clarified the definitions of the local spin operators and projector by explicitly indicating that these operators act on site i. Specifically, we replaced $Z_i = |{\uparrow}\rangle\langle{\uparrow}| - |{\downarrow}\rangle\langle{\downarrow}|$ by $Z_i = (|{\uparrow}\rangle\langle{\uparrow}| - |{\downarrow}\rangle\langle{\downarrow}|)_i$, $X_i = |{\downarrow}\rangle\langle{\uparrow}| + |{\uparrow}\rangle\langle{\downarrow}|$ by $X_i = (|{\downarrow}\rangle\langle{\uparrow}| + |{\uparrow}\rangle\langle{\downarrow}|)_i$, and $P_i = |{\downarrow}\rangle\langle{\downarrow}|$ by $P_i = (|{\downarrow}\rangle\langle{\downarrow}|)_i$.
- In the section Model and properties we changed $|\ldots\downarrow\uparrow\downarrow\underline{\downarrow}\downarrow\uparrow\downarrow\ldots\rangle$ to $|\mathbb{Z}_2\rangle$ state, $|\ldots\downarrow\uparrow\downarrow\downarrow\downarrow\uparrow\downarrow\ldots\rangle$
- We expanded the paragraph introducing the scarred three-site periodic initial state of the PXP model by adding the clarification "which explains previously reported revivals of the product state $|\mathbb{Z}_3\rangle = |\downarrow\downarrow\uparrow\downarrow\downarrow\uparrow\ldots\rangle$ [19] through the large overlap between the two states".
- In Eq. (3), we corrected the tensor-product definition of $|K\rangle$ by changing the lower bound of

the product index from $k = 0$ to $k = 1$, so that the product contains exactly $N/3$ three-site unit cells rather than $N/3 + 1$ cells.

- In the section Soliton excitations we changed "therefore representing a novel class of non-ergodic behavior in highly excited states." to "therefore representing a distinct class of non-ergodic behavior in highly excited states."
- In the Discussion section we changed "dynamics in excited quantum systems and novel realization of soliton physics in quantum simulators" to "dynamics in excited quantum systems and realizations of soliton physics in quantum simulators"
- In the section Energy transport via solitons we changed "can be used to construct novel homogeneous scarred initial states" to "can be used to construct different homogeneous scarred initial states"
- After Eq. (10), we added the sentence "Here, the reduced operator obtained after tracing out $\bar{A}$ is understood to be embedded back into the full Hilbert space by tensoring with the identity on $\bar{A}$" to clarify how the reduced operator acts in the full-chain expectation value.
- In Eq. (12), we corrected the infinite-MPS definition by changing the product range from $i = 0, \ldots, \ell$ to $i = 1, \ldots, \ell$ and by replacing the unit-cell indices $\theta_{i \bmod N}$ and $\phi_{i \bmod N}$ with $\theta_{((i-1) \bmod N)+1}$ and $\phi_{((i-1) \bmod N)+1}$. This makes the tensor-product indices consistent with the summed physical indices $\sigma_1, \ldots, \sigma_\ell$ and avoids an undefined zeroth unit-cell parameter.
- In the equations of motion, we replaced all occurrences of the dot notation $\dot{\phi}$ and $\dot{\theta}$ by $\partial_t \phi$ and $\partial_t \theta$ to use a uniform derivative notation throughout.
- We changed the subsection title "TEBD" in Methods to "Time-evolving block decimation algorithm". In the same subsection we changed "Unitary time-evolutions in this article have been conducted via the TEBD algorithm" to "Unitary time-evolutions in this article have been conducted via the time-evolving block decimation (TEBD) algorithm".
- In the Discussion section we changed "by nonlinear equations of motion derived via the time-dependent variational principle (TDVP)." to "by nonlinear equations of motion derived via TDVP.". In the section Model and properties we changed "Notably, perturbing the fine-tuned scarred initial states away from the semiclassical TDVP trajectory destroys" to "Notably, perturbing the fine-tuned scarred initial states away from the semiclassical time-dependent variational principle (TDVP) trajectory destroys". Furthermore, we changed the subsection title in Methods from "TDVP" to "Time-dependent variational principle"
- We clarified that the symbols $\mathbb{Z}_2$ and $\mathbb{Z}_3$ refer to quantum states by replacing occurrences of $\mathbb{Z}_2$ and $\mathbb{Z}_3$ with $|\mathbb{Z}_2\rangle$ and $|\mathbb{Z}_3\rangle$, respectively (also in the Supplementary Material)
- We removed all appearances of the word "remarkable" in the main text of the manuscript. In the subsection Soliton excitations, we changed "Remarkably, a single unit cell" to "A single unit cell" and "Remarkably, by the full period" to "By the full period". In the Discussion section we changed "We want to highlight how remarkable the coherent transport" to "We want to highlight how unusual the coherent transport".
- The funding in the section Funding Statement has been updated compared to the previously listed funding in the Acknowledgments section.
- We changed certain words that were written in italic to normal font

Supplementary Material:

- The section structure has been updated. Instead of sections and subsections, the Supplementary Information has been divided into 7 Supplementary Notes in compliance with the formatting requirements.
- The text "This Supplementary Material is organized in three sections. The first section provides additional details for quantum dynamics of solitons, including their internal structure, collisions, and entanglement dynamics, as well as spectral decomposition of initial states with solitons. The second section provides details on the TDVP description of the PXP model and discusses solitons in the resulting system of first-order nonlinear differential equations. Finally, the last section illustrates existence of the decaying solitons in the unitary dynamics of the PPXPP model, that generalizes the PXP model to approximately describe longer range Rydberg blockade." has been removed
- We removed the undefined reference to Fig. S4 by changing "In Fig. S4 we demonstrate" to "In the main text, we demonstrated".
- We removed a previously empty Fig. S4 from the Supplementary Information.
- We corrected the typo "by the lack of peas" to "by the lack of peaks".
- In Eq. (S5), we corrected the undefined index i to j in the intermediate states $|K^{\{N-j+1\},\varnothing}\rangle$ and $|K^{\{N-j\},\varnothing}\rangle$, making the equation consistent with the initially introduced defect position j.
- We corrected the figure reference in the discussion of the entanglement dynamics by changing the reference from Fig. S6 to Fig. S4.
- In the discussion of Fig. S7, we corrected the relevant momentum sectors by changing both "$k = 0$ and $k = \pm\pi/3$" and "$k = 0$ and $k = \pm 3/N$" to "$k = 0$ and $k = \pm\frac{2\pi}{3}$".
- We made the notation for the shifted loss contribution consistent by replacing both occurrences of L_{shift} with L_{shifted}.
- We removed the undefined cutoff parameters d_c and D_c in the loss functions by replacing them directly with their numerical values, using $d/0.4$ in P_{closed} and $D/0.7$ in P_{shifted}.
- In the equations of motion, we replaced all occurrences of the dot notation $\dot{\phi}$ and $\dot{\theta}$ by $\partial_t\phi$ and $\partial_t\theta$ to use a uniform derivative notation throughout.
- We corrected the typo "under unitary time-evolution of the quantum mode" to "under unitary time-evolution of the quantum model".
- We corrected the numerical tuple by adding the missing commas in $(4.13244625, 3.59559592, 6.24284442)$.
- We corrected the phrase "the defects looses its chirality" to "but the defect loses its chirality".
- We corrected the incomplete caption phrase "with a somewhat faster under evolution of the Rydberg Hamiltonian" to "with a somewhat faster decay under evolution of the Rydberg Hamiltonian".
- When referring to "EOMs (13) and (14)" of the main text, and it was not unambiguously clear that we refer to the equations of the main text (and not the supplement), we modified the reference to "EOMs (13) and (14) of the main text" and analogously when referring to Eq. 1, Eq. 5, Eq. 11, and Eq. 15 of the main text.
- In the definition of $|K_5\rangle$, we corrected the tensor-product range by changing the lower bound from $k = 0$ to $k = 1$ and the upper bound from $N/3$ to $N/5$, so that the product contains the intended number of five-site unit cells.
